# Essential oil-derived decomposable polymers via cycloaddition polymerization of silyl ether-linked phenylpropanoids

**DOI:** 10.1038/s41467-025-65707-x

**Published:** 2025-11-27

**Authors:** Ryo Nagaya, Tatsuya Seko, Kazuhiro Okamoto, Kazuhide Ueno, Mahito Atobe, Naoki Shida

**Affiliations:** 1 Department of Chemistry and Life Science, Yokohama National University, Yokohama, Japan; 2 Department of Science, University of Toyama, Toyama, Japan; 3 Institute of Advanced Sciences, Yokohama National University, Yokohama, Japan; 4 PRESTO, Japan Science and Technology Agency (JST), Saitama, Japan

**Keywords:** Polymer synthesis, Photocatalysis, Design, synthesis and processing

## Abstract

Owing to increasing concerns regarding climate change, research concerning the use of plant biomass as a renewable carbon resource has become increasingly active. Phenylpropanoids, which are aromatic compounds derived from plants, offer renewable sources owing to their availabilities and structural diversity. This study presents an approach for use in producing decomposable polymers with high biomass contents via [2 + 2] cycloaddition polymerization. Bifunctional monomers with silyl ether-linked phenylpropanoids were synthesized, and their polymerizations were investigated using chemical and electro- and photochemical methods. The resulting polymers contained aromatic and cyclobutane rings and silyl ether bonds in their backbones, which enhanced their thermal properties. Notably, these polymers could be decomposed via Diels-Alder reactions at the cyclobutane rings or Si–O bond cleavage, facilitating chemical re- and upcycling. Here, we show a sustainable method of producing high-biomass decomposable polymers, potentially contributing in reducing plastic waste and promoting a circular economy.

## Abstract (web summary)

Phenylpropanoids are an emerging class of bio-based aromatic monomers. Here, the authors report [2 + 2] cycloaddition polymerisations of silyl ether-linked phenylpropanoids to yield polymers capable of decomposing via Diels-Alder reactions or Si-O cleavage, enabling their chemical recycling.

## Introduction

The efficient use of renewable plant biomass resources in polymeric materials is urgently required to halt anthropogenic carbon emissions from petroleum-derived raw materials^1–5^. Biomass resources derived from wood and plants display potential as sufficient carbon sources to replace raw petroleum materials because of their levels of abundance. Phenylpropanoids are natural aromatic organic compounds extracted from plants, such as anise, ylang-ylang, and clove, as essential oils. Because of their availabilities and structural diversity, they are widely used in spices, cosmetics, and pharmaceutical derivatives^6–9^, and the worldwide market for essential oils reached USD 10 billion in 2021^10^. In addition, considerable efforts have been devoted to generating phenylpropanoids directly or indirectly from inedible woody biomass, such as lignin, facilitating its use as a renewable source of raw materials^11–14^.

**Fig. 1:**
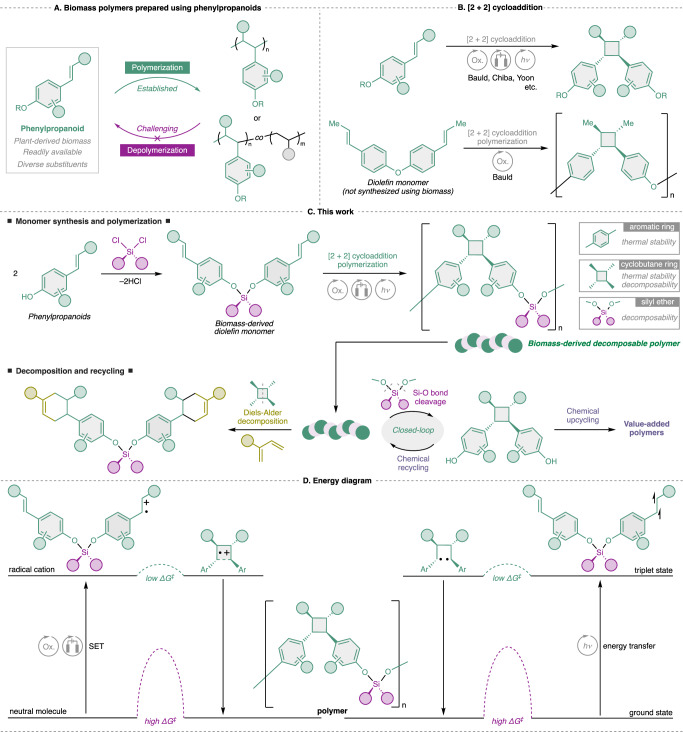
Concept of this work. **A** Previous studies regarding the conversion of biomass-derived phenylpropanoids to polymer materials. **B** Precedents of [2 + 2] cycloaddition and polymerization as a method of efficiently polymerizing phenylpropanoids. **C** Strategy applied in synthesizing biomass-derived decomposable polymers using phenylpropanoids in this study. **D** Energy diagram for [2 + 2] cycloaddition polymerization.

In this context, the polymerization of phenylpropanoids has been extensively investigated for decades. The reactivities of the vinyl groups of phenylpropanoids are generally low owing to the steric hindrance of their 1,2-disubstituted frameworks. Therefore, the homopolymerization of phenylpropanoids has been a long-standing challenge in this field, and their copolymerization with other reactive monomers, such as styrene and methyl acrylate, has been widely studied^15–18^. Recently, progress was reported in the homopolymerization of phenylpropanoids^19–23^, generating polymers with high contents of biomass-derived components. However, even with state-of-the-art polymerization techniques, only a handful of polymers with high phenylpropanoid contents, e.g., >65 wt.% phenylpropanoids, have been reported. In addition, most reported phenylpropanoid-containing polymers exhibit polystyrene-type backbones polymerized via addition at the vinyl moiety, which limits the applicability and recyclability of phenylpropanoids (Fig. 1A). Notably, Labrie-Cleary et al. recently reported the synthesis of photodegradable polymers via the homopolymerization of *o*-hydroxycinnamic acid. However, the resulting material faces limitations in chemical recyclability, as the decomposition products are not readily reusable^23^.

To develop decomposable polymers with high phenylpropanoid contents, [2 + 2] cycloaddition reaction represents a promising strategy, as the reactivity of multiply substituted olefins in this transformation is well established^24^. Since [2 + 2] cycloaddition reactions hardly proceed under thermal conditions, they are generally induced via single electron transfer (SET)-triggered reactions using chemical reagents^25–28^, electrolysis^29–31^, and photoredox catalysis^32–34^, and the photochemical reactions based on energy transfer using photosensitizers (Fig. 1B)^35–37^. Indeed, Bauld et al. reported the [2 + 2] cycloaddition polymerization of diolefins bearing electron-rich aromatic rings via SET-triggered hole-catalytic cycloaddition (Fig. 1B)^38,39^. Although their study introduced a unique polymerization mechanism, the polymers reported by them were neither derived from biomass resources nor designed to be chemically decomposable, thereby limiting their relevance to sustainable polymer development. Regarding the photocatalytic cycloaddition pathway, Oshimura et al. reported the synthesis of a bifunctional cyclobutane-containing monomer from a caffeic acid derivative via [2 + 2] cycloaddition reaction and subsequently reacted it with tetraethylene glycol to produce biomass-derived polyester^40^. Although they have also succeeded in hydrolysis of the resulting polyester, strong acidic or basic conditions and/or heating are typically required for hydrolysis. From the perspective of environmental impact, it seems necessary to design polymers that can be decomposed under milder conditions.

In this study, we report phenylpropanoid-containing polymers with high biomass contents and decomposability via [2 + 2] cycloaddition polymerization (Fig. 1C). Bifunctional monomers containing two phenylpropanoids connected via silyl ether linkages are synthesized, and polymerizations are performed using chemical, electrochemical, and photochemical methods. The polymer design comprises aromatic, cyclobutane, and silyl ether groups in the main chain. The aromatic rings improve the mechanical and thermal properties, whereas the cyclobutane groups provide a high thermal stability due to the thermally unfavorable nature of the retro-[2 + 2] reaction. The Si–O bonds of the silyl ether linkages display higher bond dissociation energies (108 kcal mol^−1^) than that of the C_sp3_–C_sp3_ bond (83 kcal mol^−1^), rendering the polymer chemically and thermally stable. In addition to their robustness, the polymers developed in this study exhibit unique dual decomposition capacities under mild conditions (Fig. 1C). A radical cationic cyclobutane ring is known to be in equilibrium with two olefins, the precursor of the [2 + 2] cycloaddition^32,41^. Therefore, the cyclobutane ring enables a hole-catalytic Diels-Alder reaction in the presence of a diene, enabling an unprecedented mode of polymer decomposition^32^. In addition, the polymers reported herein can be decomposed via reaction with fluoride anions at the Si–O bonds to yield bisphenol products. The resulting bisphenols can be used in chemical re- and upcycling to produce value-added products^42–44^. In general, [2 + 2] cycloaddition reactions between unactivated substrates have high activation energies and do not proceed. However, by activating substrates by one-electron oxidation or energy transfer, the activation energy is reduced, and cycloaddition reaction proceeds (Fig. 1D). Overall, this study proposes a design concept for use in fabricating biomass-derived decomposable polymeric materials with unique main-chain structures, potentially contributing to the sustainable, circular use of carbon resources.

## Results

### Monomer synthesis

First, we attempted to synthesize monomers comprising bisphenylpropanoids connected by silyl ether bonds. The condensation reactions of the phenylpropanoids and dialkyldichlorosilanes were performed at 25 °C in the presence of triethylamine as the base and a catalytic amount of 4-dimethylaminopyridine. We first examined the influences of the substituents on the silicon atom (Table S1). Sterically unhindered substituents lead to destabilization of the resulting monomers, whereas sterically hindered dialkyldichlorosilanes did not react with phenylpropanoids. The reaction between the phenylpropanoids and dichlorodiisopropylsilane proceeded smoothly, affording the desired stable monomers **M1**–**M6** in high yields (Fig. S1).

### Hole-catalytic polymerization using a chemical oxidant

The silyl ether linkages of **M1–M6** increase the electron densities in the aromatic rings because of the small electronegativity of the silicon atoms compared to carbon and oxygen atoms, and thus, these monomers may be compatible with the hole-catalytic cycloaddition polymerization initiated via SET. The hole-catalytic cycloaddition polymerization explored by Bauld et al. mostly relied on the use of a catalytic amount of the chemical oxidant magic blue (MB). Thus, we first examined the polymerization of **M1** using MB.

MB addition to a solution of **M1** resulted in an immediate color change from blue to orange, suggesting a smooth SET between MB and **M1**. However, reprecipitation afforded a significant amount of an insoluble gel. A small amount of the soluble fraction was subjected to gel permeation chromatography (GPC), which suggested the formation of a polymer of >100 kDa (Fig. S5). Thus, MB induced cross-linking via cationic polymerization at the vinyl groups, in addition to the desired cycloaddition polymerization (Table S3), as observed for specific monomers in a previous study^45^.

**Fig. 2:**
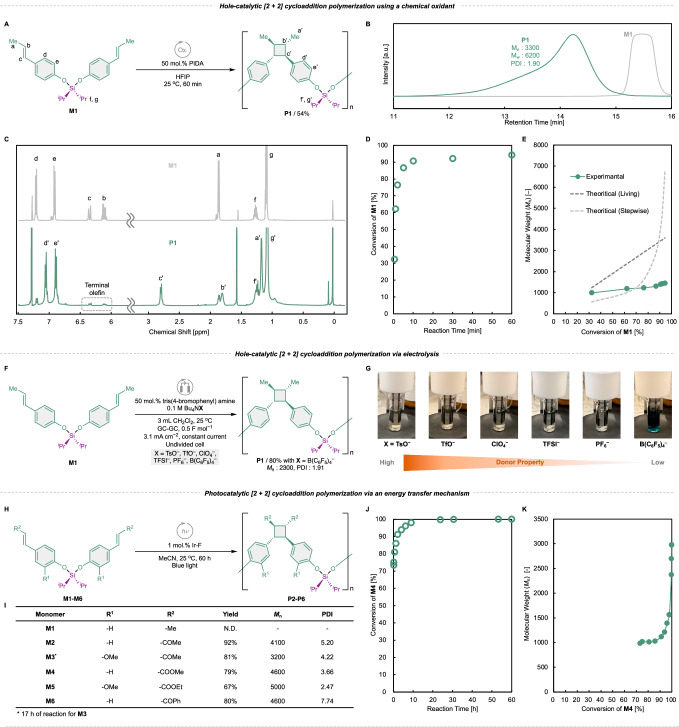
[2 + 2] cycloaddition polymerizations of the monomers. **A** Schematic of hole-catalytic polymerization via chemical oxidation using 50 mol.% PIDA as a chemical oxidant. **B** GPC traces (in THF) and **C** ^1^H NMR spectra (in CDCl_3_) of **M1** and **P1**. **D** Time course of conversion using 0.1 M **M1**, as initiated by PIDA. **E** Relationship between the conversion of **M1** and number-average molecular weight (*M*_n_) of **P1**. The green line represents the experimental data, and the dark/light gray dotted lines represent the theoretical lines of living/stepwise polymerization, respectively. **F** Schematic of hole-catalytic polymerization via electrochemical oxidation using 50 mol.% tris(4-bromophenyl) amine as a redox mediator (TsO^−^ = *p*-toluenesulfonate, TfO^−^ = trifluoromethanesulfonate, TFSI^−^ = bis(trifluoromethanesulfonyl)imide). **G** Images of the solutions with different electrolytes during electrolysis. **H** Schematic of photocatalytic [2 + 2] cycloaddition polymerization via energy transfer. **I** Results of photocatalytic [2 + 2] cycloaddition polymerization using the monomers. **J** Time course of conversion using 0.77 M **M4**. **K** Relationship between the conversion of **M4** and *M*_n_ of **P4**. Me methyl, Et ethyl, Ph phenyl.

Inspired by recent progress in hole-catalytic [2 + 2] cycloaddition reactions using hypervalent iodine in small-molecule synthesis^26,46^, we changed the oxidant from MB to iodobenzene diacetate (PIDA). **M1** was polymerized by adding 50 mol.% PIDA in 1,1,1,3,3,3-hexafluoro-2-propanol (HFIP) at 25 °C (Fig. 2A). After purifying the crude product via reprecipitation, **P1** was obtained as a white powder in a 54% yield without the formation of an insoluble product. GPC revealed that the number- (*M*_n_) and weight-average molecular weights (*M*_w_) of **P1** were 3300 and 6200, respectively, and its polydispersity index (PDI) was 1.90 (Fig. 2B). Comparing the ^1^H nuclear magnetic resonance (NMR) spectra of **M1** and **P1** suggested that the signals representing the olefin protons of **M1** were mostly diminished after the reaction. This is concomitant with the manifestation of the signals representing methine protons, which are characteristic of the cyclobutane rings of **P1** (Fig. 2C). In addition, the signals representing the terminal olefin protons of **P1** were observed at the same positions as those of **M1** ($\delta$ = 6.0–6.5). The molecular weight of **P1** estimated from the ^1^H NMR was 3600 Da, corresponding to *ca*. 9.5 repeating units, showing a good agreement with the results of GPC measurement (Fig. S15). From these data, it is suggested that **M1** polymerization proceeds via the formation of cyclobutane rings via [2 + 2] cycloaddition to yield **P1**. The content of biomass-derived ingredients in **P1** is 69 wt.%, which is higher than most of the reported polymers synthesized from phenylpropanoid by copolymerization with other non-biomass vinyl monomers.

To gain insight into the polymerization mechanism, the relationship between the conversion of **M1** and *M*_n_ was investigated. A time-trace experiment revealed that 62% of **M1** was consumed within 1 min of PIDA addition (Fig. 2D). Furthermore, *M*_n_ exhibited almost no correlation with **M1** conversion (Fig. 2E). These data strongly suggest that the reaction proceeded via a chain-growth mechanism with a termination reaction. Intramolecular transfer of a radical cation on the cyclobutane ring after [2 + 2] cycloaddition reaction or back electron transfer from a neutral monomer or polymer would lead to a propagating reaction, but other termination reactions would occur during the reaction. It should be noted that both **M1** and **P1** can potentially be involved in the propagating reaction by SET, since there was little difference in their oxidation potentials (Fig. S3).

To further investigate the competition between chain propagation and termination reactions, we performed polymerization of **M1** with various conditions. The concentration of **M1** had minimal impact on the molecular weight of **P1** (Tables S4 and S5), whereas increasing the equivalents of PIDA significantly raised the molecular weight (Tables S5–S7). This is presumably because increasing amount of PIDA promoted reoxidation of the polymer chain ends even after swift termination reaction. The plausible mechanism of hole-catalytic polymerization based on these findings is presented in Fig. S10.

Contrasting to the result of **M1**, the polymerizations of the other monomers (**M2–M6**) hardly progressed under these conditions (Table S14). Cyclic voltammetry (CV) study of **M1–M6** revealed that the oxidation potentials of **M2–M6** are higher than that of **M1** (Fig. S2), suggesting that the alkene moieties with electron-withdrawing groups, such as ketone or ester, render the monomers less susceptible to SET oxidation. The order of the oxidation potentials of **M1–M6** matched with the order of HOMO energy levels estimated by DFT calculations (Table S2).

### Hole-catalytic polymerization via electrolysis

We then investigated electrochemical hole-catalytic cycloaddition polymerization. First, the electrochemical polymerization of **M1** was performed via direct oxidation on the anode surface. However, a passivation film was formed immediately, which hampered further electrolysis. To avoid passivation, electrolysis was performed using the redox mediator tris(4-bromophenyl) amine (Fig. 2F). Remarkably, the progress of the polymerization reaction was sensitive to the nature of the anionic species in the supporting electrolyte. The use of the weakly coordinating anion B(C_6_F_5_)_4_^−^ was strikingly effective, and the desired polymer was obtained in an 80% yield (Table S13). When Bu_4_NB(C_6_F_5_)_4_ (Bu = *n*-butyl) was used as the supporting electrolyte, the color of the reaction solution remained blue, which was derived from the radical cationic state of the mediator, whereas more coordinating electrolytes turned the solution orange (Fig. 2G). This suggests that the weakly coordinating electrolyte solution keeps radical cationic cyclobutane intact^47^, and back electron transfer from the mediator is promoted. It is noteworthy that the use of tris(4-bromophenyl) amine mediator successfully afforded the desired polymer by electrolysis, even though the aforementioned hole-catalysis by using MB induced uncontrolled cationic polymerization. This is presumably due to the slow generation of oxidant by electrochemical reaction was preferable compared to the rapid addition of oxidant in chemical oxidation reaction.

Meanwhile, the electrochemical polymerizations of **M2–M6** hardly progressed as in the case of chemical oxidants, even in using tris(2,4-dibromophenyl) amine, which has a higher oxidation potential than tris(4-bromophenyl) amine (Figs. S2 and S11). This is presumably due to the lack of nucleophilicity of the monomers and/or difficulty in the back electron transfer, making it difficult to proceed with a hole-catalytic [2 + 2] cycloaddition polymerization.

### Photocatalytic polymerization via energy transfer

Several studies report the [2 + 2] cycloaddition of carbonyl-substituted olefins, including several phenylpropanoids, such as chalcone and cinnamate, under photocatalysis via an energy transfer mechanism^35–37^. Inspired by these precedents, we investigated the polymerizations of **M1–M6** under photocatalytic conditions (Fig. 2H). Blue light was irradiated for 60 h in the presence of 1 mol.% of the (4,4′-di-*tert*-butyl-2,2′-bipyridine)bis[3,5-difluoro-2-[5-(trifluoromethyl)−2-pyridinyl]phenyl]iridium(III) hexafluorophosphate (Ir-F) photocatalyst. No polymer products were obtained using **M1**, whereas the polymerizations of **M2–M6** proceeded to afford the corresponding polymers **P2–P6** in high yields (Fig. 2I). The biomass contents of the resulting polymers are 73–79 wt.%. Based on the previous reports, the successful progress of polymerization for **M2–M6** was attributed to the presence of carbonyl groups adjacent to the olefins, stabilizing the triplet states^36^.

Although the failure of polymerization of **M1** is reasonable due to the absence of a carbonyl group adjacent to the olefin moiety, the polymerization of **M1** via hole-catalytic mechanism with Ir-F as a photoredox catalyst seemed reasonable. However, no polymerization was observed under these conditions as mentioned above. We attributed this outcome to the modest oxidizing power of Ir-F (*E*_1/2_ = +1.21 V vs. SCE)^48^, which is not strong enough to oxidize **M1**. In fact, when we employed [Ru(bpz)_3_][B(C_6_F_5_)_4_]_2_ (bpz = 2,2′-bipyrazine), a photocatalyst with a higher oxidation potential (*E*_1/2_ = +1.45 V vs. SCE)^32,48^, **P1** was successfully obtained via photochemically driven hole-catalytic polymerization of **M1** (Table S8).

We then investigated the mechanism of photocatalytic polymerization using **M4**. The conversion of **M4** exceeded 75% within 10 min of initiating the reaction, indicating a favorable reaction rate (Fig. 2J, Table S21). In contrast, *M*_n_ of **P4** continued to increase even after the conversion of **M4** had plateaued (Fig. 2K), indicating a behavior distinct from that observed in the hole-catalytic polymerization. This relationship indicates that photocatalytic polymerization proceeds via a stepwise mechanism.

**P2–P6** showed notably larger PDI values compared to **P1**. This is presumably due to the difference in polymerization mechanism. Also, the possibility of forming cyclic polymers by [2 + 2] cycloadditions between terminal olefins cannot be ruled out. In fact, most of the terminal olefin protons of photopolymerized polymers are not observed in the ^1^H NMR spectra.

### Thermal properties

**Fig. 3:**
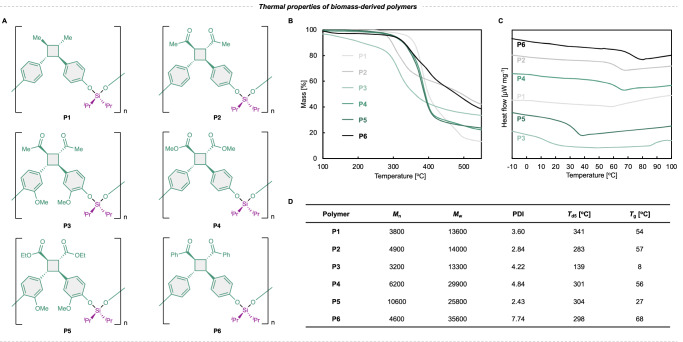
Characteristics of the biomass-derived decomposable polymers. **A** Structures of **P1–P6**. **B** Thermogravimetric analysis (TGA) thermograms of **P1–P6**. **C** Differential scanning calorimetry (DSC) thermograms of **P1–P6**. Heat flow is shown in the endothermic down direction. **D** Thermal properties of each polymer. Polymerization conditions, **P1**: 0.096 mM **M1** in HFIP initiated by 100 mol.% PIDA at 25 °C for 60 min. **P2** and **P4–P6**: 0.384 M monomer in acetonitrile (MeCN) catalyzed by 1 mol.% of the Ir-F photocatalyst at 25 °C for 60 h. **P3**: 0.384 M **M3** in MeCN catalyzed by 1 mol.% of the Ir-F photocatalyst at 25 °C for 17 h.

The thermal properties of the resulting polymers were evaluated using thermogravimetric analysis (TGA) and differential scanning calorimetry (DSC). Their 5% mass loss temperatures (*T*_d5_) are in the range 139–341 °C under a nitrogen atmosphere (Fig. 3A, B, D). The *T*_d5_ generally depends on the substituents, particularly those on the alicyclic structures of the main chain, with the following order of thermal stability: –CH_3_ (**P1**) > –COO (**P4,** **P5**) > –CO (**P2,** **P3,** **P6**).

The polymers prepared in this study exhibit relatively high glass transition temperatures (*T*_g_) in the range 8–68 °C compared to polymers bearing silyl ether groups (Fig. 3A, C, D). The rigid framework formed by including the alicyclic structures within the main chain may improve *T*_g_^49^. In addition, not only the alicyclic structures, but also aromatic rings improve rigidities of the polymers. *T*_g_ was the highest for **P6**, which contains the largest number of aromatic rings in its structure. Silyl ether polymers generally display relatively low *T*_g_ values owing to their flexible silyl ether bonds with high internal degrees of freedom, and *T*_g_ values of <0 °C have been reported^43,44,50^. Additionally, the methoxy group, as a substituent on the aromatic ring, significantly affects *T*_g_. The polymers without methoxy groups on their aromatic rings (**P1,** **P2,** **P4**, and **P6**) exhibit relatively high *T*_g_ values.

The polymers display high thermal stabilities owing to their backbone groups, such as aromatic rings, alicyclic structures, and silyl ether bonds. These results also suggest that the thermal properties of these materials can be tuned by modifying their polymer backbones and substituents. Notably, cyclobutane rings are known for their high distortion energies, and they are thus susceptible to bond cleavage under mechanochemical conditions^51^. The counterintuitively high thermal stabilities should be attributed to the thermally unfavorable [2 + 2] cycloaddition, and thus, the reverse reaction, i.e., the retro-[2 + 2] reaction, is difficult to proceed thermally either.

### Polymer decomposition

**Fig. 4:**
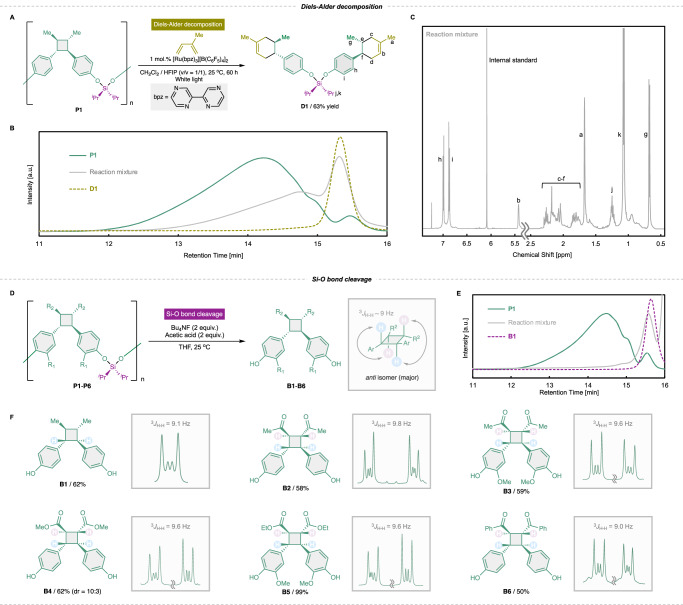
Decomposition reactions of the biomass-derived polymers. The yields were determined via ^1^H NMR spectroscopy of the crude reaction mixtures relative to the internal standard 1,3,5-trimethoxybenzene. Scheme (**A**), GPC traces (in THF) (**B**), and ^1^H NMR spectrum (in CDCl_3_) of the reaction mixture (**C**) of the Diels-Alder decomposition of **P1** using isoprene. Scheme (**D**) and GPC traces (in THF) (**E**) of decomposition via Si–O bond cleavage with Bu_4_NF, and the ^1^H NMR spectra of **B1–B6** (**F**). The coupling constants of the adjacent vicinal protons of the methine groups of **B1–B6** are noted. THF tetrahydrofuran.

Then, decomposability of **P1–P6** was evaluated. First, a decomposition method involving Diels-Alder reactions at the cyclobutane rings was investigated. According to the reaction conditions reported by Yoon et al.^32^, [Ru(bpz)_3_][B(C_6_F_5_)_4_]_2_ and isoprene were added to reaction solution containing **P1**, and the reaction was performed under white light (Fig. 4A). We have also independently synthesized possible Diels-Alder decomposition product, **D1**, for characterization by GPC and ^1^H NMR (see Supplementary Information for details). The GPC trace of the reaction mixture confirmed the decrease in the molecular weight of the polymer, and the formation of small molecular fragment overlapping to **D1** was confirmed (Fig. 4B). ^1^H NMR spectroscopy of the crude mixture after the reaction revealed signals representing the target product **D1**. In addition, the peak of **P1** was not observed in the ^1^H NMR spectrum, indicating that the polymer backbone was completely decomposed (Fig. 4C). The calculated ^1^H NMR yield of **D1** was 63% compared to an internal standard. This decomposition approach has an excellent atomeconomic advantage, and the resulting **D1** is expected to be used as an epoxy resin through epoxidation of olefins^52,53^.

Subsequently, we performed polymer decomposition involving the cleavage of the Si–O bonds at the linker moieties. Fluoride anions can readily cleave silyl ether bonds under mild conditions, driven by the formation of strong Si–F bonds^42,43,54^. Exploiting this property, the decomposition reactions of **P1–P6** were conducted by adding 2 equiv. of Bu_4_NF relative to the numbers of silyl ether bonds of the polymers (Fig. 4D). GPC of the reaction solutions detected no high-molecular-weight species, indicating that the polymer backbones were completely decomposed (Figs. 4E and S20). ^1^H NMR spectroscopy confirmed that the decomposition products of **P1–P6** corresponded to the respective bisphenols **B1–B6**, which were produced in high yields (Fig. 4F).

This decomposition also confirmed the regio- and stereoselectivities of the substituents on the cyclobutane rings. Generally, [2 + 2] cycloadditions produce isomers with different regioselectivities, such as head-to-head or -tail, and stereoselectivities, such as *anti* or *syn*, depending on the reaction method used^55–59^. Regarding the regioselectivity, the coupling pattern of the signals representing the methine protons of the cyclobutane rings changes significantly from head-to-head to head-to-tail. The head-to-head mode exhibits higher-order coupling, whereas the head-to-tail mode does not exhibit such signals^60,61^. The ^1^H NMR spectra of the decomposition products revealed higher-order coupling within **B1–B6**, indicating that head-to-head is the predominant structure of each decomposition product. More significantly, in the case of head-to-head coupling, the coupling constant of the signal representing the methine protons differs depending on the stereochemistry, i.e., *anti* or *syn*. The general coupling constant of the *anti* or *syn* conformation with an adjacent vicinal proton is approximately 9 or 6 Hz, respectively^51,55,56^. The coupling constants of **B1–B6** were in the range 9.0–9.8 Hz, indicating that the *anti*-isomer is predominant in each case (Fig. 4F). The signals representing head-to-head *syn*-type products were also observed in the ^1^H NMR spectrum of **B4**, and the *anti*:*syn* diastereoselectivity was 10:3. In contrast, detailed stereochemical assignments were challenging across the entire polymer series due to significant signal broadening. Nevertheless, within analyzable regions, we observed vicinal coupling constants of approximately *J* ~ 9 Hz (Fig. S17).

### Chemical re- and upcycling

**Fig. 5:**
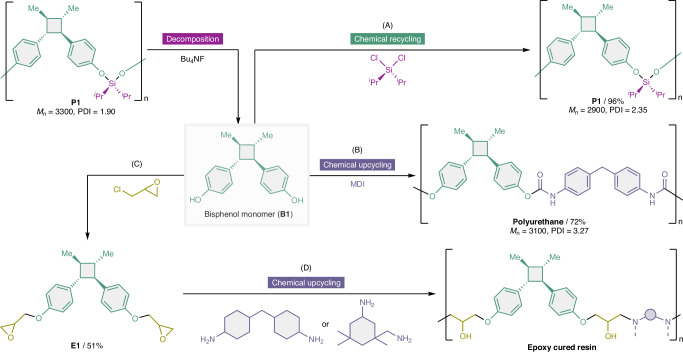
Re- and upcycling of B1. **A** Repolymerization to yield **P1** by adding 1 equiv. of dichlorodiisopropylsilane. **B** Synthesis of polyurethane via reaction with 4,4′-methylenebis(phenyl isocyanate) in the presence of 1,4-diazabicyclo[2.2.2]octane. **C** Epoxidation of **B1** via reaction with epichlorohydrin. **D** Curing reactions of epoxide **E1** with curing agents, such as 4,4′-methylenebis(cyclohexylamine) and isophoronediamine, to yield thermoset networks.

Biomass-derived bisphenols, which are the products obtained via the cleavage of the linker moieties, display potential for use as bifunctional monomers in chemical re- and upcycling. As a proof-of-concept, **B1** was reacted with a stoichiometric amount of dichlorodiisopropylsilane, and condensation polymerization was performed, resulting in the successful formation of recycled **P1** (Fig. 5A). The molecular weight of recycled **P1** was almost identical to that of the original **P1**, demonstrating that the material can be depolymerized and repolymerized without significant loss of structural integrity. Furthermore, polyurethanes and epoxy-cured products were synthesized using **B1**. The reaction of **B1** with 4,4′-methylenebis(phenyl isocyanate) (MDI) afforded the corresponding polyurethane (Fig. 5B). The reaction of epichlorohydrin with **B1** afforded epoxide **E1** in a 51% yield (Fig. 5C). Subsequently, two types of epoxy-cured resins were synthesized by adding amine curing agents (Fig. 5D). Therefore, the bisphenol products obtained via the decomposition of silyl ether-linked polymers can be easily recycled to form the original polymers and upcycled to generate various value-added polymers.

## Discussion

In this study, phenylpropanoid-derived decomposable polymers with high biomass contents of 69–79 wt.% were successfully developed. By synthesizing bifunctional monomers linked via silyl ethers and employing cycloaddition polymerization, polymers with unique combinations of aromatic, cyclobutane, and silyl ether groups in their backbones were synthesized. These polymers exhibited excellent thermal properties attributed to their robust structural components. Furthermore, the polymers underwent efficient dual decomposition via Diels-Alder reactions at the cyclobutane rings and Si–O bond cleavage facilitated by fluoride anion, enabling chemical re- and upcycling. This dual decomposability offers a significant advantage in sustainable polymer applications by enabling the decomposition and repurposing of the materials under mild conditions. These findings provide a promising pathway for the development of sustainable polymeric materials using renewable resources, addressing the urgent need to reduce our dependence on petroleum-based polymer materials. This study not only advances the field of biomass-derived polymers but also contributes to environmental sustainability by promoting the circular use of carbon resources. Further increase in molecular weight would be required for practical use, which is currently undergoing in our group. Future research can build on these results to explore further applications and the optimization of these materials.

## Methods

### General procedure for cycloaddition polymerization via chemical oxidation

In a 5 mL vial, a single-electron oxidant dissolved in half the required amount of solvent was added dropwise to the monomer (0.25 mmol) dissolved in the other half of the solvent, while under a nitrogen atmosphere using a balloon. The reaction mixture was stirred for 60 min at a specified temperature and then quenched with methanol. After the solvent was removed under reduced pressure, the crude polymer was purified via reprecipitation (dichloromethane:methanol = 1 mL:20 mL), dissolved in THF at a concentration of 2 mg mL^−1^, and characterized using GPC and NMR.

### General procedure for cycloaddition polymerization via electrochemical oxidation

The reaction was performed in an undivided cell with glassy carbon plates (0.8 × 2 cm) as the anode and cathode, using the ElectraSyn 2.0 Package (IKA, Staufen, Germany). The electrodes were polished using 0.1 and 1 µm alumina and rinsed with deionized water and acetone before the reaction. A solution of the supporting electrolyte was prepared in dichloromethane (0.1 M, 3 mL). After dissolving 0.288 and 0.144 mmol of the monomer and redox mediator, respectively, electrolytic polymerization was performed at room temperature with the application of a constant current. After completion of the reaction, the solvent was removed under reduced pressure. The crude polymer was purified via reprecipitation (dichloromethane:methanol = 1.5 mL:30 mL), dissolved in THF at a concentration of 2 mg mL^−1^, and characterized using GPC and NMR.

### General procedure for cycloaddition polymerization using the photoredox catalyst

In a 5 mL vial, the photocatalyst dissolved in half the required amount of solvent was added dropwise to the monomer (0.25 mmol) dissolved in the other half of the solvent. The reaction mixture was stirred for a defined period under visible or blue light. After the solvent was removed under reduced pressure, the crude polymer was purified via reprecipitation (**P2–P5**, dichloromethane:hexane = 1 mL:20 mL; **P1** and **P6**: dichloromethane:methanol = 1 mL:20 mL), dissolved in THF at a concentration of 2 mg mL^−1^, and characterized using GPC and NMR.

### Procedure for Diels-Alder decomposition

Ru(bpz)_3_[B(C_6_F_5_)_4_]_2_ (2.9 mg, 1 mol.% relative to the cyclobutane rings of **P1**), **P1** (57 mg) dissolved in dichloromethane/1,1,1,3,3,3-hexafluoro-2-propanol (HFIP) (v/v = 1/1, 4.7 mL), and isoprene (0.3 mL) were added to a vial bottle, and the reaction mixture was stirred for 60 h under white light. After completion of the reaction, the dichloromethane and isoprene were removed under reduced pressure. 1,3,5-trimethoxybenzene was then added as an internal standard, and the product was dissolved in deuterated chloroform to calculate the NMR yield of **D1**.

### General procedure for the decomposition at the silyl ether linkage

Two equivalents (relative to the repeating unit of the polymer) of TBAF were added to a solution of the polymer in THF (10 mM repeating units) in the presence of 2 equiv. of acetic acid. The decomposition reaction was conducted by stirring the mixture at room temperature for several hours. After the evaporation of the solvent and vacuum drying, 1 equiv. (relative to the repeating unit of the polymer) of 1,3,5-trimethoxybenzene was added as an internal standard, and the sample was dissolved in the appropriate deuterated solvent. The NMR yields of **B1–B6** were determined using the integral value(s) of their methine groups as an index.

## Supplementary information

Supplementary Information

Transparent Peer Review file

## Source data

Source data

## Data Availability

The experimental and computational data generated in this study are provided in the article and the Supplementary Information, and are also available from the corresponding authors upon request. Source data are provided with this paper.
