# Supplementary material for: Essential oil-derived decomposable polymers via cycloaddition polymerization of silyl ether-linked phenylpropanoids
Source: Nat Commun. 2025 Nov 27;16:10679. doi: 10.1038/s41467-025-65707-x (PMC12660959; doi:10.1038/s41467-025-65707-x)
Supplement: Supplementary file 1 — Supplementary Information [file 41467_2025_65707_MOESM1_ESM.pdf]

Supplementary Information

Essential Oil-Derived Decomposable Polymers via Cycloaddition Polymerization of Silyl Ether-Linked Phenylpropanoids

Ryo Nagaya,[1†] Tatsuya Seko,[1†] Kazuhiro Okamoto,[2] Kazuhide Ueno,[1,3] Mahito Atobe[1,3]*, Naoki Shida[1,3,4]*

[1] Department of Chemistry and Life Science, Yokohama National University, 79-5 Tokiwadai, Hodogaya-ku, Yokohama 240-8501, Japan

[2] Department of Science, University of Toyama, 3190 Gofuku, Toyama, 930-0887, Japan.

[3] Institute of Advanced Sciences, Yokohama National University, 79-5 Tokiwadai, Hodogaya-ku, Yokohama 240-8501, Japan

[4] PRESTO, Japan Science and Technology Agency (JST), 4-1-8 Honcho, Kawaguchi, Saitama 332-0012, Japan

† denotes equal contribution

Table of Contents

1. General Considerations 3

2. Substrate Synthesis 5

2-1. General Procedure A: syntheses of the bifunctional monomers *5*

2-2. General procedures used in polymerization *9*

2-3. General procedures used in the decomposition reactions 10

2-4. General Procedures used in the recycling and upcycling of B1 14

3. Supplementary Data 17

3-1. Optimization of the substituents on the Si atom of the bifunctional monomers 17

3-2. Cyclic voltammetry (CV) 18

3-3. Cycloaddition polymerization of the bifunctional monomers 20

3-4. Thermal properties of the upcycled materials 42

3-5. Decomposition of P1–P6 43

4. Visual Data 48

5. NMR Spectra 49

6. HRMS Data 71

7. Supplementary References 74

1. General Considerations

The chemicals and solvents were purchased from commercial vendors, including Tokyo Chemistry Industry (Tokyo, Japan), Kanto Chemical (Tokyo, Japan), FUJIFILM Wako Pure Chemical (Osaka, Japan), and Sigma-Aldrich (St. Louis, MO, USA), and used without further purification, and an aqueous solution of $NaB(C_6F_5)_4$ was supplied by Nippon Shokubai (Osaka, Japan). $Bu_4NB(C_6F_5)_4$ (Bu = *n*-butyl) was prepared via the salt exchange of Bu_4NBr and $NaB(C_6F_5)_4$.

^{1}H and ^{13}C nuclear magnetic resonance (NMR) spectroscopy was conducted using an ECA-500 (^{1}H: 500 MHz, ^{13}C: 126 MHz; JEOL, Tokyo, Japan). The chemical shifts in $CDCl_3$ are reported in parts per million relative to the signal representing the internal standard tetramethylsilane (0 ppm) and solvent signal at 77.16 ppm in the ^{1}H and ^{13}C NMR spectra, respectively. The chemical shifts in acetone-D_6 are reported in parts per million relative to the solvent signals at 2.05 and 29.84 ppm in the ^{1}H and ^{13}C NMR spectra, respectively. The chemical shifts in dimethyl sulfoxide-D_6 (DMSO-D_6) are reported in parts per million relative to the solvent signals at 2.50 and 39.52 ppm in the ^{1}H and ^{13}C NMR spectra, respectively. The chemical shifts in tetrahydrofuran-D_8 (THF-D_8) are reported in parts per million relative to the solvent signals at 3.58 ppm in the ^{1}H NMR spectra. Chemical shifts in methanol-D_4 are reported in parts per million relative to the solvent signals at 3.31 ppm in the ^{1}H NMR spectra.

High-resolution mass spectrometry (HRMS) was performed using a JMS-T100GCV (JEOL) and microTOF Focus II (Bruker Daltonics, Bruker, Billerica, MA, USA) mass spectrometer.

Analytical gel permeation chromatography (GPC) was performed using a Shodex standard organic solvent size exclusion chromatography GPC column (GPC K-805L, Resonac, Tokyo, Japan) with degassed tetrahydrofuran (THF) as the mobile phase (flow rate: 0.7 mL min^{-1}), and the concentration of the sample within the THF was approximately 2 mg mL^{-1}. Samples were filtered through 0.45 µm polytetrafluoroethylene syringe filters before injection into the instrument. The molar masses were calculated using linear polystyrene calibration standards.

Gas chromatography (GC-FID) analyses were performed using a Shimadzu gas chromatograph (GC2014) equipped with a DB-5ms (Agilent) column. Helium was used as a carrier gas for the GC analyses.

High performance liquid chromatography (HPLC) analyses were performed using a LC pump (LC-20AD, Shimadzu), ultraviolet detector (SPD-20A, Shimadzu), and a reverse phase column (Poroshell 120 EC-C18, 250-4.6 (4 µm), Agilent) under isothermal conditions at 40 °C, with acetonitrile as the mobile phase (flow rate: 1 mL min^{-1}).

Matrix-assisted laser desorption-ionization time-of-flight mass spectrometry (MALDI-TOF-MS) spectra were measured using dithranol as a matrix, and silver(I) trifluoroacetate as an ionizing agent.

Thermogravimetric analysis (TGA) was performed using 5–10 mg of sample and a TG/DTA6200 (Seiko Instruments Inc., Tokyo, Japan) and STA7200RV (Hitachi High-Tech, Tokyo, Japan) under a constant stream of nitrogen gas at a temperature ramp of 30 °C min^{-1} from 30 to 550 °C. T_{d5}, the

temperature at which the sample loses 5% of its mass, was used as an index to evaluate the heat resistance of each sample.

Differential scanning calorimetry (DSC) was performed using a DSC7020 (Hitachi High-Tech) under a constant stream of nitrogen. Each sample (5–10 mg) was sealed in an aluminum pan and subjected to a temperature ramp of 10 °C min^{-1} from –150 to 250 °C. The temperature at the intersection of the tangent line at the inflection point of the DSC thermogram and the tangent line of the baseline is defined as the glass transition temperature (T_g) of the sample.

Computations were performed using Gaussian 16 (Revision C.01) software. Geometry optimizations and were conducted using CAM-B3LYP levels of theory with 6-311+G(d,p) basis set.

2. Substrate Synthesis

2-1. General Procedure A: syntheses of the bifunctional monomers [1]

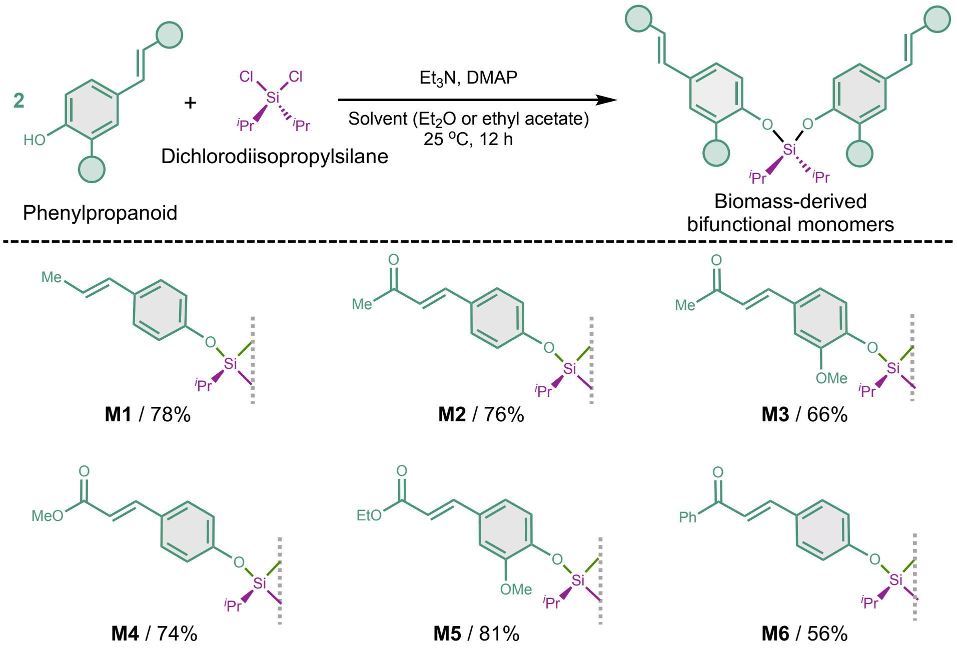

Figure S1. Syntheses of the bifunctional monomers **M1–M6** (Et_2O = diethyl ether).

The phenylpropanoid (2.5–3.0 equiv.) was placed in a two-necked, round-bottom flask equipped with a stirrer bar, followed by the addition of the solvent (dehydrated diethyl ether or ethyl acetate) using a glass syringe under a nitrogen atmosphere. Triethylamine (Et_3N, 2.5–3.0 equiv.), which acted as a proton-withdrawing agent for the phenyl group of the phenylpropanoid, was added. After stirring for several minutes, a catalytic amount (0.1 equiv.) of 4-dimethylaminopyridine (DMAP) dissolved in dehydrated diethyl ether was added using a disposable syringe. DMAP promoted chloride abstraction by coordinating to the silicon atom of the dichlorosilane. Dichlorodiisopropylsilane (1.0 equiv.) was added to the mixture, and the reaction proceeded for 12 h at 25 °C. Subsequently, the ether was removed using rotary evaporation and the crude product was extracted using an organic solvent (CH_2Cl_2 or ethyl acetate) and washed thoroughly with water three times. The solution was dried over Na_2SO_4 and filtered, and the solvent was removed. The product mixture was purified via silica gel column chromatography using an eluent, and the monomer yield was calculated as the percentage of moles of dichlorosilane used.

2-1-1 Synthesis of M1

2-1-1-1. Synthesis of the precursor of M1 [2]

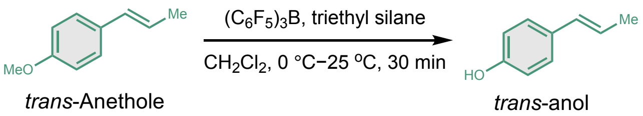

trans-Anethole (6.05 g, 40.8 mmol) was added to a round-bottom flask equipped with a stirrer bar, followed by the addition of dehydrated CH_2Cl_2 (55 mL) and a catalytic amount of tris(pentafluorophenyl)borane (0.123 g, 0.241 mmol). Triethylsilane (7.6 mL, 48 mmol) was added dropwise to the mixture at 0 °C, and it was then stirred at room temperature for 30 min. After the reaction, a tetrabutylammonium fluoride solution (1 M in THF, 42 mL, 42 mmol) was added to remove the silyl protection of the phenols, followed by acidification with aqueous HCl (1 M, 50 mL). The residue was extracted using CH_2Cl_2 and washed with water, and the organic layer was dried over Na_2SO_4. The crude product was purified via silica gel column chromatography (hexane/ethyl acetate, v/v = 4/1), and pure *trans*-anol (5.13 g, 38.2 mmol, 93%) was obtained as a white solid.

^{1}H NMR (500 MHz, CHLOROFORM-*D*) δ 7.23 – 7.19 (m, 2H), 6.79 – 6.72 (m, 2H), 6.37 – 6.29 (m, 1H), 6.08 (dq, J = 15.8, 6.6 Hz, 1H), 4.66 (s, 1H), 1.85 (dd, J = 6.7, 1.7 Hz, 3H). The obtained ^{1}H NMR spectrum corresponded to the reported data.[2]

2-1-1-2. Synthesis of M1

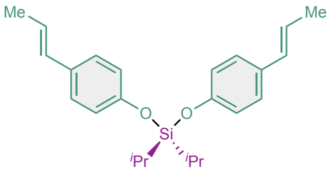

M1

M1 was synthesized according to General Procedure A, using 1.46 g (10.9 mmol) pure *trans*-anol, 25 mL dehydrated diethyl ether, 1.20 g (11.9 mmol) Et_3N, 35.3 mg (0.288 mmol) DMAP, and 0.955 g (5.16 mmol) dichlorodiisopropylsilane. After the reaction, the residue was extracted using CH_2Cl_2, and the product mixture was purified using silica gel column chromatography (hexane/ethyl acetate, v/v = 99/1). Pure **M1** (1.54 g, 4.06 mmol, 78%) was isolated as a viscous, transparent liquid.

^{1}H NMR (500 MHz, CHLOROFORM-*D*) δ 7.22 – 7.15 (m, 4H), 6.92 – 6.86 (m, 4H), 6.36 – 6.29 (m, 2H), 6.09 (dq, J = 15.7, 6.6 Hz, 2H), 1.84 (dd, J = 6.7, 1.7 Hz, 6H), 1.31 – 1.18 (m, 2H), 1.07 (d, J = 7.4 Hz, 12H).

^{13}C NMR (126 MHz, CHLOROFORM-*D*) δ 153.83, 131.88, 130.50, 127.06, 123.99, 119.86, 18.56, 17.24, 12.66.

HRMS (atmospheric pressure chemical ionization (APCI)) calculated for $C_{24}H_{32}O_2Si$ ($[M + H]^+$): 381.2244; measured: 381.2233.

2-1-2. Synthesis of M2

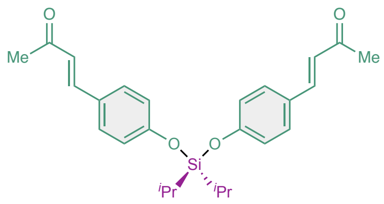

M2

M2 was synthesized according to General Procedure A, using 2.00 g (12.3 mmol) 4-(4-hydroxyphenyl)-3-buten-2-one, 40 mL dehydrated diethyl ether, 1.44 g (14.2 mmol) Et_3N, 65.6 mg (0.536 mmol) DMAP, and 0.896 g (4.84 mmol) dichlorodiisopropylsilane. After the reaction, the residue was extracted using CH_2Cl_2, and the product mixture was purified using silica gel column chromatography (toluene/ethyl acetate, v/v = 17/3). Pure **M2** (1.60 g, 3.68 mmol, 76%) was isolated as a pale yellow solid.

^{1}H NMR (500 MHz, CHLOROFORM-*D*) δ 7.49 – 7.42 (m, 6H), 7.01 – 6.95 (m, 4H), 6.61 (d, *J* = 16.2 Hz, 2H), 2.36 (s, 6H), 1.37 – 1.24 (m, 2H), 1.10 (d, *J* = 7.5 Hz, 12H).

^{13}C NMR (126 MHz, CHLOROFORM-*D*) δ 198.53, 156.88, 143.12, 130.18, 128.48, 125.73, 120.37, 27.59, 17.11, 12.69.

HRMS (APCI) calculated for $C_{26}H_{32}O_4Si$ ($[M + H]^+$): 437.2143; measured: 437.2130.

2-1-3. Synthesis of M3

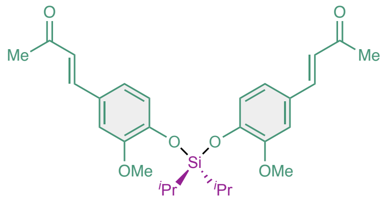

M3

M3 was synthesized according to General Procedure A, using 2.89 g (15.0 mmol) (*E*)-4-(4-hydroxy-3-methoxyphenyl)-3-buten-2-one, 50 mL dehydrated diethyl ether, 1.78 g (17.6 mmol) Et_3N, 79.4 mg (0.649 mmol) DMAP, and 1.14 g (6.18 mmol) dichlorodiisopropylsilane. After the reaction, the residue was extracted using ethyl acetate, and the product mixture was purified using silica gel column chromatography (chloroform/ethyl acetate, v/v = 24/1). Pure **M3** (2.04 g, 4.12 mmol, 66%) was isolated as a pale-yellow viscous liquid or white solid.

^{1}H NMR (500 MHz, CHLOROFORM-*D*) δ 7.45 (d, *J* = 16.2 Hz, 2H), 7.06 – 7.01 (m, 4H), 6.98 (d, *J* = 8.1 Hz, 2H), 6.60 (d, *J* = 16.2 Hz, 2H), 3.78 (s, 6H), 2.37 (s, 6H), 1.36 – 1.24 (m, 2H), 1.09 (d, *J* = 7.4 Hz, 12H).

^{13}C NMR (126 MHz, CHLOROFORM-*D*) δ 198.47, 150.82, 146.85, 143.65, 128.62, 125.57, 122.56, 120.75, 110.82, 55.55, 27.40, 17.06, 13.30.

HRMS (APCI) calculated for $C_{28}H_{36}O_6Si$ ($[M + H]^+$): 497.2354; measured: 497.2346.

2-1-4. Synthesis of M4

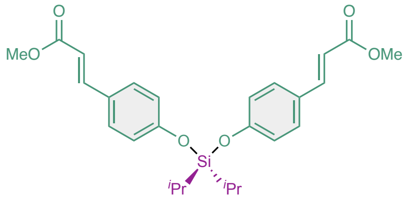

M4

M4 was synthesized according to General Procedure A, using 1.90 g (10.6 mmol) methyl *trans*-*p*-coumarate, 40 mL dehydrated diethyl ether, 1.49 g (14.7 mmol) Et_3N, 119 mg (0.980 mmol) DMAP, and 653 mg (3.53 mmol) dichlorodiisopropylsilane. After the reaction, the residue was extracted using CH_2Cl_2, and the product mixture was purified using silica gel column chromatography (toluene/ethyl acetate, v/v = 17/3). Pure **M4** (1.22 g, 2.61 mmol, 74%) was obtained as a white solid.

^{1}H NMR (500 MHz, CHLOROFORM-*D*) δ 7.63 (d, J = 16.0 Hz, 2H), 7.45 – 7.39 (m, 4H), 7.00 – 6.93 (m, 4H), 6.31 (d, J = 16.0 Hz, 2H), 3.79 (s, 6H), 1.35 – 1.24 (m, 2H), 1.09 (d, J = 7.5 Hz, 12H).

^{13}C NMR (126 MHz, CHLOROFORM-*D*) δ 167.81, 156.70, 144.48, 129.96, 128.53, 120.28, 116.10, 51.77, 17.14, 12.72.

HRMS (electrospray ionization (ESI)) calculated for $C_{26}H_{32}O_6Si$ ($[M + Na]^+$): 491.1860; measured: 491.1852.

2-1-5. Synthesis of M5

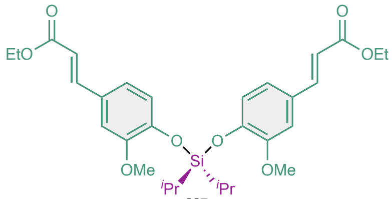

M5

M5 was synthesized according to General Procedure A, using 1.04 g (4.68 mmol) ethyl 4-hydroxy-3-methoxycinnamate, 25 mL dehydrated diethyl ether, 540 mg (5.43 mmol) Et_3N, 31.7 mg (0.259 mmol) DMAP, and 396 mg (2.14 mmol) dichlorodiisopropylsilane. After the reaction, the residue was extracted using CH_2Cl_2, and the product mixture was purified using silica gel column chromatography (hexane:ethyl acetate, v/v = 9/1). Pure **M5** (965 mg, 1.73 mmol, 80%) was obtained as a white solid.

^{1}H NMR (500 MHz, CHLOROFORM-*D*) δ 7.61 (d, J = 15.9 Hz, 2H), 7.03 – 6.98 (m, 4H), 6.96 (d, J = 8.2 Hz, 2H), 6.30 (d, J = 15.9 Hz, 2H), 4.26 (q, J = 7.1 Hz, 4H), 3.76 (s, 6H), 1.33 (t, J = 7.1 Hz, 6H), 1.31 – 1.25 (m, 2H), 1.09 (d, J = 7.4 Hz, 12H).

^{13}C NMR (126 MHz, CHLOROFORM-*D*) δ 167.37, 150.78, 146.62, 144.71, 128.76, 122.22, 120.74, 116.33, 110.85, 60.51, 55.61, 17.15, 14.50, 13.35.

HRMS (ESI) calculated for $C_{30}H_{40}O_8Si$ ($[M + H]^+$): 557.2565, measured: 557.2564.

2-1-6. Synthesis of M6

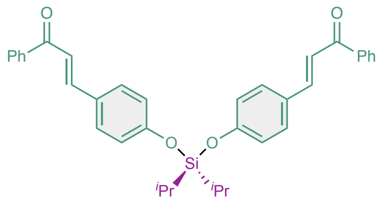

M6

M6 was synthesized according to General Procedure A, using 2.16 g (9.60 mmol) (*E*)-4-hydroxychalcone, 50 mL ethyl acetate, 1.27 g (12.6 mmol) Et_3N, 50.2 mg (0.410 mmol) DMAP, and 789 mg (4.26 mmol) dichlorodiisopropylsilane. After the reaction, the residue was extracted using ethyl acetate, and the product mixture was purified using silica gel column chromatography (hexane/ethyl acetate, v/v = 9/1). Pure **M6** (1.34 g, 2.39 mmol, 56%) was isolated as a pale-yellow viscous liquid.

^{1}H NMR (500 MHz, CHLOROFORM-*D*) δ 8.03 – 7.97 (m, 4H), 7.81 – 7.73 (m, 2H), 7.59 – 7.54 (m, 6H), 7.53 – 7.45 (m, 4H), 7.43 (dd, J = 15.7, 2.2 Hz, 2H), 7.05 – 6.98 (m, 4H), 1.39 – 1.26 (m, 2H), 1.15 – 1.09 (m, 12H).

^{13}C NMR (126 MHz, CHLOROFORM-*D*) δ 190.60, 156.92, 144.51, 138.48, 132.74, 130.41, 129.00, 128.68, 128.53, 120.50, 120.33, 17.11, 12.69.

HRMS (ESI) calculated for $C_{36}H_{36}O_4Si$ ($[M + H]^+$): 561.2456; measured: 561.2459.

2-2. General procedures used in polymerization

2-2-1. General procedure for cycloaddition polymerization via chemical oxidation

In a 5 mL vial, a single-electron oxidant dissolved in half the required amount of solvent was added dropwise to the monomer (0.25 mmol) dissolved in the other half of the solvent, while under a nitrogen atmosphere using a balloon. The reaction mixture was stirred for 60 min at a specified temperature and then quenched with methanol. After the solvent was removed under reduced pressure, the crude polymer was purified via reprecipitation (dichloromethane:methanol = 1 mL:20 mL), dissolved in THF at a concentration of 2 mg mL^{-1}, and characterized using GPC. ^{1}H NMR spectroscopy (500 MHz) was conducted using deuterated chloroform.

2-2-2. General procedure for cycloaddition polymerization via electrochemical oxidation

The reaction was performed in an undivided cell with glassy carbon plates (0.8 × 2 cm) as the anode and cathode, using the ElectraSyn 2.0 Package (IKA, Staufen, Germany). The electrodes were polished using 0.1 and 1 μm alumina and rinsed with deionized water and acetone before the reaction. A solution of the supporting electrolyte was prepared in dichloromethane (0.1 M, 3 mL). After dissolving 0.288 and 0.144 mmol of the monomer and redox mediator, respectively, electrolytic polymerization was performed at room temperature with the application of a constant current. After completion of the

reaction, the solvent was removed under reduced pressure. The crude polymer was purified via reprecipitation (dichloromethane:methanol = 1.5 mL:30 mL), dissolved in THF at a concentration of 2 mg mL^{-1}, and characterized using GPC. ^{1}H NMR spectroscopy (500 MHz) was conducted using deuterated chloroform.

2-2-3. General procedure for cycloaddition polymerization using the photoredox catalyst

In a 5 mL vial, the photocatalyst dissolved in half the required amount of solvent was added dropwise to the monomer (0.25 mmol) dissolved in the other half of the solvent. The reaction mixture was stirred for a defined period under visible or blue light. After the solvent was removed under reduced pressure, the crude polymer was purified via reprecipitation (**P2–P5**, dichloromethane:hexane = 1 mL:20 mL; **P1** and **P6**: dichloromethane:methanol = 1 mL:20 mL), dissolved in THF at a concentration of 2 mg mL^{-1}, and characterized using GPC. ^{1}H NMR spectroscopy (500 MHz) was conducted using deuterated chloroform.

2-3. General procedures used in the decomposition reactions

2-3-1. Diels-Alder decomposition of P1

2-3-1-1. Synthesis of D1

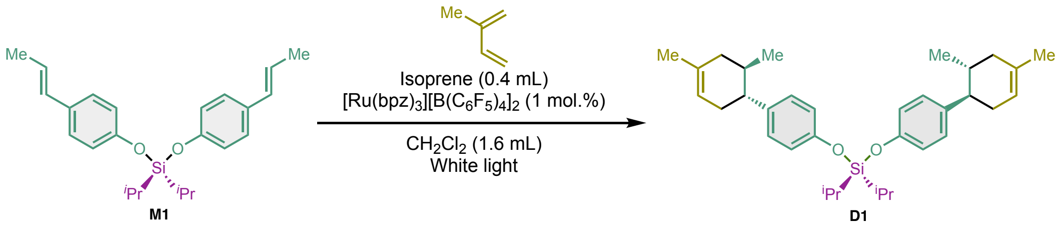

$[Ru(bpz)_3][B(C_6F_5)_4]_2$ (bpz = 2,2′-bipyrazine, 3.9 mg, 1 mol.%), **M1** (76.0 mg, 0.2 mmol) dissolved in dichloromethane (1.6 mL), and isoprene (0.4 mL) were added to a vial bottle, and the reaction mixture was stirred for 48 h under white light. Subsequently, the dichloromethane and isoprene were removed under reduced pressure, and the crude mixture was purified using silica gel column chromatography (hexane/ethyl acetate, v/v = 49/1). Pure **D1** (78.4 mg, 0.15 mmol, 76%) was isolated as a viscous, transparent liquid.

^{1}H NMR (500 MHz, CHLOROFORM-*D*) δ 7.03 – 6.96 (m, 4H), 6.91 – 6.84 (m, 4H), 5.46 – 5.40 (m, 2H), 2.27 (td, J = 10.6, 5.2 Hz, 2H), 2.23 – 2.09 (m, 4H), 2.09 – 2.01 (m, 2H), 1.91 – 1.73 (m, 4H), 1.68 (s, 6H), 1.32 – 1.20 (m, 2H), 1.07 (d, J = 7.5 Hz, 12H), 0.68 (d, J = 6.2 Hz, 6H).

^{13}C NMR (126 MHz, CHLOROFORM-*D*) δ 152.96, 139.34, 133.92, 128.64, 121.08, 119.71, 47.17, 39.99, 35.25, 34.18, 23.52, 20.35, 17.26, 12.70.

HRMS (ESI) calculated for $C_{34}H_{48}O_2Si$ ($[M + H]^+$): 517.3496; measured: 517.3497.

2-3-1-2. General procedure used in Diels-Alder decomposition

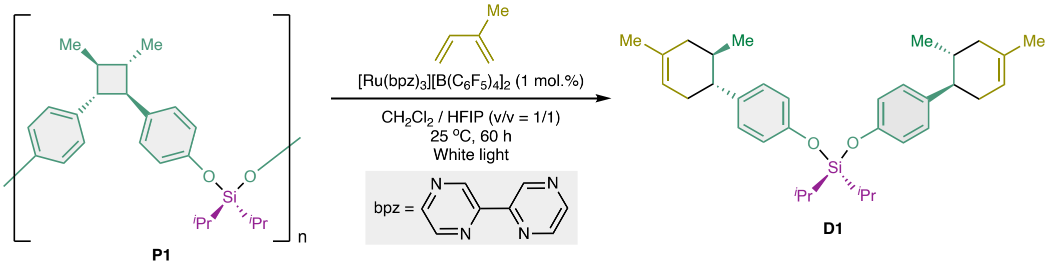

$Ru(bpz)_3[B(C_6F_5)_4]_2$ (2.9 mg, 1 mol.% relative to the cyclobutane rings of **P1**), **P1** (57 mg) dissolved in dichloromethane/1,1,1,3,3,3-hexafluoro-2-propanol (HFIP) (v/v = 1/1, 4.7 mL), and isoprene (0.3 mL) were added to a vial bottle, and the reaction mixture was stirred for 60 h under white light. After completion of the reaction, the dichloromethane and isoprene were removed under reduced pressure. 1,3,5-trimethoxybenzene was then added as an internal standard, and the product was dissolved in deuterated chloroform to calculate the NMR yield of **D1**.

2-3-2. Decomposition at the linker moieties

2-3-2-1. General procedure B: decomposition at the linker moieties

Two equivalents (relative to the repeating unit of the polymer) of TBAF were added to a solution of the polymer in THF (10 mM repeating units) in the presence of 2 equiv. of acetic acid. The decomposition reaction was conducted by stirring the mixture at room temperature for several hours. After the evaporation of the solvent and vacuum drying, 1 equiv. (relative to the repeating unit of the polymer) of 1,3,5-trimethoxybenzene was added as an internal standard, and the sample was dissolved in the appropriate deuterated solvent. The NMR yields of **B1–B6** were determined using the integral value(s) of their methine groups as an index.

2-3-2-1-1. Decomposition of P1 at linker moiety

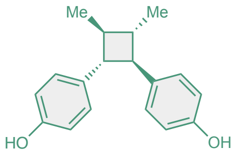

B1

B1 was obtained according to General Procedure B using 72.1 mg **P1**. The NMR yield of **B1** was determined to be 62% using acetone-D_6.

^{1}H NMR (500 MHz, ACETONE-D_6) δ 8.10 (s, 2H), 7.08 – 7.05 (m, 4H), 6.76 – 6.72 (m, 4H), 2.76 – 2.74 (m, 2H), 1.81 – 1.72 (m, 2H), 1.16 – 1.14 (m, 6H).

^{13}C NMR (126 MHz, ACETONE-D_6) δ 156.61, 135.35, 128.66, 115.90, 53.72, 44.16, 18.90.

HRMS (ESI) calculated for $C_{18}H_{20}O_2$ ($[M - H]^+$): 267.1391, measured: 267.1400.

2-3-2-1-2. Decomposition of P2 at linker moiety

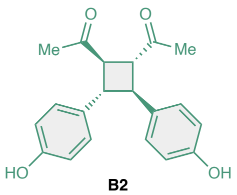

B2

B2 was obtained according to General Procedure B using 100.7 mg **P2**. The NMR yield of **B2** was determined to be 58% using THF-D_8.

^{1}H NMR (500 MHz, CHLOROFORM-*D*) δ 7.15 – 7.07 (m, 4H), 6.84 – 6.76 (m, 4H), 5.38 (s, 2H), 3.63 – 3.45 (m, 2H), 3.39 – 3.11 (m, 2H), 2.07 (s, 6H).

^{13}C NMR (126 MHz, ACETONE-D_6) δ 206.89, 157.35, 133.42, 129.15, 116.23, 52.33, 48.23, 28.45.

HRMS (APCI) calculated for $C_{20}H_{20}O_4$ ([M + H]$^+$): 325.1434, measured: 325.1434.

2-3-2-1-3. Decomposition of P3 at linker moiety

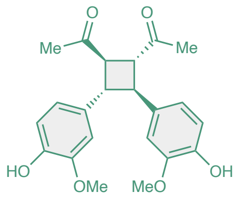

B3

B3 was obtained according to General Procedure B using 99.1 mg **P3**. The NMR yield of **B3** was determined to be 59% using acetone-D_6.

^{1}H NMR (500 MHz, CHLOROFORM-*D*) δ 6.86 (d, J = 8.2 Hz, 2H), 6.76 (dd, J = 8.1, 2.0 Hz, 2H), 6.72 (d, J = 2.0 Hz, 2H), 5.73 (s, 2H), 3.85 (s, 6H), 3.57 – 3.51 (m, 2H), 3.38 – 3.27 (m, 2H), 2.08 (s, 6H).

^{13}C NMR (126 MHz, ACETONE-D_6) δ 206.94, 148.42, 146.57, 134.07, 120.67, 115.85, 111.54, 56.24, 52.26, 48.65, 28.49.

HRMS (APCI) calculated for $C_{22}H_{24}O_6$ ([M + Cl]$^-$): 419.1267, measured: 419.1271.

2-3-2-1-4. Decomposition of P4 at linker moiety

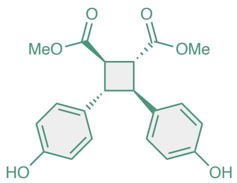

B4

B4 was obtained according to General Procedure B using 95.2 mg **P4**. The NMR yield of **B4** was determined to be 62% using THF-D_8.

^{1}H NMR (500 MHz, ACETONE-D_6) δ 7.19 – 7.13 (m, 4H), 6.82 – 6.77 (m, 4H), 3.68 (s, 6H), 3.56 – 3.51 (m, 2H), 3.37 – 3.32 (m, 2H).

^{13}C NMR (126 MHz, ACETONE-D_6) δ 173.46, 157.39, 133.07, 128.88, 116.19, 52.18, 48.47, 45.61.

HRMS (APCI) calculated for $C_{20}H_{20}O_6$ ($[M + H]^+$): 357.1333, measured: 357.1325.

2-3-2-1-5. Decomposition of P5 at linker moiety

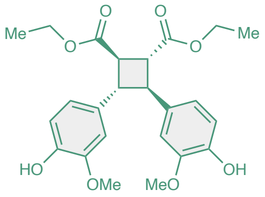

B5

B5 was obtained according to General Procedure B using 93.4 mg **P5**. The NMR yield of **B5** was determined to be 99% using acetone-D_6.

^{1}H NMR (500 MHz, ACETONE-D_6) δ 6.97 (d, J = 1.8 Hz, 2H), 6.83 – 6.77 (m, 4H), 4.16 (q, J = 6.5 Hz, 4H), 3.83 (s, 6H), 3.62 – 3.55 (m, 2H), 3.35 – 3.30 (m, 2H), 2.87 (s, 2H), 1.23 (t, J = 7.1 Hz, 6H).

^{13}C NMR (126 MHz, ACETONE-D_6) δ 173.06, 148.36, 146.57, 133.94, 120.26, 115.82, 111.33, 61.25, 56.21, 48.38, 45.88, 14.56.

HRMS (APCI) calculated for $C_{24}H_{28}O_8$ ($[M + Na]^+$): 467.1676, measured: 467.1675.

2-3-2-1-6. Decomposition of P6 at linker moiety

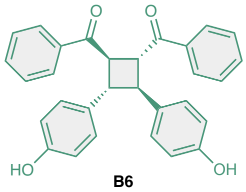

B6

B6 was obtained according to General Procedure B using 107.3 mg **P6**. The NMR yield of **B6** was determined to be 50% using methanol-D_4.

^{1}H NMR (500 MHz, ACETONE-D_6) δ 8.37 – 8.24 (m, 2H), 7.89 – 7.82 (m, 4H), 7.57 – 7.49 (m, 2H), 7.41 – 7.34 (m, 4H), 7.24 – 7.16 (m, 4H), 6.83 – 6.77 (m, 4H), 4.58 – 4.54 (m, 2H), 3.78 – 3.73 (m, 2H).

^{13}C NMR (126 MHz, ACETONE-D_6) δ 199.49, 157.47, 136.88, 134.16, 133.41, 129.49, 129.43, 129.37, 116.29, 49.24, 48.66.

HRMS (APCI) calculated for $C_{30}H_{24}O_4$ ($[M + H]^+$): 449.1747, measured: 449.1746.

2-4. General Procedures used in the recycling and upcycling of B1

2-4-1. Recycling of B1

2-4-1-1. General procedure used in the recycling of B1

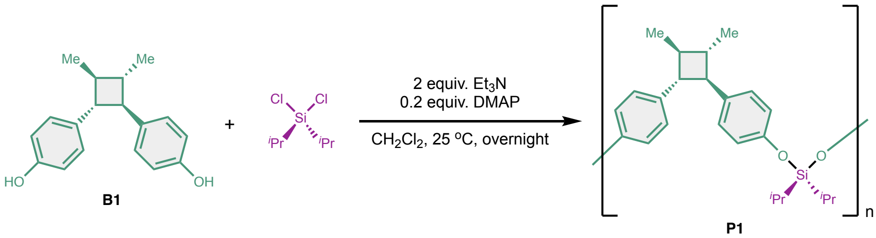

B1 (67.1 mg, 0.25 mmol) was added to a two-necked round-bottom flask equipped with a stirrer bar, followed by dehydration in dichloromethane (0.2 mL) under a nitrogen atmosphere. Et_3N (50.6 mg, 0.5 mmol) was added, and the mixture was stirred for several minutes to facilitate the deprotonation of the phenols. Subsequently, DMAP (6.1 mg, 0.05 mmol) dissolved in dehydrated acetonitrile (0.8 mL) was added, and the reaction was initiated via the dropwise addition of dichlorodiisopropylsilane (46.3 mg, 0.25 mmol) at room temperature, followed by stirring for 12 h. After completion of the reaction, the mixture was dissolved in dichloromethane (0.5 mL) and dispersed in methanol (20 mL). The resulting precipitate was filtered, and the residue was recovered using THF. **P1** (91 mg, 96%) was obtained after the evaporation of the THF. GPC revealed that **P1** displayed a number- (M_n) and weight-average molecular weight (M_w) and polydispersity index (PDI) of 2900, 6700, and 2.35, respectively.

2-4-2. Upcycling of B1

2-4-2-1. Synthesis of epoxy cured resins 1 and 2

2-4-2-1-1. Synthesis of E1

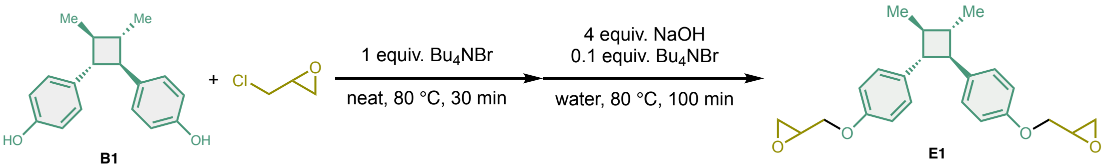

B1 (0.642 g, 2.39 mmol) was added to a two-necked round-bottom flask equipped with a stirrer bar, followed by the addition of tetrabutylammonium bromide (TBAB, 0.771 g, 2.39 mmol) under a nitrogen atmosphere. Epichlorohydrin (1.88 mL, 23.9 mmol) was then added, and the mixture was stirred for 30 min at 80 °C. Thereafter, 1.91 mL of aqueous sodium hydroxide (5 M) and TBAB (0.0771 g, 0.239 mmol) were added to the mixture, which was then stirred for 100 min at 80 °C. Subsequently, the reaction mixture was extracted with ethyl acetate and washed three times with water. The solution was dried over Na_2SO_4 and filtered, the solvent was removed, and the product mixture was purified using silica gel column chromatography (hexane/ethyl acetate, v/v = 3/1). Pure **E1** (460 mg, 1.21 mmol, 51%) was isolated as a transparent viscous liquid.

^{1}H NMR (500 MHz, ACETONE-D_6) δ 7.20 – 7.14 (m, 4H), 6.90 – 6.85 (m, 4H), 4.27 (dd, J = 11.2, 2.8 Hz, 2H), 3.84 (dd, J = 11.3, 6.3 Hz, 2H), 3.31 – 3.25 (m, 2H), 2.82 – 2.79 (m, 4H), 2.68 (dd, J = 5.2, 2.6 Hz, 2H), 1.85 – 1.77 (m, 2H), 1.21 – 1.14 (m, 6H).

^{13}C NMR (126 MHz, CHLOROFORM-D) δ 156.98, 136.56, 127.86, 114.59, 68.90, 52.53, 50.27, 44.80, 43.28, 18.94.

HRMS (ESI) calculated for $C_{24}H_{28}O_4$ ($[M + Na]^+$): 403.1880; measured: 403.1874.

2-4-2-1-2. Synthesis of epoxy cured resin 1

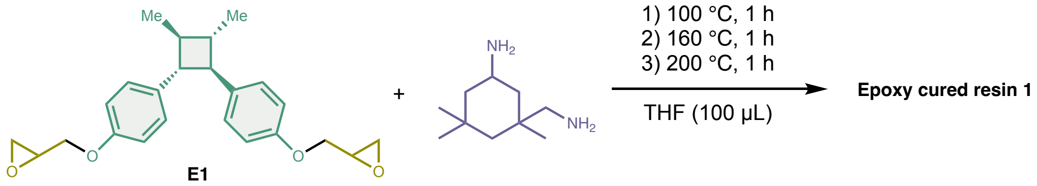

E1 (0.080 g, 0.21 mmol), THF (100 μL), and isophoronediamine (19.3 μL, 0.105 mmol) were added to a vial bottle, and the mixture was fully dispersed via ultrasonication. The mixture (20 μL) was transferred to an aluminum pan for use in TGA (or DSC). The reaction mixture was heated in the oven for 1 h at 100 °C, then for 1 h at 160 °C, and then for 1 h at 200 °C. After cooling to room temperature, the epoxy cured resin was directly used in TGA (or DSC). Its respective $T_{d5\%}$ and T_g were 336.6 and 52.0 °C (Figure S18).

2-4-2-1-3. Synthesis of epoxy cured resin 2

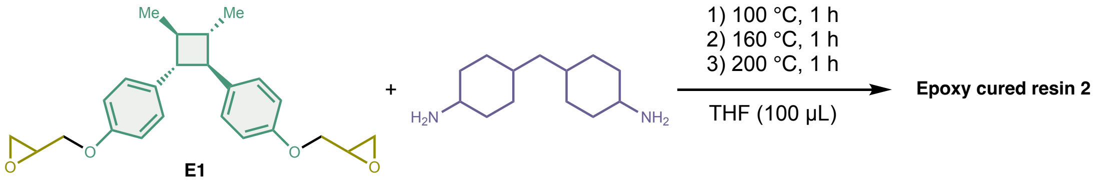

E1 (0.0802 g, 0.211 mmol), THF (100 μL), and 4,4′-methylenebis(cyclohexylamine) (21.9 mg, 0.104 mmol) were added to a vial bottle, and the mixture was fully dispersed via ultrasonication. The mixture (20 μL) was transferred to an aluminum pan for use in TGA (or DSC). The reaction mixture was heated in the oven for 1 h at 100 °C, then for 1 h at 160 °C, and then for 1 h at 200 °C. After cooling to room temperature, the epoxy cured resin was directly used in TGA (or DSC). Its respective $T_{d5\%}$ and T_g were 338.8 and 60.5 °C (Figure S18).

2-4-2-2. Synthesis of polyurethane

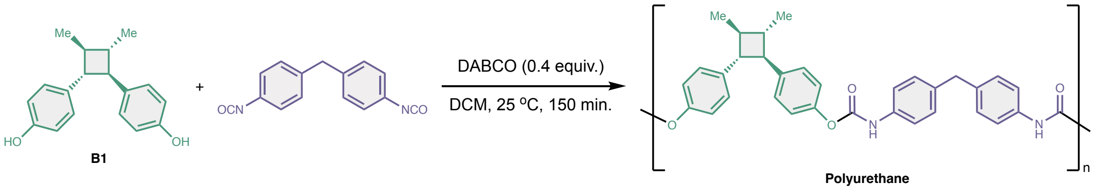

B1 (0.1008 g, 0.376 mmol) and 1,4-diazabicyclo[2.2.2]octane (DABCO, 0.0167 g, 0.150 mmol) were dissolved in dichloromethane (0.4 mL) in a vial bottle. 4,4′-Methylenebis(phenyl isocyanate) (MDI, 93.2 mg, 0.372 mmol) in dichloromethane (0.6 mL) was then poured into the mixture, which was then stirred for 2.5 h at room temperature. Subsequently, the mixture was dispersed in methanol (20 mL), and the precipitate was filtered, the residue was dried, and polyurethane (139.4 mg, 72%) was obtained as a pale-yellow solid. GPC revealed that the polyurethane exhibited M_n = 3100, M_w = 10000, and PDI = 3.27. Its $T_{d5\%}$ was 253.4 °C (Figure S19).

^{1}H NMR (500 MHz, DMSO-D_6) δ 7.03 – 6.95 (m, 4H), 6.80 (d, J = 8.3 Hz, 4H), 6.70 – 6.62 (m, 4H), 6.47 – 6.42 (m, 4H), 4.79 (s, 4H), 2.71 – 2.65 (m, 2H), 1.72 – 1.63 (m, 2H), 1.13 – 1.04 (m, 6H).

3. Supplementary Data

3-1. Optimization of the substituents on the Si atom of the bifunctional monomers

Table S1. Influences of the **R** substituents on the yield of the bifunctional monomer

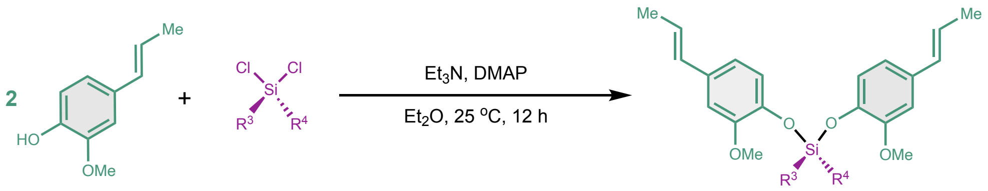

Entry	Substituent on the Si atom - R^3	- R^4	Yield of monomer [%]
1	- Me	- Me	Unstable
2	- Et	- Et	80
3	- ^{i}Pr	- ^{i}Pr	93
4	- ^{t}Bu	- ^{t}Bu	N.D.
5	- Ph	- Me	Unstable
6	- Ph	- Ph	N.D.

Substituent effects on the silicon atoms in bifunctional monomers were examined using isoeugenol. Unhindered substituents such as methyl group resulted in the decompositions of the resulting monomers under ambient conditions, while more sterically hindered groups such as ^{t}Bu and phenyl did not react with isoeugenol. Monomers with ethyl and isopropyl groups were found to form stable structures.

3-2. Cyclic voltammetry (CV)

3-2-1. General procedure used in CV

A glassy carbon disk (φ = 3 mm), platinum plate (2 × 2 cm), and Ag/$AgNO_3$ were used as the working, counter, and reference electrodes, respectively. The working electrode was polished using 0.1 and 1 μm alumina and rinsed with deionized water and acetone before use. After measuring the background of 0.1 M tetrabutylammonium hexafluorophosphate as the supporting electrolyte in 5 mL of CH_2Cl_2, 10 mM of the substrate (**M1–M6** or the mediator) was added and measurements were performed at a scan rate of 0.1 V s^{-1}.

3-2-2. CV of the substrates

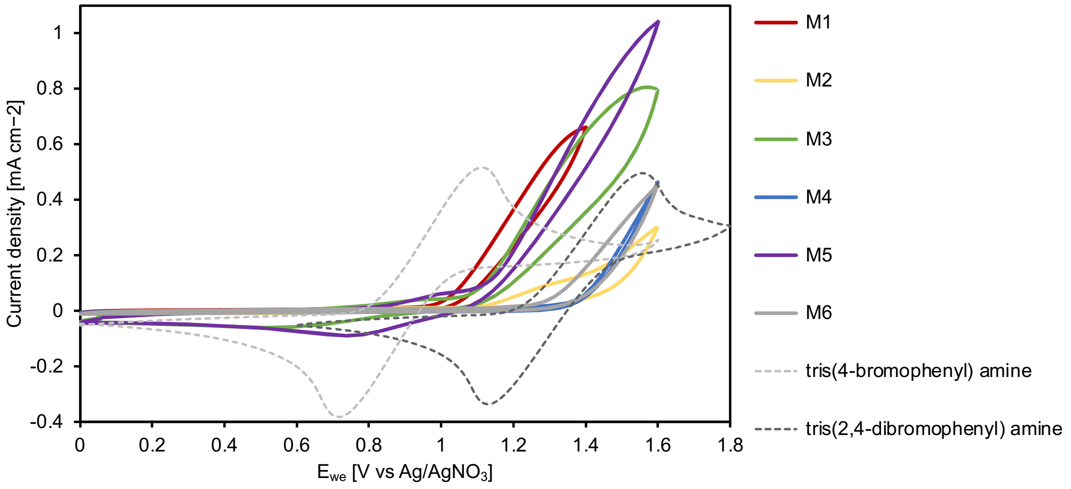

Figure S2. Cyclic voltammograms of **M1–M6**, tris(4-bromophenyl) amine, and tris(2,4-dibromophenyl) amine.

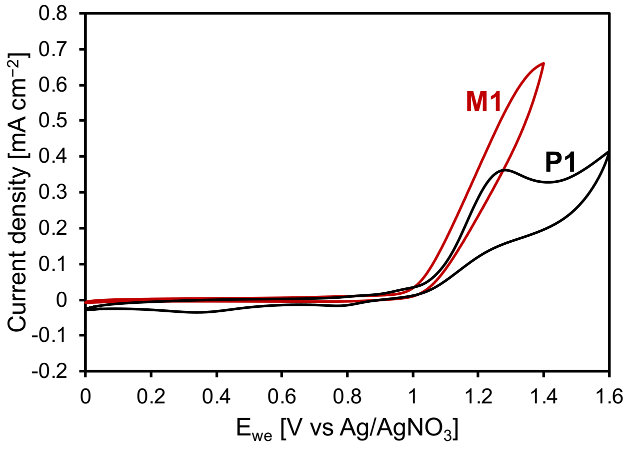

Figure S3. Cyclic voltammograms of **M1** and **P1**.

Table S2. HOMO energies of **M1-M6** identified from DFT calculation

Entry	Monomer	Energy of HOMO [eV]
**1**	**M1**	-7.15
**2**	**M2**	-7.74
**3**	**M3**	-7.41
**4**	**M4**	-7.82
**5**	**M5**	-7.31
**6**	**M6**	-7.70

The estimations of HOMO energies of **M1-M6** were conducted using Gaussian 16 (Revision C.01). Geometry optimizations and were calculated using CAM-B3LYP levels of theory with 6-311+G(d,p) basis set. The order of HOMO energy was generally consistent with the CV results—where **M1** exhibited the highest value—suggesting that **M1** is the most susceptible to oxidation.

3-3. Cycloaddition polymerization of the bifunctional monomers

3-3-1. [2 + 2] cycloaddition polymerization of M1 using a chemical oxidant

3-3-1-1. Optimization of the reaction conditions

Magic blue (MB) and iodobenzene diacetate (PIDA) were used as the chemical oxidants to initiate the polymerization of **M1**. However, when using MB as the initiator, the proportion of the desired structure in the main chain of the resulting polymer is relatively low, i.e., yield of **B1** by the Si-O bond cleavage of **P1** is only 20–48%. Therefore, the progress of another polymerization, in parallel with [2 + 2] cycloaddition polymerization, results in lower yields. According to Bauld et al., cationic polymerization proceeds due to Brønsted acid generation as a side reaction in the [2 + 2] cycloaddition polymerization of bifunctional olefin monomers initiated by MB, which leads to the formation of acyclic structures.[3] A similar phenomenon may be induced during the polymerization of **M1** using MB, forming undesirable cross-linked structures (Table S3). In the GPC trace of the decomposed mixture of the polymer synthesized using MB in CH_2Cl_2 (Table S3, Entry 2) after treatment with tetrabutylammonium fluoride (TBAF), peaks representing residual segments are observed at earlier retention times than that of **B1** (Figure S4A). Furthermore, gelatinous products that do not dissolve in any solvent are produced (Figure S5), and they are scarcely decomposed via TBAF treatment. The acyclic segments formed via cationic polymerization may not contain siloxane bonds, and thus, the backbone of the resulting polymer is not cleaved by the fluoride anions and remains intact.

In contrast, the polymer synthesized using PIDA in CH_2Cl_2/HFIP is soluble in the reaction solution and completely decomposes into **B1** (Figure S4B). The use of PIDA as an initiator instead of MB, which may generate acid, can inhibit cationic polymerization. Additionally, the solvent effect of HFIP may stabilize the catalytic radical cations, thus inhibiting the abstraction of protons and promoting the formation of cyclobutane rings via oxidative [2 + 2] cycloaddition.[4]

Table S3. Polymerization of **M1** using Magic Blue

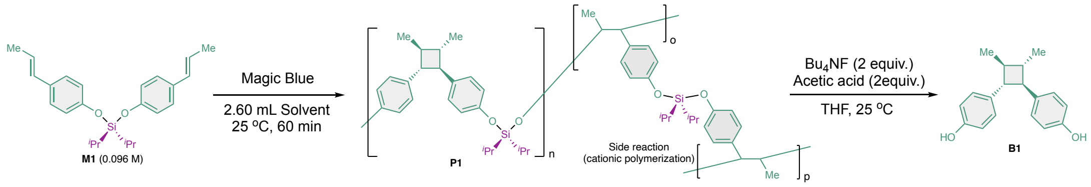

Entry	Amount of MB [mol.%]	Solvent	Yield of P1 [%]	M_n	PDI	Yield of B1 [%]
**1**	5	CH_2Cl_2	91	5000	2.85	29
**2**	10	CH_2Cl_2	83	5800	4.44	20
**3**	10	CH_2Cl_2:HFIP = 1:1	Unmeasurable	-	-	-
**4**	10	HFIP	41	5500	3.28	48

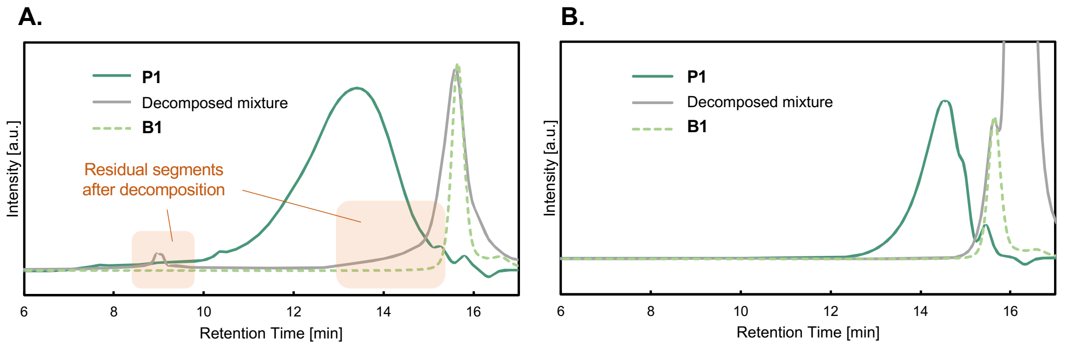

Figure S4. GPC traces (in THF) before/after the decomposition of 10 mM **P1** synthesized using (**A**) magic blue (Table S3, Entry 2) or (**B**) PIDA (Table S4, Entry 1).

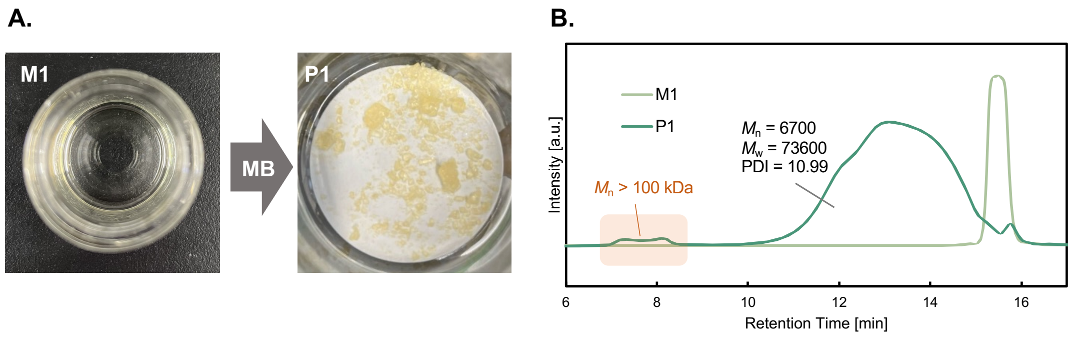

Figure S5. Polymerization of 0.192 M **M1** initiated by 10 mol.% MB in CH_2Cl_2 for 60 min at 25 °C. (**A**) Gelatinous products obtained via the polymerization of **M1**. (**B**) GPC traces before/after the polymerization of **M1**.

Table S4. Hole-catalytic polymerization of **M1** at various concentrations

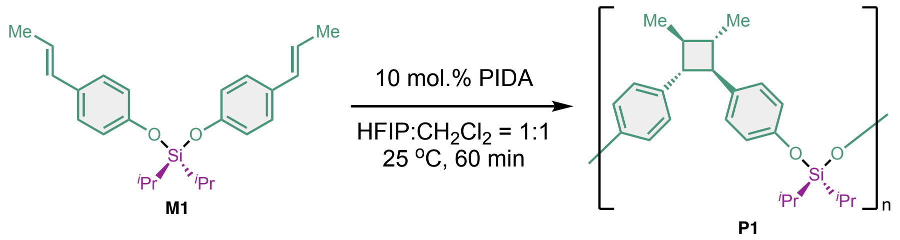

Entry	Concentration of M1 [M]	Polymer Yield [%]	M_n	M_w	PDI
1	0.096	69	1800	3100	1.69
2	0.192	79	2400	4600	1.94
3	0.384	80	2300	4400	1.85
4	0.768	67	2000	3500	1.69

Table S5. The effect of adding extra PIDA and/or **M1** on molecular weight of **P1**

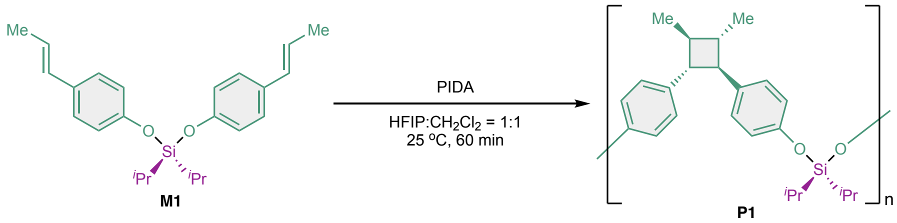

Entry	Initial condition		Variation from the initial condition		Yield of P1 [%]	M_n	M_w	PDI
	M1 [mmol]	PIDA [mol.%]	M1 [mmol]	PIDA [mol.%]				
**1**	0.25	10	0.25	10	79	2400	4600	1.94
**2** [a]	0.19	10	0.19 → 0.40	10 → 5	— [c]	1400	3500	2.57
**3** [b]	0.25	3.3	0.25	3.3 → 10	81	1900	4600	2.47
**4** [a]	0.19	3.3	0.19 → 0.55	3.3	— [c]	1700	3900	2.36

M1 and/or PIDA were added in three portions at a) 1 min, b) 10 min intervals. c) It was not possible to determine the exact yields due to the large amount of monomer remaining.

Although we performed polymerization by adding extra **M1** during the reaction, the molecular weight of **P1** did not increase (TableS5, Entry 2). However, when we added extra PIDA during the reaction (TableS5, Entry 3), the molecular weight increased compared to when less than 10 mol.% of PIDA was added (TableS6 Entries 1-3). Furthermore, when we added extra **M1** together with PIDA during the reaction (TableS5 Entry 4), the molecular weight did not improve. These results suggested that the molecular weight of **P1** greatly depends on the equivalent amount of PIDA rather than the concentration of **M1**.

Table S6. The effect of PIDA equivalent on hole-catalytic polymerization using HFIP/CH_2Cl_2 co-solvent

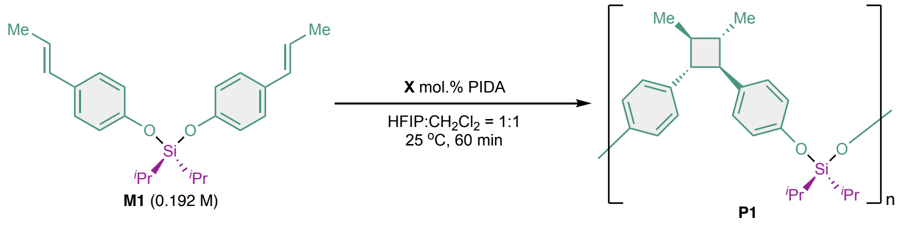

Entry	Amount of PIDA [mol.%]	Yield of P1 [%]	M_n	M_w	PDI
1	1.25	41	1200	1900	1.58
2	2.5	46	1500	2800	1.84
3	5	60	1800	3900	2.22
4	3.3 × 3 [a]	81	1900	4600	2.47
5	10	79	2400	4600	1.94
6	30	77	2200	5100	2.30
7	50	61	2200	5200	2.40
8	100	14	2000	3800	1.91

a) PIDA was added in three portions at 10 min intervals.

Table S7. The effect of PIDA equivalent on hole-catalytic polymerization using HFIP

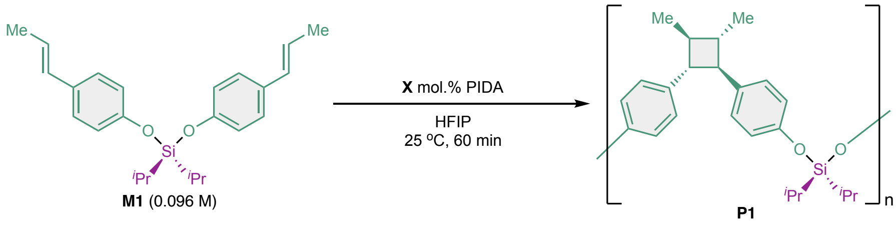

Entry	Amount of PIDA [mol.%]	Yield of P1 [%]	M_n	M_w	PDI
1	10	75	2400	3700	1.57
2	30	76	2800	5000	1.76
3	50	54	3300	6200	1.90
4	100	25	3800	13600	3.60

Table S8. Hole-catalytic polymerization of **M1** using a photocatalyst

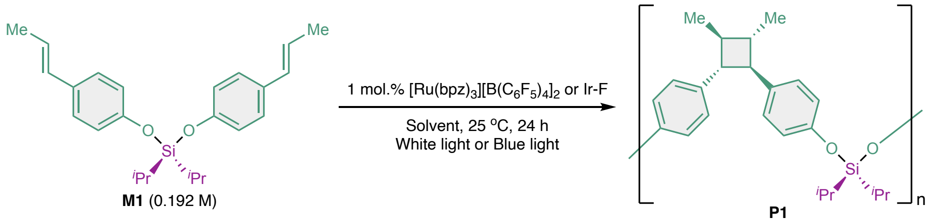

Entry	Photocatalyst	Solvent	Light	Yield [%]	M_n	PDI
**1**	Ir-F	MeCN	Blue	N.D.	-	-
**2**	$[Ru(bpz)_3][B(C_6F_5)_4]_2$	CH_2Cl_2/HFIP (v/v = 1/1)	White	40	2500	3.51
**3**	$[Ru(bpz)_3][B(C_6F_5)_4]_2$	HFIP	White	56	3600	4.06

Oxidizing powers of Ir-F and $[Ru(bpz)_3][B(C_6F_5)_4]_2$ are 1.21 and 1.45 V *vs.* SCE, respectively.[5]

3-3-1-2. Investigation of the mechanism of hole-catalytic polymerization of M1

3-3-1-2-1. General procedure used in investigating the polymerization mechanism

(i) Calibration curve obtained using GC analyses

Standard solutions were prepared by dissolving specific amounts of **M1** (2, 2.5, 4, and 5 mM) and dodecane (10 mM) as the internal standard in CH_2Cl_2. The peak intensities of **M1** and dodecane in the gas chromatogram of each standard solution were analyzed to construct a calibration curve that revealed the relationship between the ratios of the peak intensities and concentrations of **M1**/dodecane. The calibration curve indicates that (peak intensity ratio) = 1.4564 × (concentration ratio) and $R^2 = 0.9996$ (Table S9, Figure S6).

Table S9. Data used in constructing the calibration curve of the polymerization behavior of **M1**

Concentration of **M1** [M]	Concentration of dodecane [M]	Concentration ratio	Peak intensity of **M1**	Peak intensity of dodecane	Peak intensity ratio
0.005	0.01	0.5	128204	174702	0.733843917
0.004	0.01	0.4	103699	179139	0.578874505
0.0025	0.01	0.25	64057	183504	0.349076859
0.002	0.01	0.2	50107	165149	0.303404804

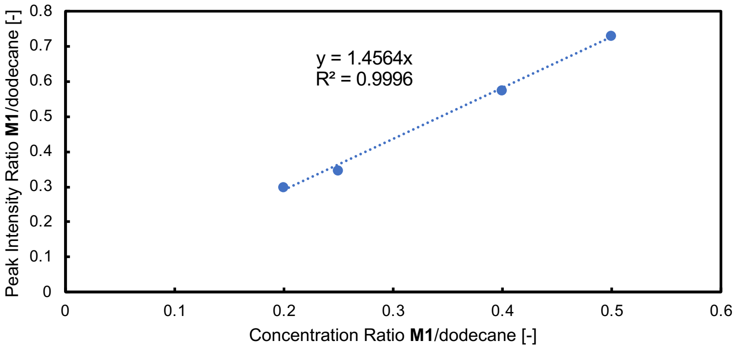

Figure S6. Calibration curve of the polymerization behavior of **M1**

(ii) Investigation of the polymerization behavior

M1 (95.1 mg, 0.25 mmol) and dodecane (113 μL, 0.50 mmol) as an internal standard were added to 1.5 mL dichloromethane in a 5 mL vial. The reaction was initiated via the external injection of the oxidant dissolved in dichloromethane (1.0 mL) at room temperature under a nitrogen atmosphere. The initial concentration of **M1** was 0.1 M, and the concentration of dodecane was fixed at 0.2 M. At predetermined times (0.5, 1, 2, 5, 10, 30, and 60 min) after injecting the oxidant, 0.05 mL of the reaction solution was sampled and diluted with 0.95 mL of THF. The conversion of **M1** in each diluted solution was determined using GC, and the M_n, M_w, and PDI were determined using GPC.

3-3-1-2-2. Polymerization behavior of M1 initiated by 10 mol.% PIDA in CH_2Cl_2/HFIP

A mechanistic study of the polymerization mechanism was also conducted. The reaction was initiated by adding 10 mol.% PIDA to 0.1 M **M1** in CH_2Cl_2/HFIP at room temperature under a nitrogen atmosphere. Sampling was performed at predetermined times (0.5, 1, 2, 5, 10, 30, and 60 min) after injecting PIDA, the conversion of **M1** was determined using GC, and the molecular weight of **P1** was established via GPC.

Table S10. Polymerization behavior of **M1**, as determined using GC and GPC

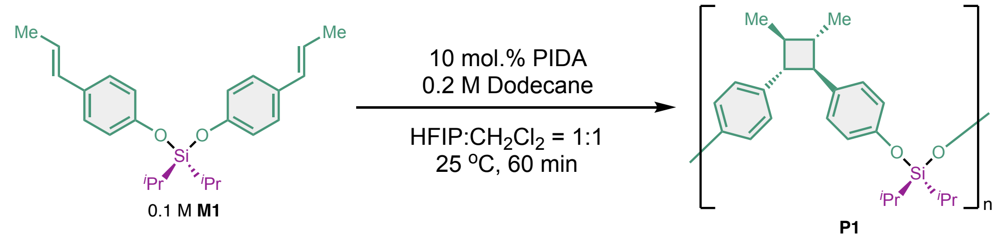

min	Concentration of **M1** [M]	Concentration of dodecane [M]	Concentration ratio	Peak intensity of **M1**	Peak intensity of dodecane	Peak intensity ratio	Conversion of **M1**	M_n (Experimental)	M_w (Experimental)	PDI	M_n (Theoretical)	M_w (Theoretical)
0.5	0.003378529	0.01	0.337852933	78749	160043	0.492049012	32.42941337	994	1180	1.18	563	746
1	0.001891894	0.01	0.189189397	44680	162157	0.275535438	62.16212059	1180	1440	1.22	1006	1631
2	0.00117824	0.01	0.11782404	37528	218696	0.171598932	76.435192	1230	1669	1.38	1615	2850
5	0.000665503	0.01	0.066550289	14040	144856	0.096923842	86.6899421	1305	1940	1.48	2859	5338
10	0.000466739	0.01	0.046673917	11516	169413	0.067975893	90.66521653	1396	2207	1.58	4077	7774
30	0.000390687	0.01	0.039068716	9005	158261	0.056899678	92.18625675	1424	2279	1.6	4871	9361
60	0.000280582	0.01	0.028058192	7127	174408	0.040863951	94.38836155	1439	2360	1.64	6782	13184

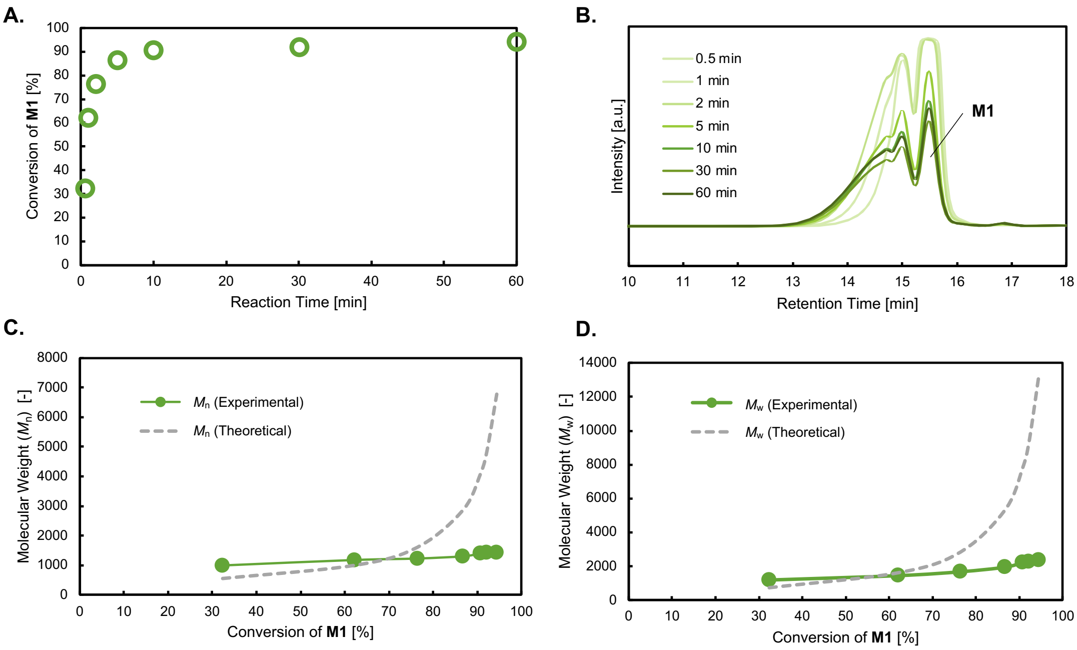

Figure S7. Time courses of the (**A**) conversion of **M1** and (**B**) GPC trace (in THF). Relationships between the conversion of **M1** and (**C**) number- (M_n) and (**D**) weight-average molecular weight (M_w). The green and dotted gray lines respectively represent the experimental data and theoretical results of stepwise polymerization.

3-3-1-2-3. Polymerization behavior of M1 initiated by 5 mol.% MB in CH_2Cl_2

The reaction was initiated by adding 5 mol.% MB to 0.1 M **M1** in CH_2Cl_2 at room temperature under a nitrogen atmosphere. Sampling was performed at predetermined times (0.5, 1, 2, 5, 10, 30, and 60 min) after injecting MB, the conversion of **M1** was determined using GC, and the molecular weight of **P1** was established via GPC. As a result, it showed a great increase of M_n and M_w in the region where the conversion ratio of **M1** is high. These data strongly suggest that the reaction proceeds via a stepwise mechanism.

Table S11. Polymerization behavior of **M1**, as determined using GC and GPC

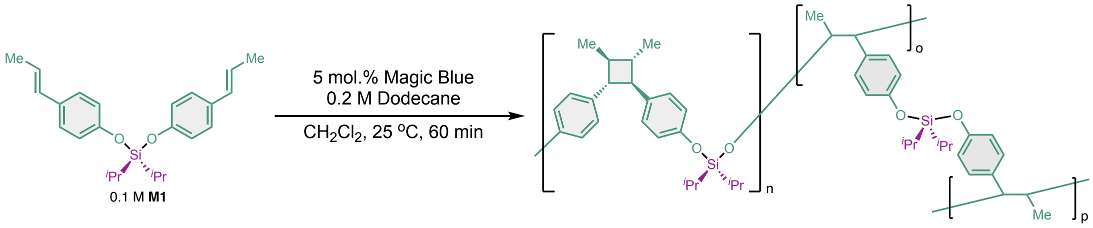

min	Concentration of **M1** [M]	Concentration of dodecane [M]	Concentration ratio	Peak intensity of **M1**	Peak intensity of dodecane	Peak intensity ratio	Conversion of **M1**	M_n (Experimental)	M_w (Experimental)	PDI	M_n (Theoretical)	M_w (Theoretical)
0.5	0.004798357	0.01	0.479835697	88844	127132	0.698832709	4.032860573	1146	1369	1.19	397	413
1	0.003808846	0.01	0.380884614	74535	134365	0.554720351	23.82307728	1274	1654	1.29	500	619
2	0.002689517	0.01	0.268951659	46605	118981	0.391701196	46.20966822	1440	2137	1.48	708	1035
5	0.000939886	0.01	0.093988634	20334	148548	0.136885047	81.2022731	1984	4233	2.13	2025	3669
10	0.000184357	0.01	0.018435672	4068	151510	0.026849713	96.31286557	2993	10070	3.36	10322	20264
30	2.02945E-05	0.01	0.002029455	443	149880	0.002955698	99.59410905	4557	19620	4.3	93769	187157
60	1.1649E-05	0.01	0.001164902	288	169755	0.001696563	99.76701968	4960	23746	4.78	163361	326342

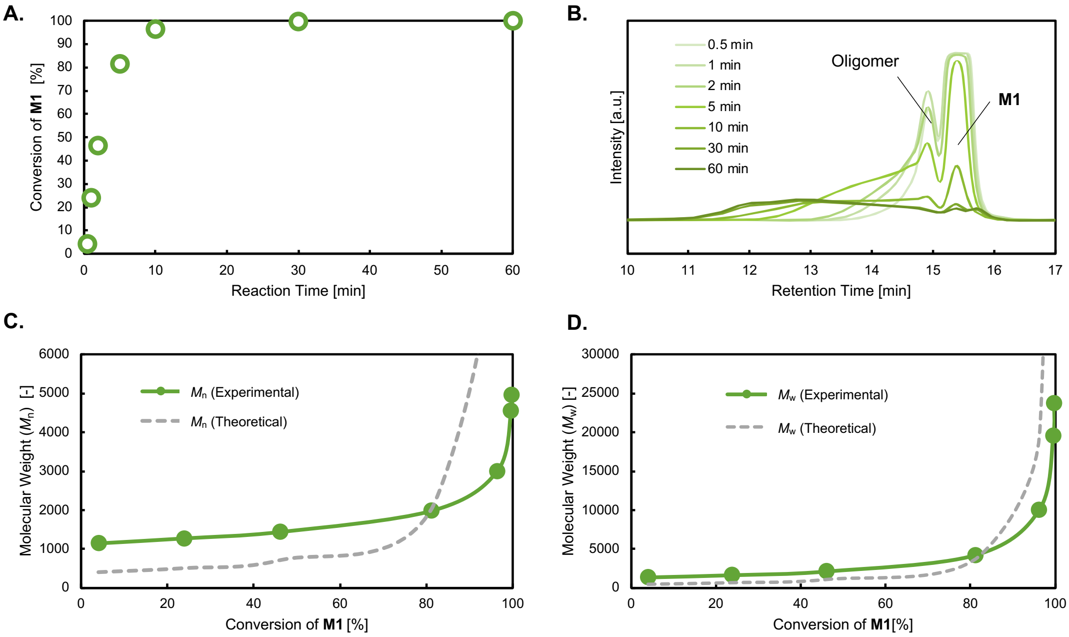

Figure S8. Time courses of the (**A**) conversion of **M1** and (**B**) GPC trace (in THF). Relationships between the conversion of **M1** and (**C**) number-average molecular weight (M_n) and (**D**) M_w. The green and dotted gray lines respectively represent the experimental data and theoretical results of stepwise polymerization.

3-3-2. [2 + 2] cycloaddition polymerization of M1 via electrochemical oxidation

Table S12. Electrochemical polymerization of **M1** using different charge passed

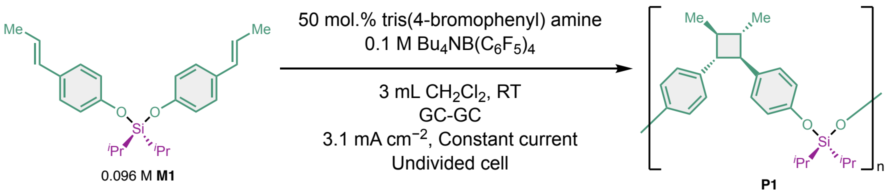

Entry	Charge passed [F mol⁻¹]	Yield of P1 [%]	M_n	M_w	PDI
**1**	0.1	35	1900	3000	1.53
**2**	0.5	80	2300	4300	1.91
**3**	1	88	4200	9700	2.31
**4**	3	78	4600	14700	3.18
**5**	6	78	2500	6200	2.48

Table S13. Electrochemical polymerization of **M1** using various supporting electrolytes

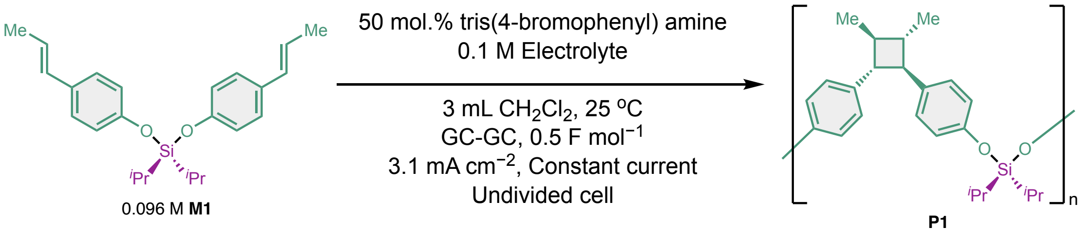

Entry	Electrolyte	Yield of P1 [%]	M_n	M_w	PDI
**1**	$^nBu_4N$ TsO	trace	-	-	-
**2**	$^nBu_4N$ TfO	trace	-	-	-
**3**	$^nBu_4N$ ClO_4	5	800	1300	1.55
**4**	$^nBu_4N$ TFSI	17	1600	2200	1.39
**5**	$^nBu_4N$ PF_6	9 a)	2200	3100	1.42
**6**	$^nBu_4N$ $B(C_6F_5)_4$	80	2300	4300	1.91

TsO^- = *p*-toluenesulfonate, TfO^- = trifluoromethanesulfonate, $TFSI^-$ = bis(trifluoromethanesulfonyl) imide.

a) A film is formed via the excessive oxidation of **M1** on the anode surface. The mass of the oxide film at the anode is not included in the yield.

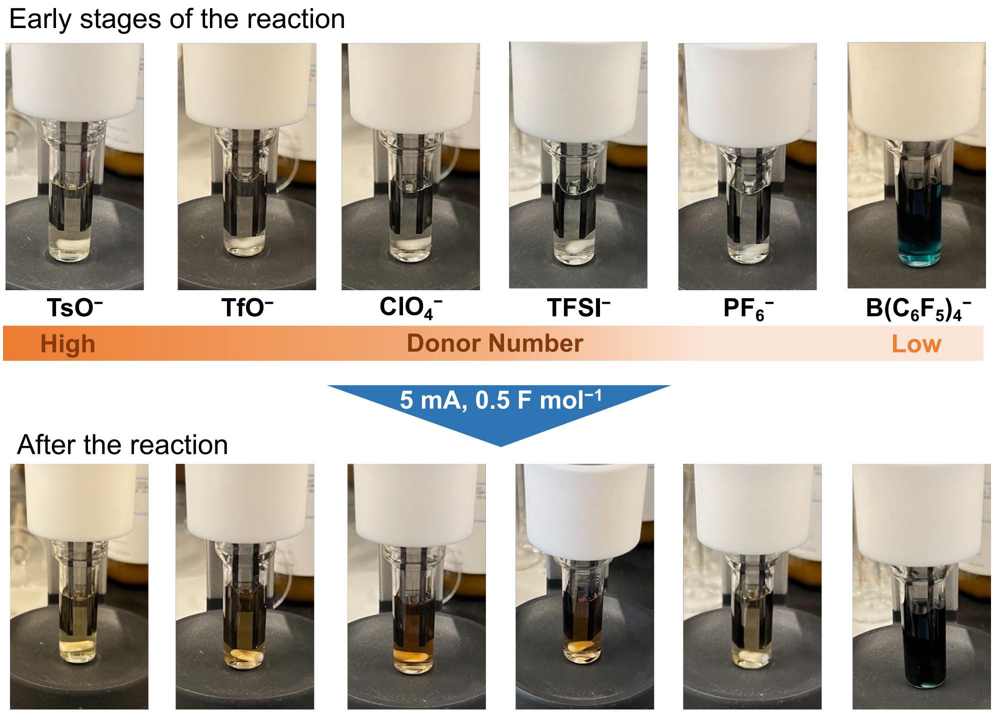

Figure S9. Differences in colors of the electrolytic solutions with various supporting electrolytes.

3-3-3. Plausible mechanism of the hole-catalytic cycloaddition polymerization

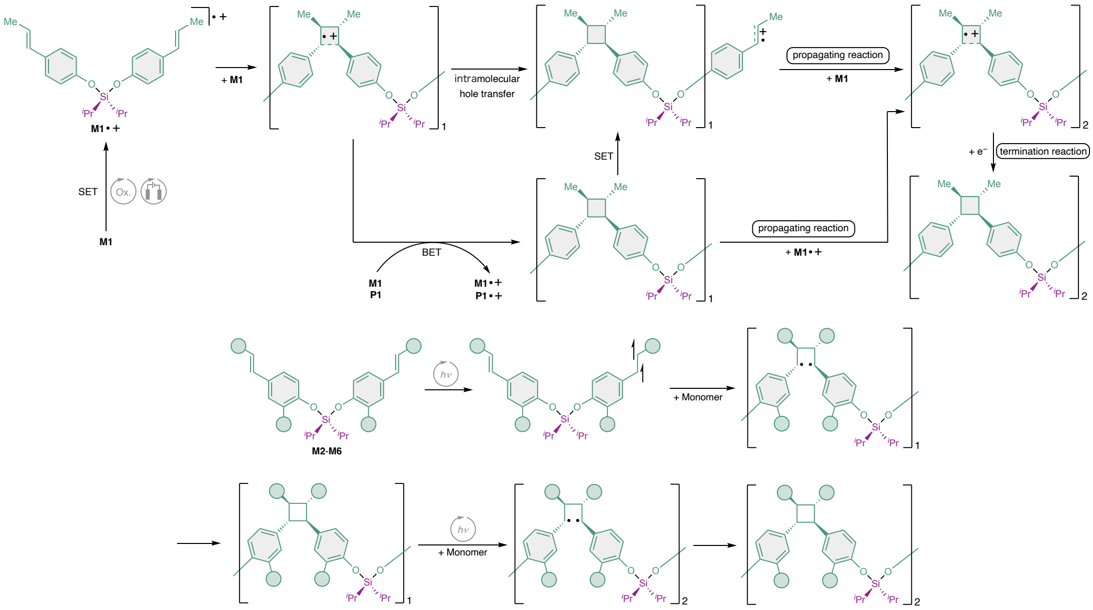

Figure S10. Plausible mechanism of the hole-catalytic cycloaddition polymerization (top) and photocatalytic polymerization (bottom).

Hole-catalytic polymerizations of **M1** proceeded catalytic amount of chemical oxidant or charge passed. This means that radical cations are effectively transferred intramolecularly or mediated by the reactants in the solution.

3-3-4. [2 + 2] cycloaddition polymerization of M2–M6

3-3-4-1. Polymerization of M2–M6 using a chemical oxidant

Table S14. Polymerization of **M2–M6** using various single-electron oxidants

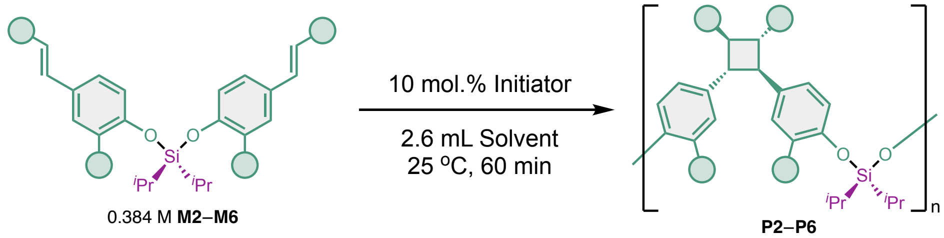

Entry	Substrate	Initiator	Solvent	Yield [%]	M_n	M_w	PDI
**1**	**M2**	Magic blue	CH_2Cl_2	trace	-	-	-
**2**	**M3**	Magic blue	CH_2Cl_2	trace	-	-	-
**3**	**M4**	Magic blue	CH_2Cl_2	trace	-	-	-
**4**	**M5** a)	Magic blue	CH_2Cl_2	trace	-	-	-
**5**	**M6**	Magic blue	CH_2Cl_2	N.D.	-	-	-
**6**	**M3**	PIDA	CH_2Cl_2	N.D.	-	-	-
**7**	**M3**	PIDA	HFIP	N.D.	-	-	-
**8**	**M3**	PIFA b)	HFIP	N.D.	-	-	-
**9**	**M3**	IBX c)	HFIP	N.D.	-	-	-
**10**	**M3**	DMP d)	HFIP	N.D.	-	-	-

a) 0.192 M **M5**. b) [Bis(trifluoroacetoxy)iodo]benzene. c) 2-Iodoxybenzoic acid. d) Dess-Martin periodinane.

3-3-4-2. Polymerization of M2–M6 using a chemical oxidant

We performed electrochemical polymerizations of **M3**, **M4**, and **M6**. However, no polymeric products were observed when tris(4-bromophenyl) amine was used. Meanwhile, when **M6** was polymerized using tris(2,4-dibromophenyl) amine, which has a higher oxidation potential than tris(4-bromophenyl) amine (Figure S2), **P6** was obtained but its yield was only 7% (Figure S11). Also, **P3** and **P4** could not be obtained at all even though tris(2,4-dibromophenyl) amine was used. These results are presumably due to the lack of nucleophilicities of the monomers, making it difficult to proceed hole-catalytic [2 + 2] cycloaddition reactions.

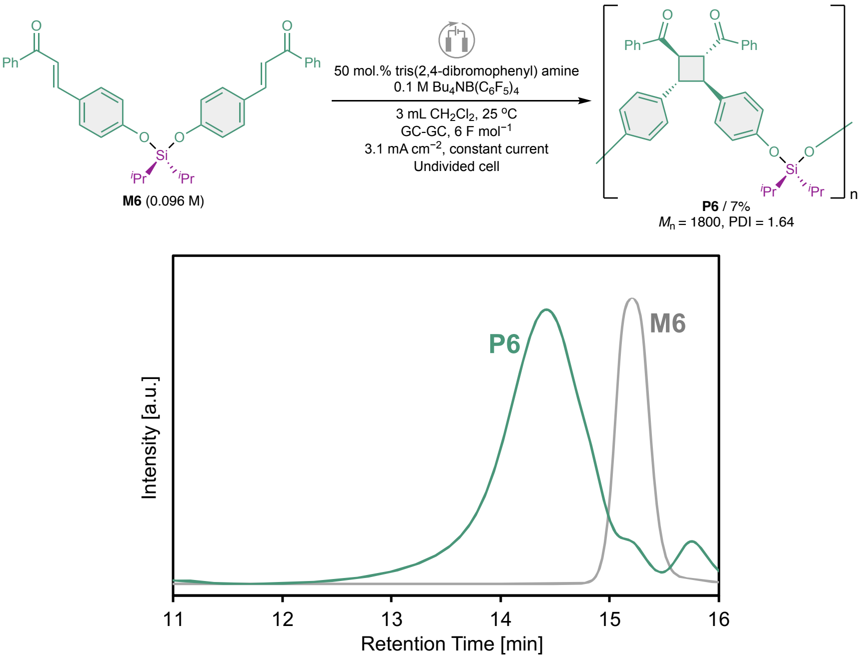

Figure S11. GPC traces (in THF) of **M6** and electrochemical polymerized **P6**.

3-3-4-3. Polymerization of M3 using various photocatalysts

Table S15. Polymerization of **M3** using various photocatalysts

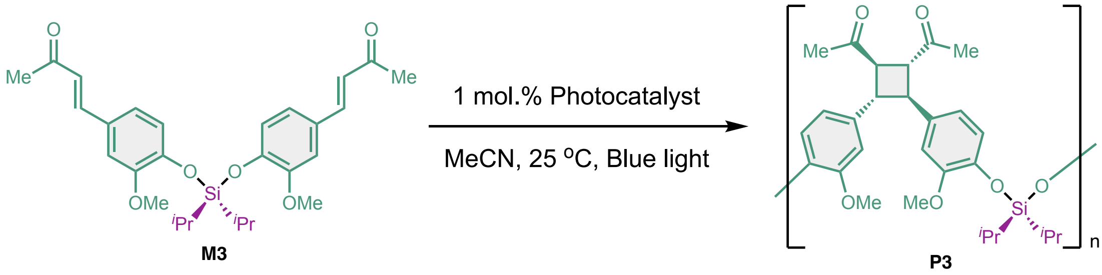

Entry	Concentration [M]	Photocatalyst	Time [h]	Yield [%]	M_n	M_w	PDI
**1**	0.384	Acridinium Perchlorate a)	12	7	1100	1100	1.08
**2** b)	0.384	$[Ru(bpz)_3][PF_6]_2$	12	3	1100	1300	1.19
**3**	0.384	9-Fluorenone c)	30	48	1400	1800	1.31
**4**	0.192	Ir- F	12	59	1700	2400	1.40
**5**	0.384	Ir- F	1	trace	-	-	-
**6**	0.384	Ir- F	12	79	3000	6900	2.34
**7**	0.384	Ir- F	17	81	3200	13300	4.22
**8**	0.384	Ir- F	30	Unmeasurable d)	-	-	-
**9**	0.384	Ir- F	60	Unmeasurable d)	-	-	-
**10**	0.768	Ir- F	12	74	2100	3500	1.66

Ir-F = (4,4'-Di-tert-butyl-2,2'-bipyridine)bis[3,5-difluoro-2-[5-(trifluoromethyl)-2-pyridinyl]phenyl]iridium(III) Hexafluorophosphate.

a) 9-mesityl-10-methylacridinium perchlorate. b) Visible light. c) Photocatalyst (10 mol.%). d) Gelatinous components, which are insoluble in any solvent, are formed. MeCN, acetonitrile.

3-3-4-4. Polymerization of M2 using the Ir-F photocatalyst

Table S16. Polymerization of **M2** using the Ir-F photocatalyst

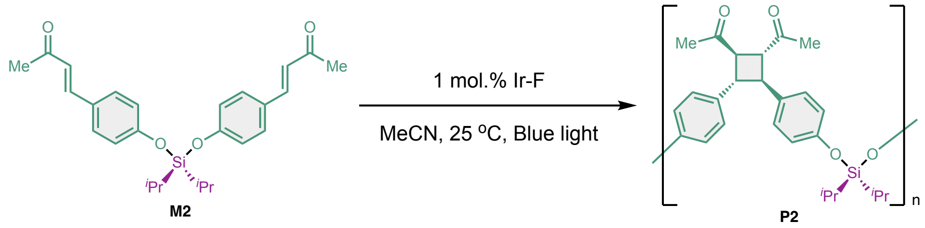

Entry	Concentration [M]	Time [h]	Yield [%]	M_n	M_w	PDI
**1**	0.384	12	35	1200	2000	1.57
**2**	0.384	60	92	4100	21600	5.20
**3**	0.768	60	84	4900	14000	2.84

3-3-4-5. Polymerization of M4 using the Ir-F photocatalyst

Table S17. Polymerization of **M4** using the Ir-F photocatalyst

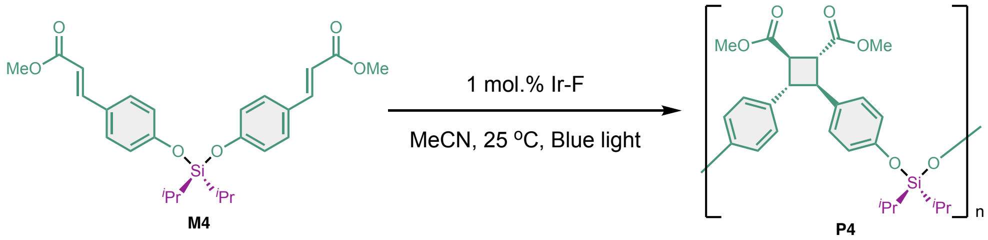

Entry	Concentration [M]	Time [h]	Yield [%]	M_n	M_w	PDI
**1**	0.192	12	28	3100	4200	1.38
**2**	0.192	60	61	7600	17400	2.27
**3**	0.384	12	53	2500	4300	1.72
**4**	0.384	60	79	4600	16800	3.66
**5**	0.768	60	79	6200	29900	4.84

3-3-4-6. Polymerization of M5 using the Ir-F photocatalyst

Table S18. Polymerization of **M5** using the Ir-F photocatalyst

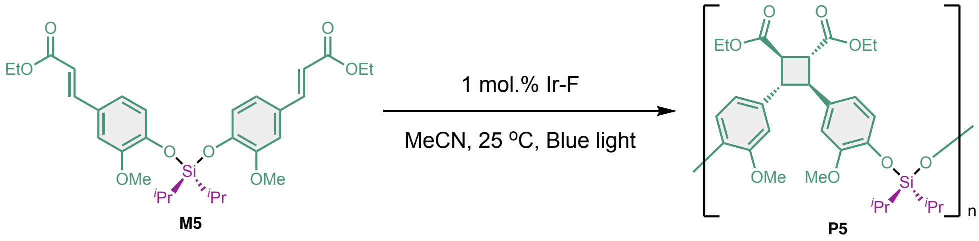

Entry	Concentration [M]	Time [h]	Yield [%]	M_n	M_w	PDI
**1**	0.384	12	61	7700	15400	1.98
**2**	0.384	60	67	5000	12300	2.47
**3**	0.768	60	73	10600	25800	2.43

3-3-4-7. Polymerization of M6 using the Ir-F photocatalyst

Table S19. Polymerization of **M6** using the Ir-F photocatalyst

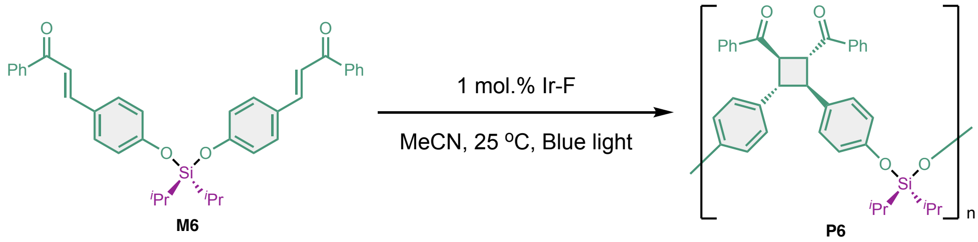

Entry	Concentration [M]	Time [h]	Yield [%]	M_n	M_w	PDI
**1**	0.192	12	75	2100	5200	2.50
**2**	0.384	12	76	3000	6300	2.06
**3**	0.384	60	80	4600	35600	7.74

3-3-4-8. Investigation of photocatalytic polymerization mechanism using M4

3-3-4-8-1. General procedure used in investigating the polymerization mechanism

(i) Calibration curve obtained using HPLC analyses

Standard solutions were prepared by dissolving specific amounts of **M4** (2.0, 1.0, 0.5, 0.25, and 0.125 mM) and 1,2,4-trimethoxybenzene (2 mM) as the internal standard in acetonitrile. The peak intensities of **M4** and 1,2,4-trimethoxybenzene in the liquid chromatogram of each standard solution were analyzed to construct a calibration curve that revealed the relationship between the ratios of the peak intensities and concentrations of **M4**/1,2,4-trimethoxybenzene. The calibration curve indicates that (peak intensity ratio) = 49.671 × (concentration ratio) and R^2 = 0.9992 (Table S20, Figure S12).

Table S20. Data used in constructing the calibration curve of the polymerization behavior of **M4**

Conc. of **M4** [M]	Conc. of internal standard [M]	Conc. ratio	Peak intensity of **M4**	Peak intensity of internal standard	Peak intensity ratio
0.0005004	0.002	0.2502027	12586399	1025874	12.26895213
0.0002502	0.002	0.1251014	6679627	1045998	6.385888883
0.0001251	0.002	0.0625507	3104917	960761	3.231726725
0.0000626	0.002	0.0312753	1656917	910503	1.819782033
0.0000313	0.002	0.0156377	894922	983847	0.909615011

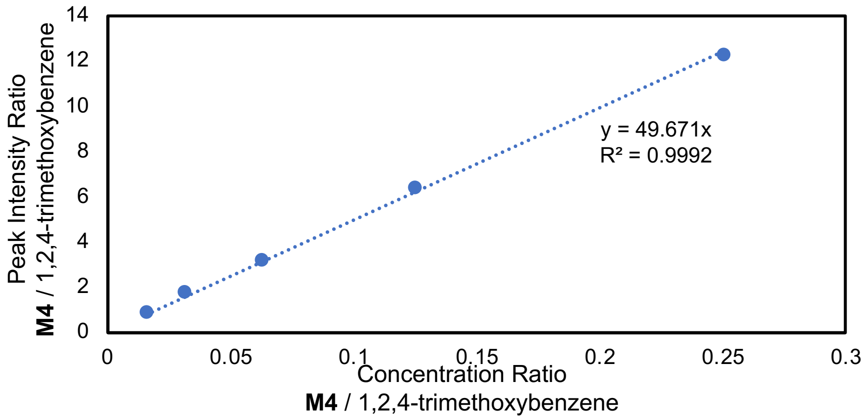

Figure S12. Calibration curve of the polymerization behavior of **M4**

(ii) Investigation of the polymerization behavior

In a 5 mL vial, Ir-F dissolved in 1 mL of acetonitrile was added dropwise to **M4** (719.4 mg, 1.54 mmol) and 1,2,4-trimethoxybenzene (298.7 µL, 2 mmol) as an internal standard dissolved in 1 mL of acetonitrile. The reaction mixture was stirred for a defined period under blue light. At predetermined times after the reaction started, 10 µL of the reaction solution was sampled and diluted with 5 mL of acetonitrile. The conversion of **M4** in each diluted solution was determined using LC, and the M_n and M_w were determined using GPC. As a result, it showed a great increase of M_n and M_w in the region where the conversion ratio of **M4** is high. These data strongly suggest that the reaction proceeds via a stepwise mechanism.

Table S21. Polymerization behavior of **M4**, as determined using LC and GPC

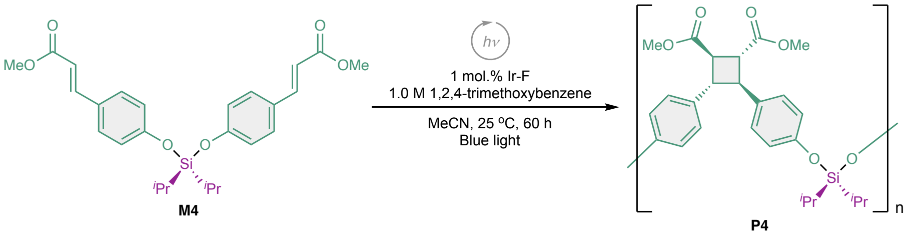

min	h	Conc. of **M4** [M]	Conc. of internal standard [M]	Concentration ratio	Peak intensity of **M4**	Peak intensity of internal standard	Peak intensity ratio	Conversion of **M4**	M_n (Experimental)	M_w (Experimental)
5	0.0833	0.000409146	0.002	0.204573136	4098744	403366	10.16135	73.4	987	1036
10	0.1667	0.000381879	0.002	0.190939696	4242144	447287	9.48417	75.1	1019	1092
30	0.5	0.00029421	0.002	0.14710504	4358261	596462	7.30685	80.8	1012	1076
75	1.25	0.000216237	0.002	0.108118493	2823109	525684	5.37035	85.9	1032	1124
120	2	0.000135794	0.002	0.067897163	1561298	462947	3.37252	91.2	1118	1302
240	4	9.33513E-05	0.002	0.046675656	1191240	513814	2.31843	93.9	1212	1486
360	6	6.15429E-05	0.002	0.030771459	793233	518979	1.52845	96.0	1394	1794
540	9	3.13743E-05	0.002	0.015687167	363201	466122	0.77920	98.0	1562	2157
1425	23.75	4.05924E-06	0.002	0.002029621	48343	479530	0.10081	99.7	2376	4632
1830	30.5	2.65853E-06	0.002	0.001329263	37037	560947	0.06603	99.8	2700	6025
3192	53.2	0	0.002	0	0	401171	0.00000	100.0	2981	8543
3600	60	0	0.002	0	0	529856	0.00000	100.0	2977	9732

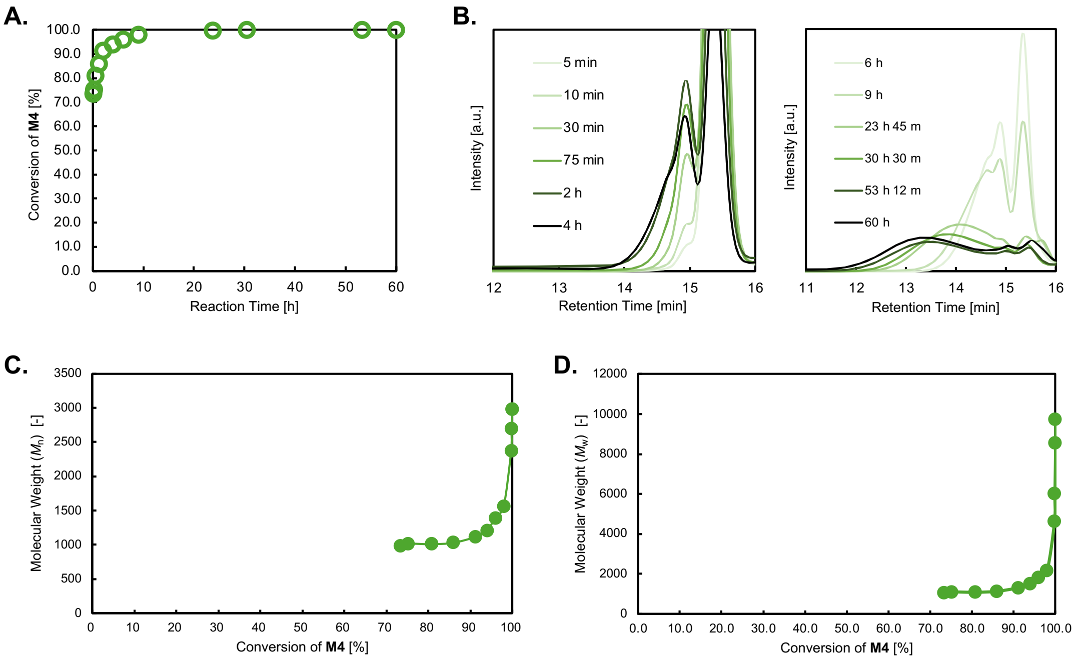

Figure S13. Time courses of the (**A**) conversion of **M4** and (**B**) GPC trace (in THF). Relationships between the conversion of **M4** and (**C**) number- (M_n) and (**D**) weight-average molecular weight (M_w).

3-3-5. Confirmation of terminal structures and repeating units of P1–P6 by MALDI-TOF-MS spectra

MALDI-TOF-MS spectra of the polymers (except for **P5**) consisted of the sharp peaks separated by the masses of the repeating units. However, the masses of the terminal structures calculated from these peaks did not match the values of the desired structures. In addition, as for **P5**, clear MS spectrum was not obtained. These results are presumably due to lacks of compatibility with the matrix or the way the samples were dried.

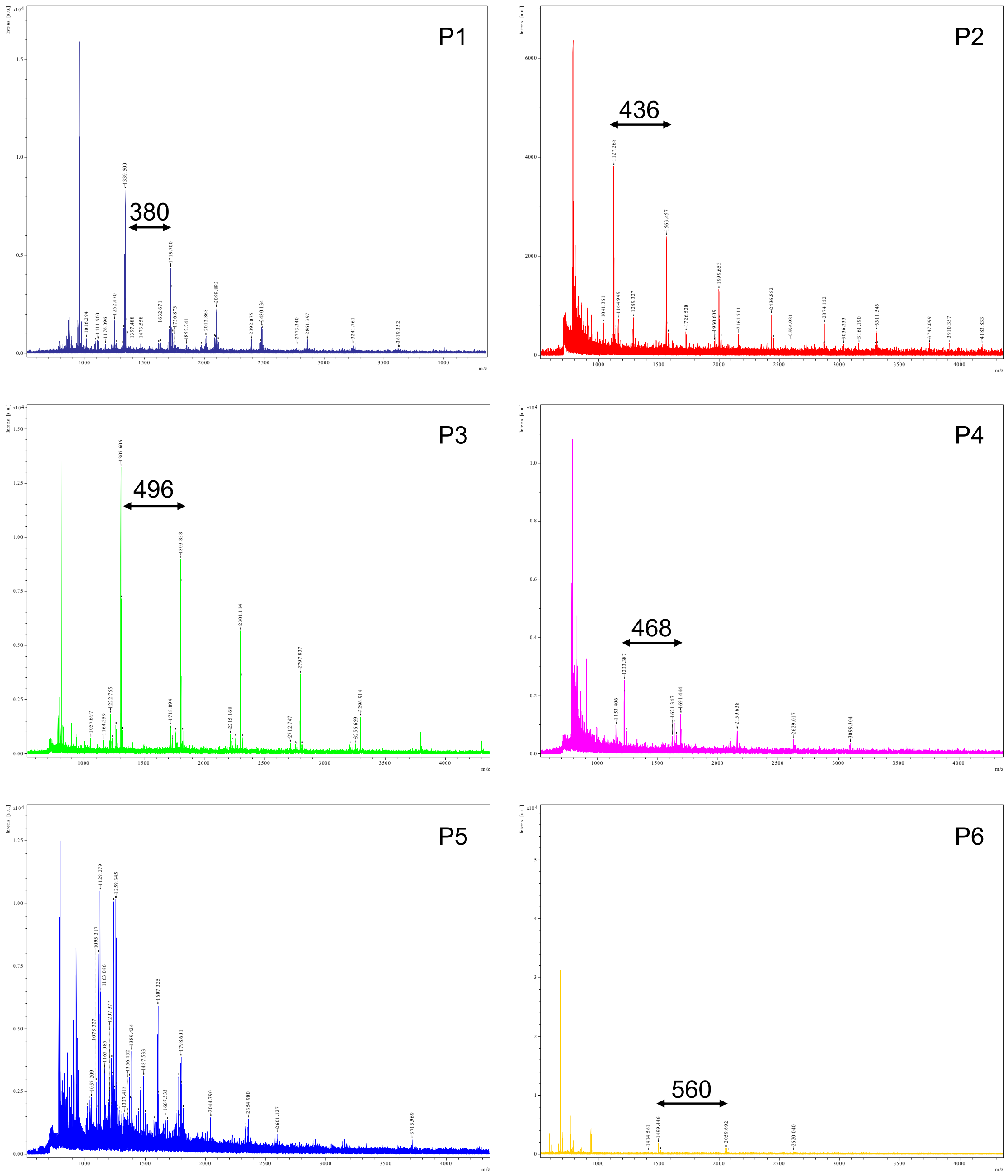

Figure S14. MALDI-TOF-MS spectra of **P1–P6**.

3-3-6. Calculations of the molecular weights of P1–P6 using ^{1}H NMR measurements

3-3-6-1. Calculation of the molecular weight of P1 synthesized using PIDA

The relationship between "the number of the protons of repeating unit and terminal olefins" and "integral value(s) of them" is defined as the equation below,

$$\frac{\text{the number of the protons of repeating unit} \times \text{n}}{\text{the number of the protons of terminal structure}}$$

$$= \frac{\text{sum of the integral values of the protons of repeating unit}}{\text{sum of the integral values of the protons of terminal structure}}$$

where n is the number of the repeating units. As for **P1**, since most of the proton peaks of the repeating unit and terminal structure (Figure S15, positions: a, b, d, e, f, g, a', d', e') were too close to other peaks, the integral values of the benzylic and olefinic protons (Figure S15, position c, b', c') was used for the calculation. The molecular weight of **P1** was determined to be 3600 from the following equation.

$$\frac{2\text{n}}{4} = \frac{2.00\ (\text{position c})}{(0.23\ (\text{position b}') + 0.25\ (\text{position c}'))}$$

$$\text{n} = \frac{4}{0.48}$$

$$\text{molecular weight of } \mathbf{P1}$$

$$= (\text{molecular weight of the repeating unit} \times \text{n}) + \text{molecular weight of the terminal structure}$$

$$= (380.60 \times \text{n}) + 380.60$$

$$\approx 3552$$

$$\approx 3600$$

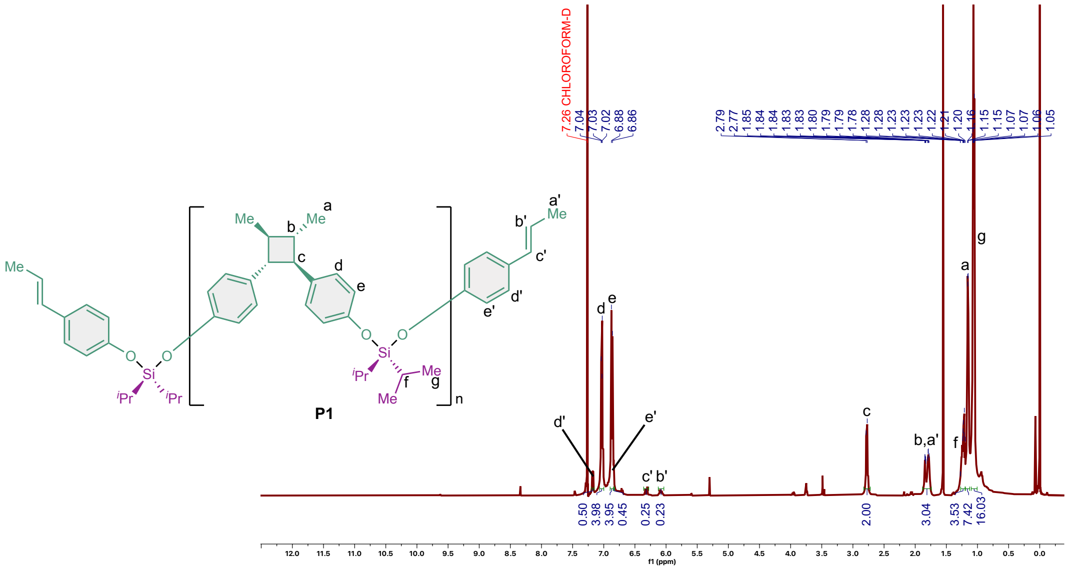

Figure S15. ^{1}H NMR spectrum of **P1** synthesized using PIDA (including terminal structure) [500 MHz, CHLOROFORM-*D*].

3-3-6-2. Calculation of the molecular weight of P3

As for **P3**, we could observe the terminal olefin peak in a sample with a small PDI value (Table S15 Entry 6). Since most of the proton peaks of the repeating unit and terminal structure of **P3** (Figure S16, positions: d, e, f, g, h, i, d', e', f', g') were too close to other peaks, the integral values of the other peaks (Figure S16, position a, b, c, a', b', c') were used for the calculation. The molecular weight of **P3** was determined to be 5900 from the following equation.

$$\frac{10\text{n}}{10} = \frac{(6.32\ (\text{position a}) + 1.96\ (\text{position b}) + 2.03\ (\text{position c}))}{(0.51\ (\text{position a}') + 0.25\ (\text{position b}') + 0.18\ (\text{position c}'))}$$

$$\text{n} = \frac{10.31}{0.94}$$

$$\text{molecular weight of } \mathbf{P3}$$

$$= (\text{molecular weight of the repeating unit} \times \text{n}) + \text{molecular weight of the terminal structure}$$

$$= (496.68 \times \text{n}) + 496.68$$

$$\approx 5944$$

$$\approx 5900$$

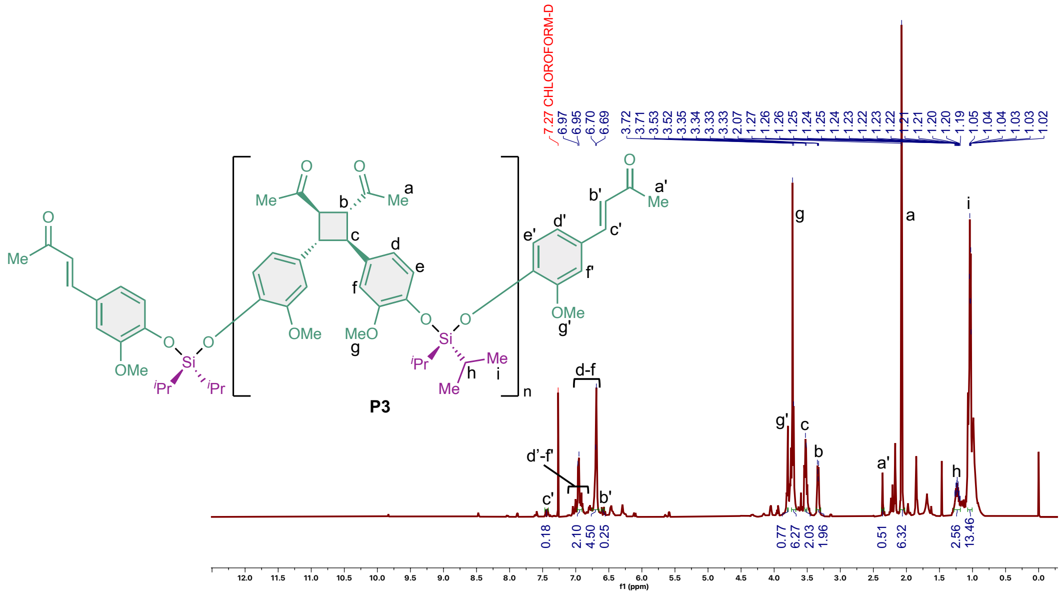

Figure S16. ^{1}H NMR spectrum of **P3** (including terminal structure) [500 MHz, CHLOROFORM-*D*].

3-3-6-3. Calculation of the molecular weights of the other polymers

As for the other polymers (**P2**, **P4**, **P5**, **P6**), absolute molecular weights could not be determined since their proton peaks of the terminal structures were not observed or too close to other peaks. This disappearances of the peaks in the terminal structures were presumably due to the undesired side reactions such as [2 + 2] cycloadditions between terminal olefins of the polymers. If this kind of reactions occur, cyclic polymers are formed, which means the terminations of the polymerization reactions. In addition, if such termination reactions occur independently of the molecular weights of the polymers, PDIs of the polymers are estimated to increase, which is consistent with the relatively large PDIs of **P2**, **P4**, **P5**, and **P6** (Figure 2I).

3-3-7. Elucidations of selectivity from ^{1}H NMR measurements of P1–P6

Although higher-order coupling were not analyzable for all polymers, analysis of **P1** and **P2** revealed a coupling constant of approximately J = 9 Hz (Figure S17), which is consistent with the results obtained from the decomposition products (Figure 4F).

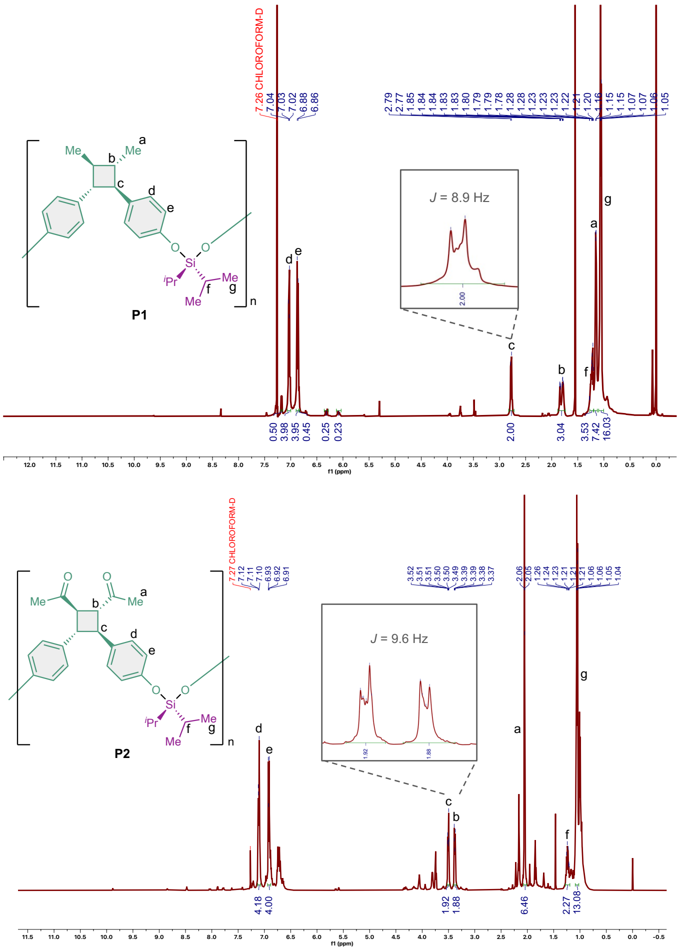

Figure S17. The coupling constants of the adjacent vicinal protons of methine groups of **P1** and **P2** in $CDCl_3$.

3-4. Thermal properties of the upcycled materials

3-4-1. Thermal properties of epoxy cured resins 1 and 2

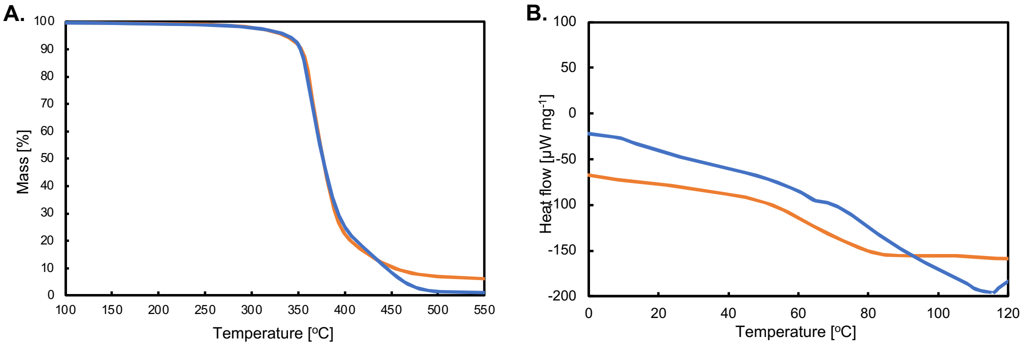

C.

Epoxy cured resin	T_{d5} [°C]	T_g [°C]
**1**	337	52
**2**	339	61

Figure S18. (**A**) Thermogravimetric analysis (TGA) and (**B**) differential scanning calorimetry (DSC) thermograms of epoxy cured resins **1** (orange) and **2** (blue). Heat flow is shown in the endothermic down direction. (**C**) T_{d5} and T_g values of epoxy cured resins 1 and 2.

3-4-2. Thermal properties of polyurethane

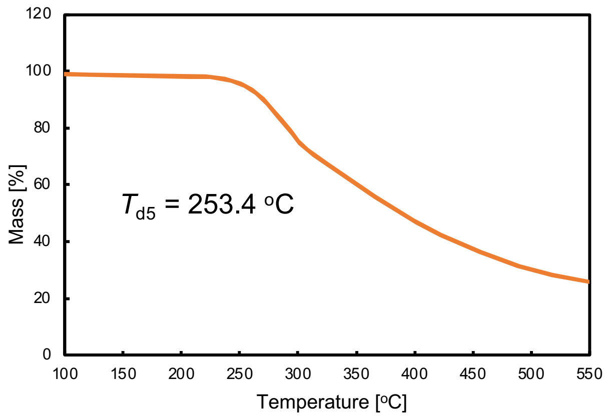

Figure S19. Thermogravimetric analysis (TGA) thermogram of polyurethane.

3-5. Decomposition of P1–P6

3-5-1. Diels-Alder decomposition of P1

3-5-1-1. Optimization of Diels-Alder decomposition

Table S22. Diels-Alder decomposition of **P1** under various conditions

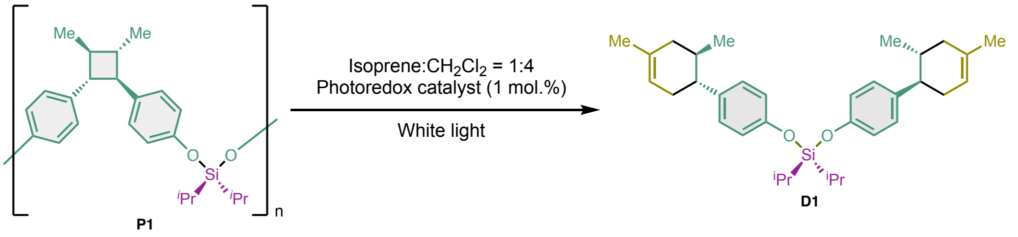

Entry	Photoredox catalyst	Reaction Time [h]	Temperature [°C]	Yield of D1 [%]
1	$[Ru(bpz)_3](PF_6)_2$	12	0	N.D.
2	$[Ru(bpz)_3][B(C_6F_5)_4]_2$	12	0	N.D.
3	$[Ru(bpz)_3][B(C_6F_5)_4]_2$	12	25	trace
4	$[Ru(bpz)_3][B(C_6F_5)_4]_2$	60	25	16

Table S23. Diels-Alder decomposition of **P1** using different solvents

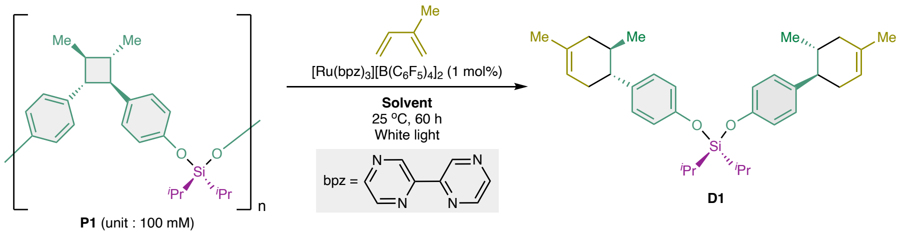

Entry	Solvent	Yield of D1 [%]
1	CH_2Cl_2	16
2	CH_2Cl_2 / HFIP (v/v = 1/1)	33

Table S24. Diels-Alder decomposition of **P1** using different photoredox catalysts

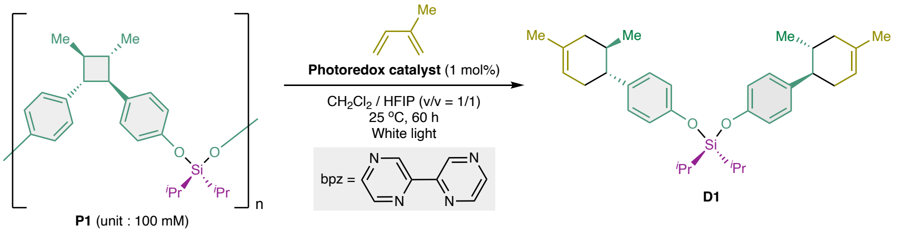

Entry	Photoredox catalyst	Light irradiation	Yield of D1 [%]
**1**	$[Ru(bpz)_3][B(C_6F_5)_4]_2$	White light	33
**2**	Eosin Y	White light	29
**3**	9-mesityl-10-methylacridinium perchlorate	White light	36
**4**	Ir-F	Blue light	4
**5**	$Ir(ppy)_3$	Blue light	8
**6**	2,4,6-tris(4-methoxyphenyl)pyrylium tetrafluoroborate	Blue light	36

Table S25. Diels-Alder decomposition of **P1** with different concentration of **P1** and/or isoprene

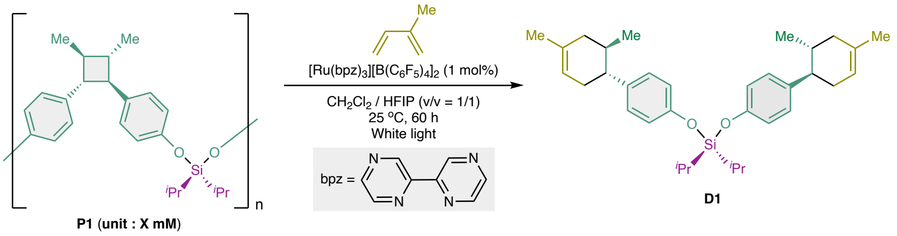

Entry	Concentration of P1 unit [mM]	Amount of isoprene [equiv.]	Yield of D1 [%]
**1**	100	20	33
**2**	55	20	51
**3**	55	4	62
**4**	30	20	63
**5**	30	4	45

3-5-2. Decomposition at the linker moieties

3-5-2-1. GPC traces before/after the decomposition reactions

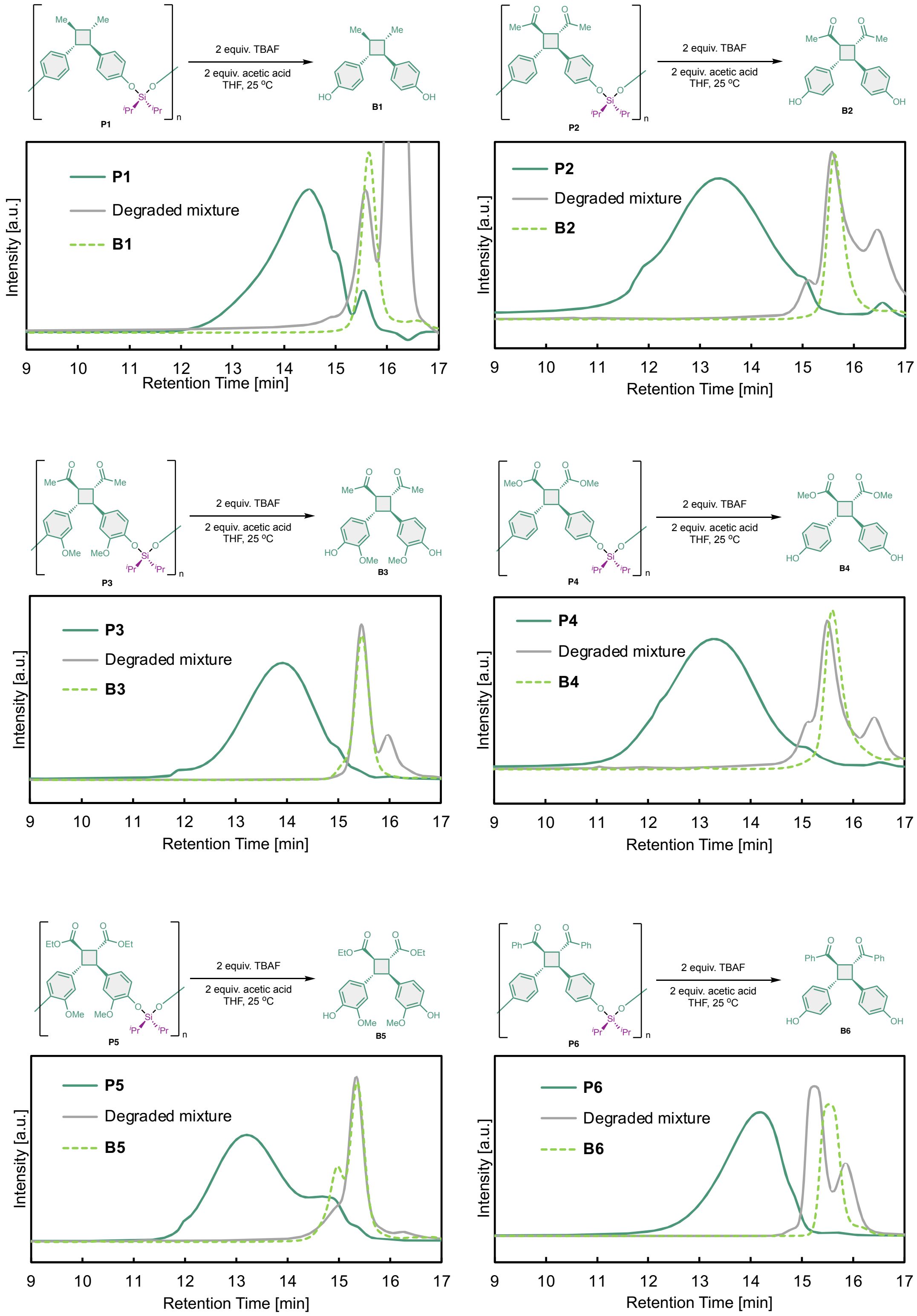

Figure S20. GPC traces (in THF) before/after the decomposition of **P1–P6**.

3-5-2-2. Decomposition of P1 synthesized via electrolysis

Table S26. Decomposition of **P1** synthesized via electrolysis using different charge passed

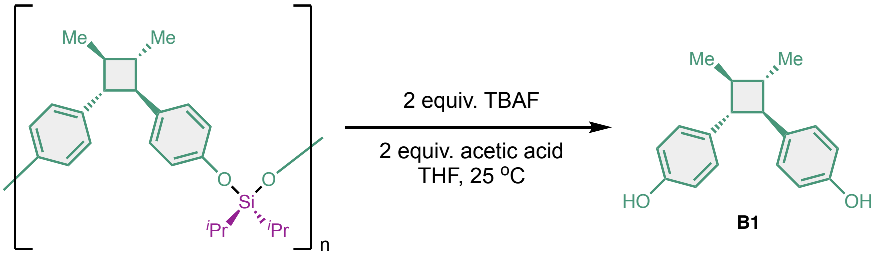

P1 (synthesized via electrolysis)

Entry	P1 used in the decomposition reaction a)				Yield of B1 [%]
	Charge passed [F mol⁻¹]	M_n	M_w	PDI	
**1**	0.1	1900	3000	1.53	51
**2**	0.5	2300	4300	1.91	50
**3**	1	4200	9700	2.31	28
**4**	3	4600	14700	3.18	3
**5**	6	2500	6200	2.48	trace

a) Polymerization conditions: substrate, 0.096 M **M1**; mediator, 50 mol.% of tris(4-bromophenyl) amine; 0.1 M $Bu_4NB(C_6F_5)_4$ as the supporting electrolyte; solvent, 3 mL CH_2Cl_2; anodic electrode, glassy carbon (0.8 × 2 cm); cathodic electrode, glassy carbon (0.8 × 2 cm); 5 mA of current; room temperature; undivided cell.

3-5-2-3. Instability of the decomposition product

GC-MS analysis of the crude mixture a few days after the decomposition reaction of **P1** revealed the presence of several byproducts that were not observed in the chromatogram obtained immediately after the decomposition reaction (Figure S21). This result indicates that the decomposition products are unstable, which is thought to be one of the reasons hampering the improvement of the yields of the decomposition products.

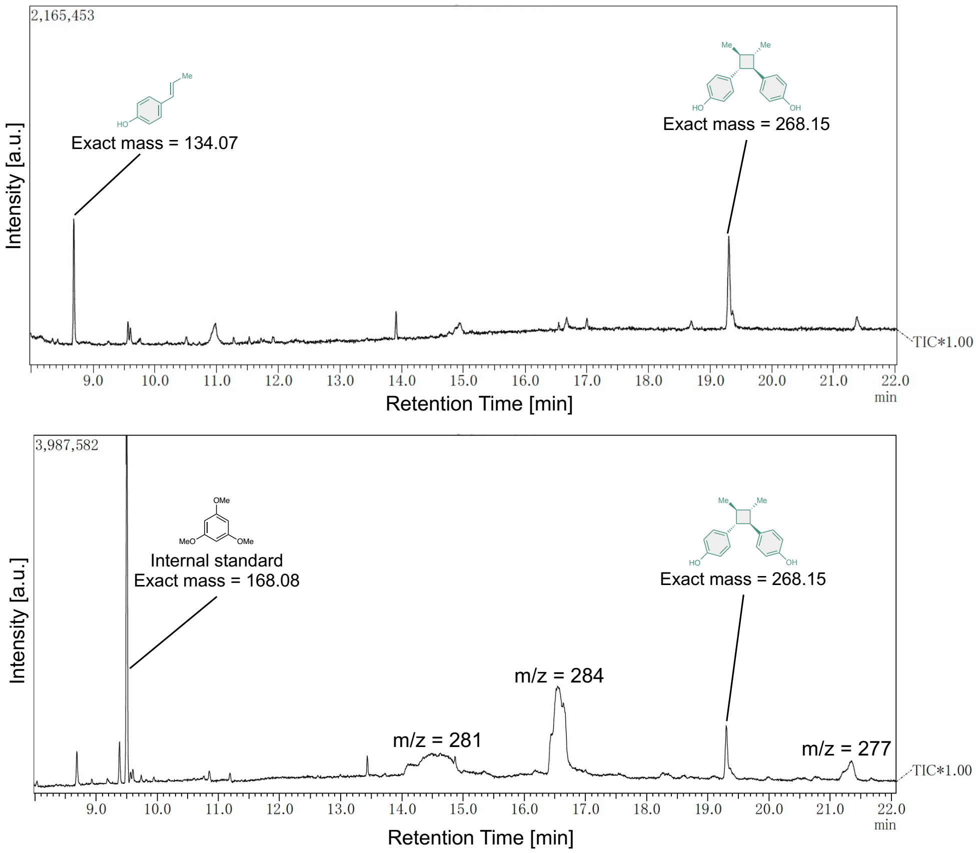

Figure S21. GC-MS chromatogram of the crude mixtures immediately after (top) and several days after (bottom) the decomposition reaction of **P1**.

3-5-3. Recycling and upcycling of B1

3-5-3-1. Recycling of B1

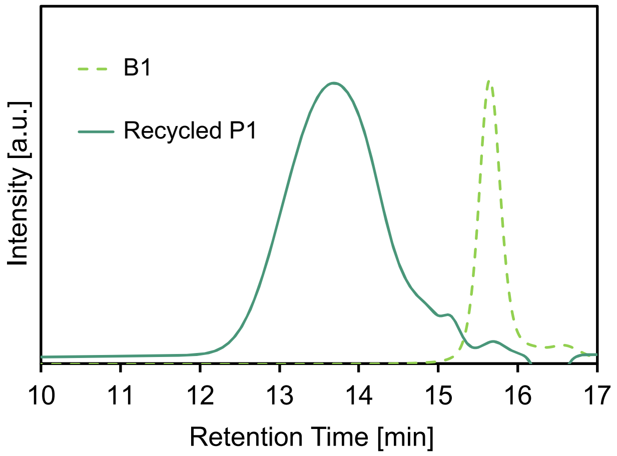

Figure S22. GPC traces (in THF) before/after the recycling of **B1**.

3-5-3-2. Upcycling of B1

3-5-3-2-1. Synthesis of polyurethane using B1

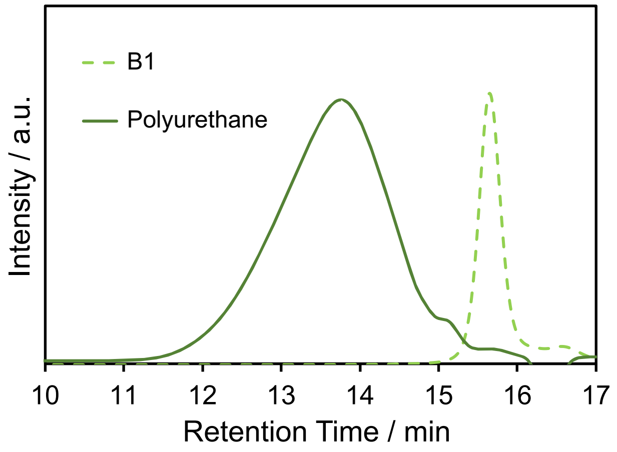

Figure S23. GPC traces (in THF) before/after the synthesis of **polyurethane**.

4. Visual Data

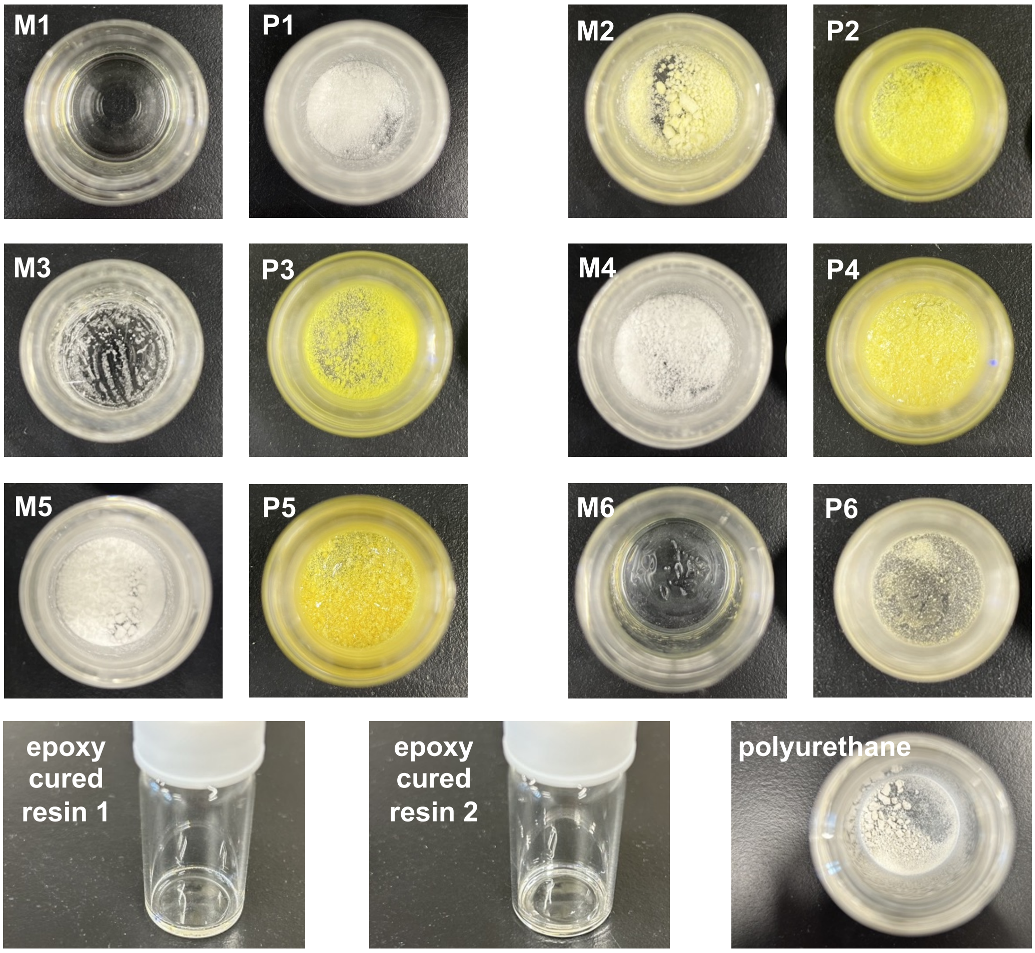

Figure S24. Images of the monomers, polymers, and upcycled materials.

5. NMR Spectra

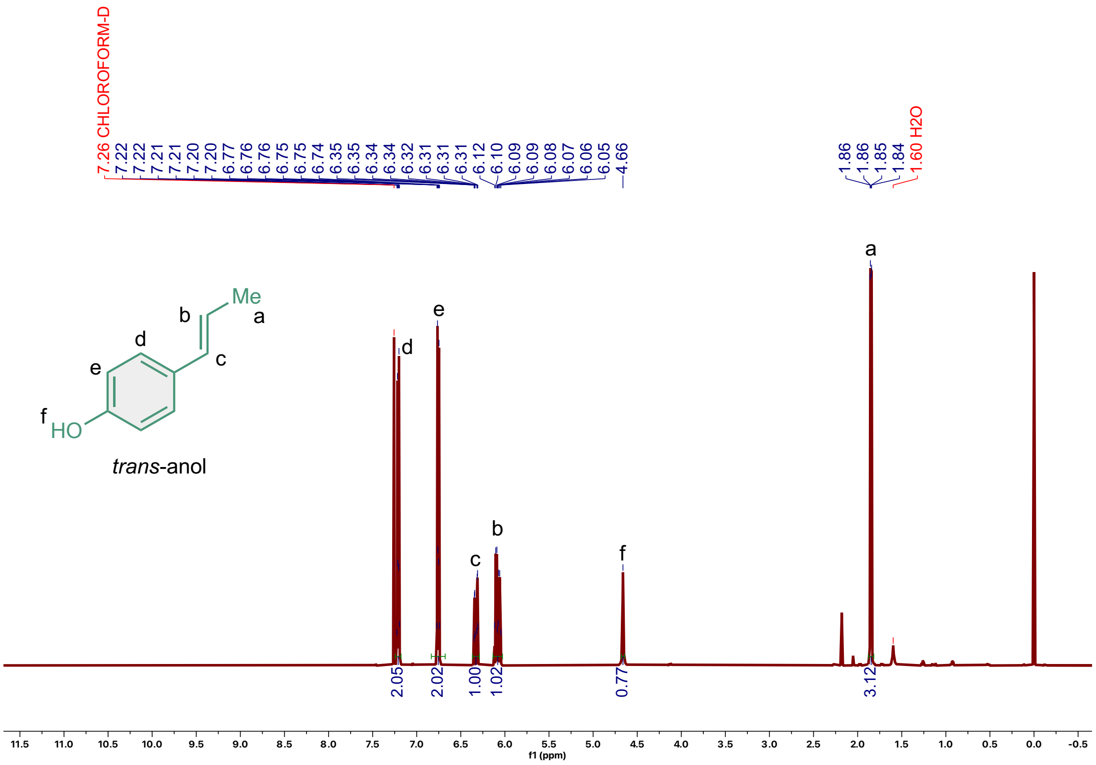

Figure S25. ^{1}H NMR spectrum of *trans*-anol [500 MHz, CHLOROFORM-*D*].

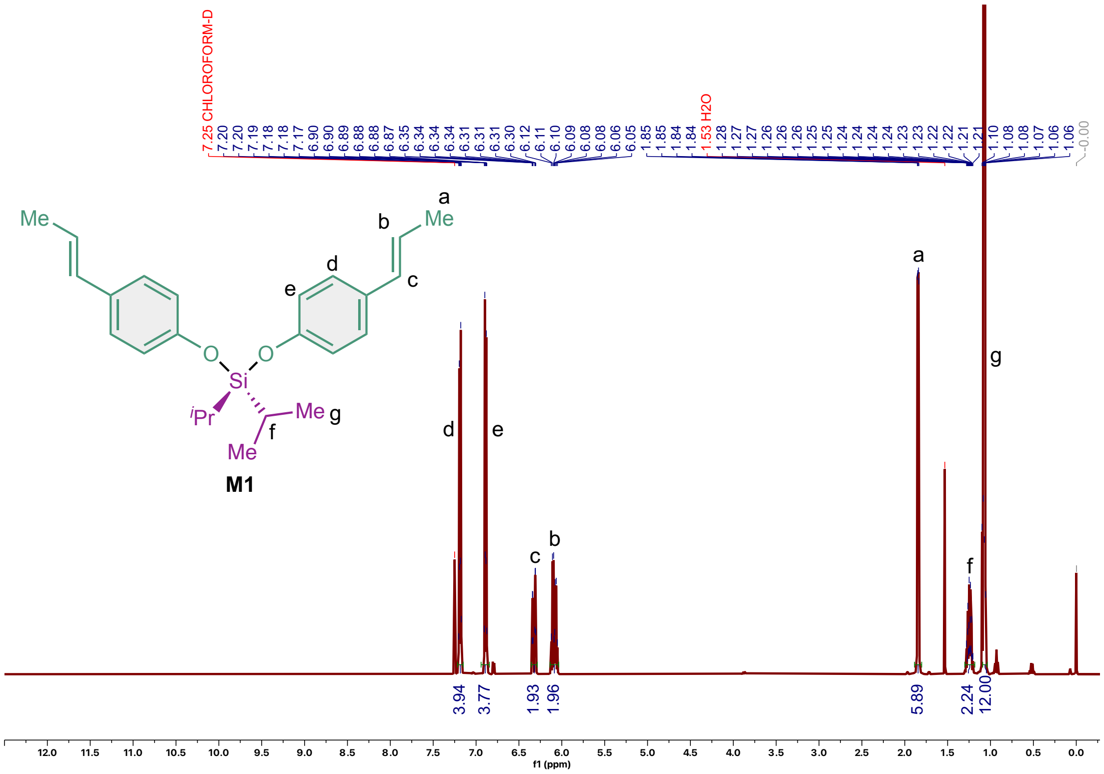

Figure S26. ^{1}H NMR spectrum of **M1** [500 MHz, CHLOROFORM-*D*].

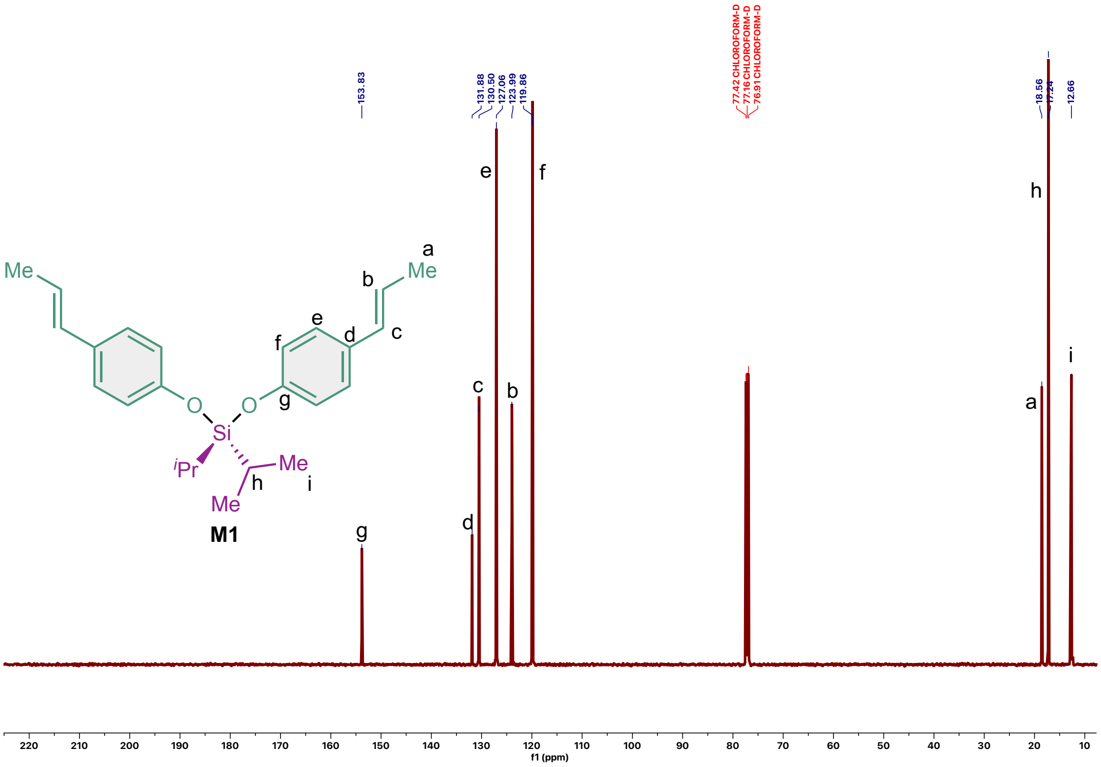

Figure S27. ^{13}C NMR spectrum of **M1** [126 MHz, CHLOROFORM-*D*].

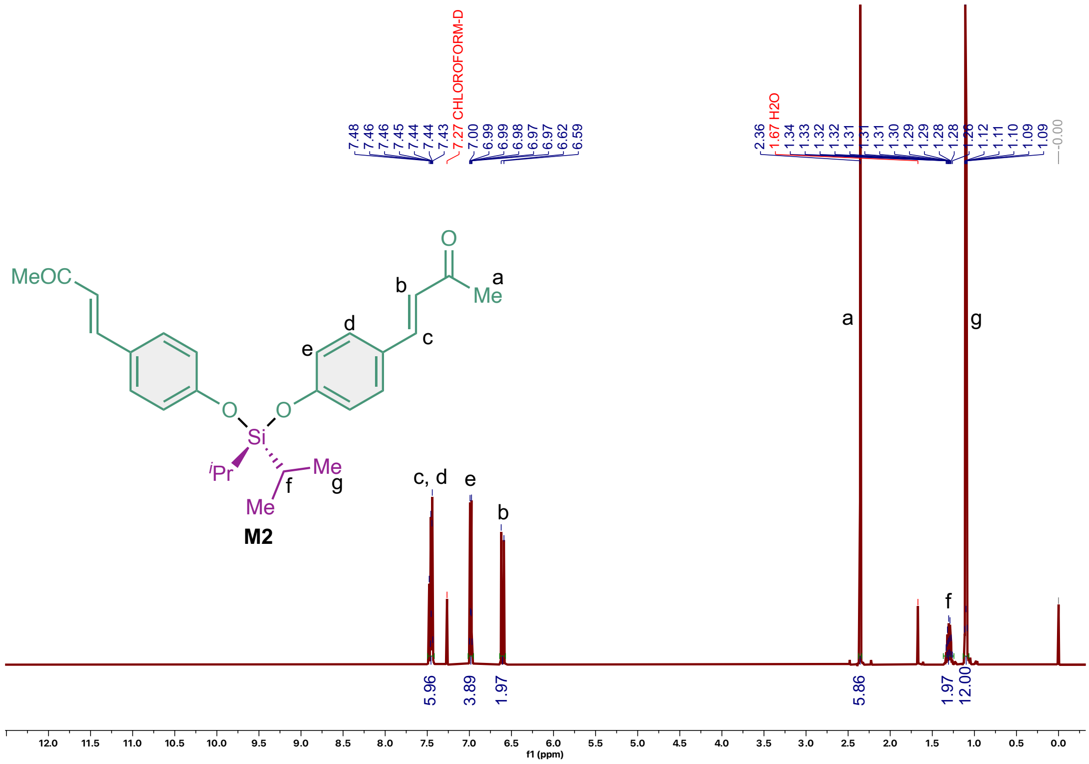

Figure S28. ^{1}H NMR spectrum of **M2** [500 MHz, CHLOROFORM-*D*].

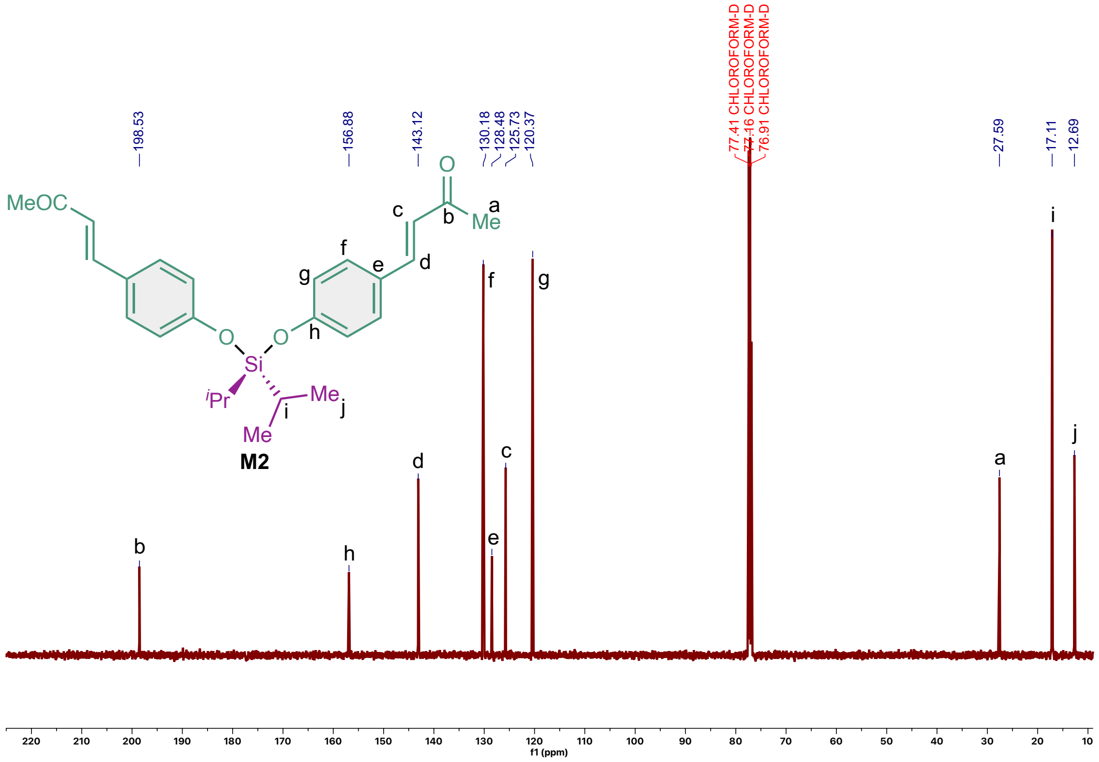

Figure S29. ^{13}C NMR spectrum of **M2** [126 MHz, CHLOROFORM-*D*].

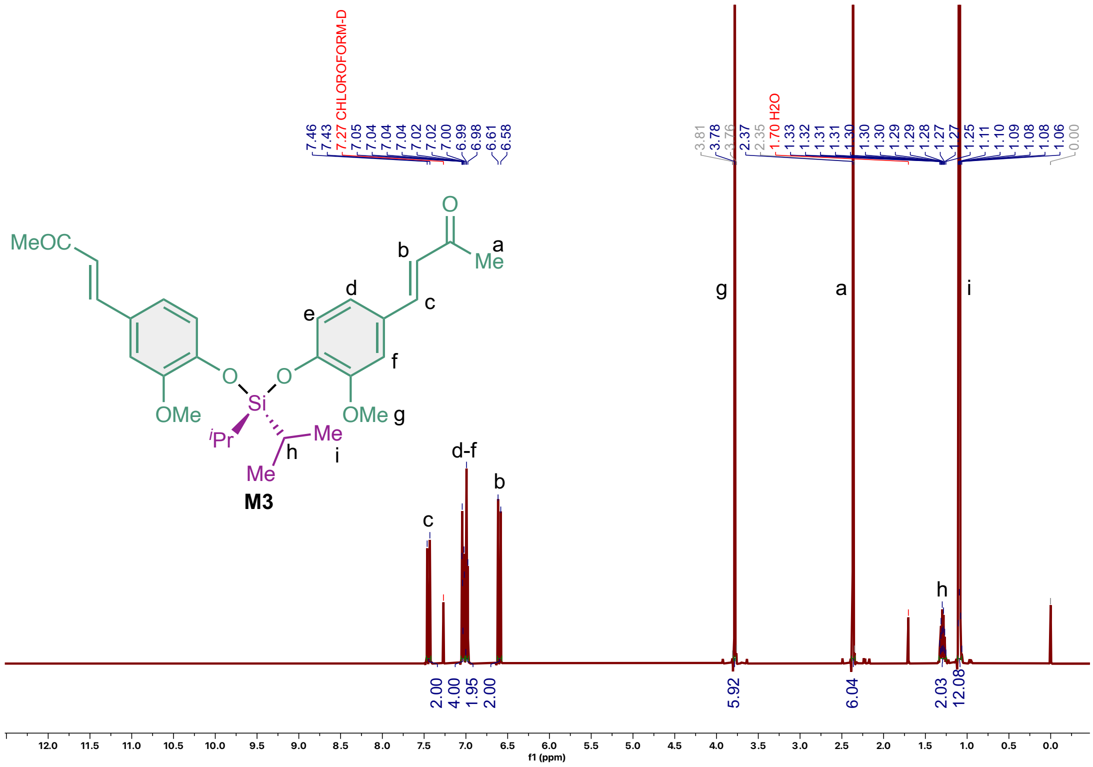

Figure S30. ^{1}H NMR spectrum of **M3** [500 MHz, CHLOROFORM-*D*].

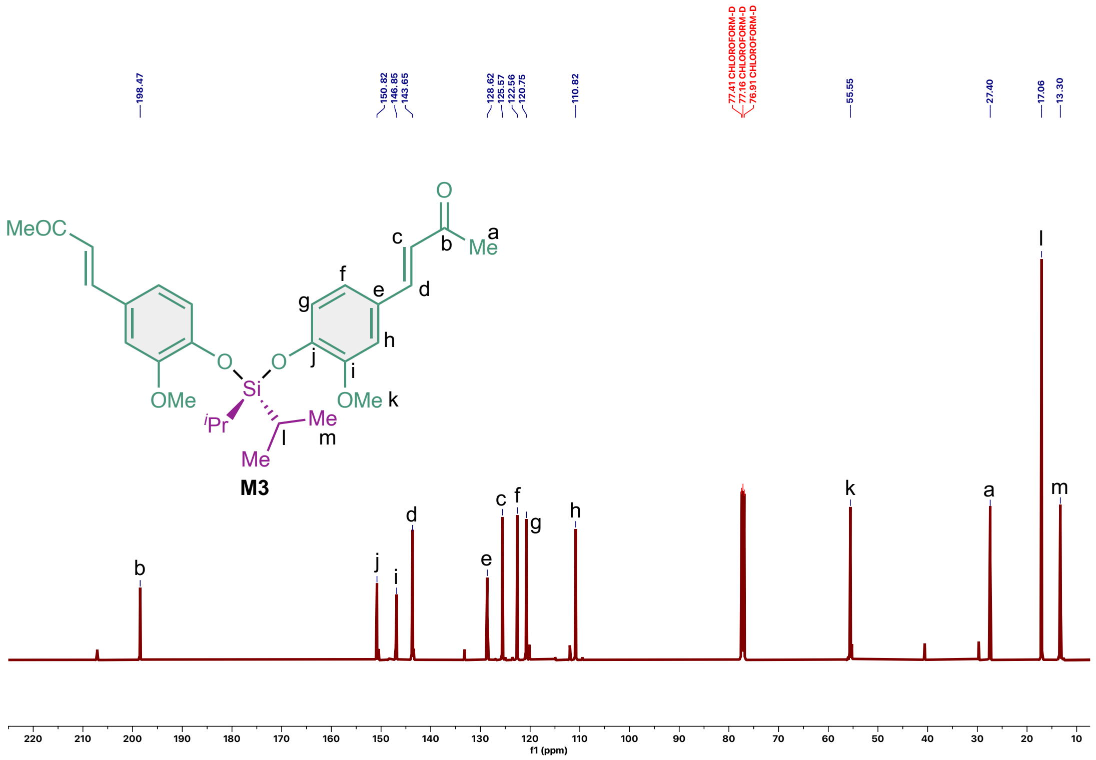

Figure S31. ^{13}C NMR spectrum of **M3** [126 MHz, CHLOROFORM-*D*].

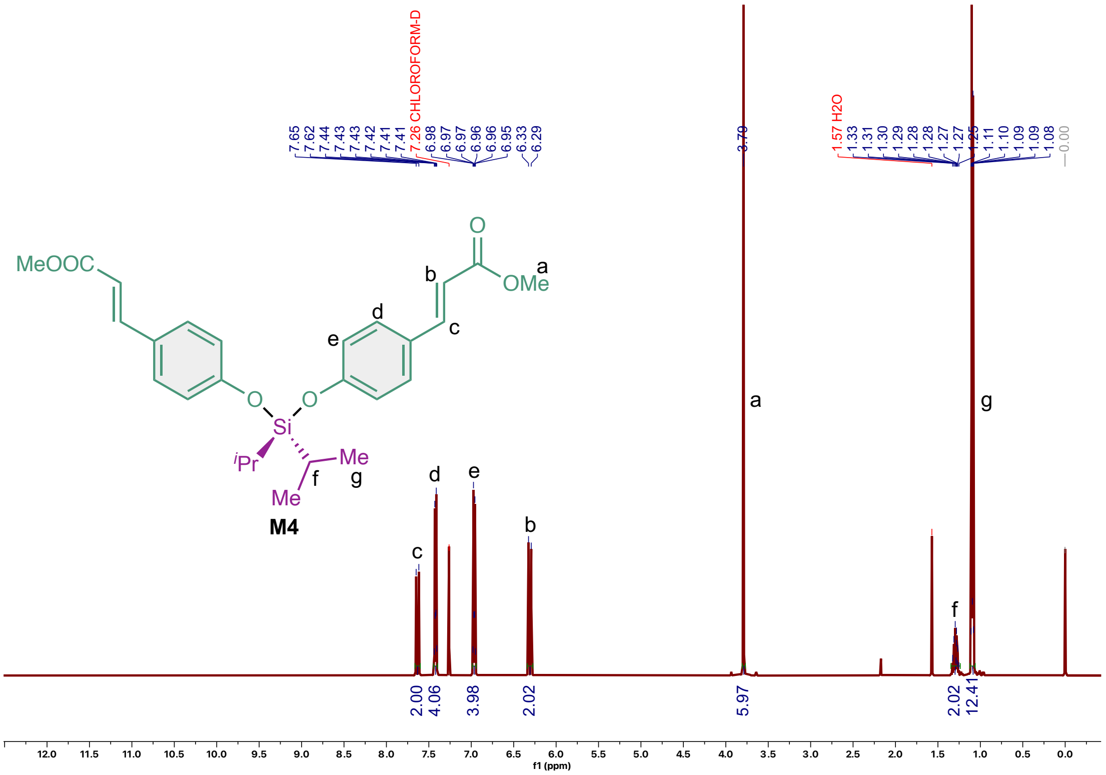

Figure S32. ^{1}H NMR spectrum of **M4** [500 MHz, CHLOROFORM-*D*].

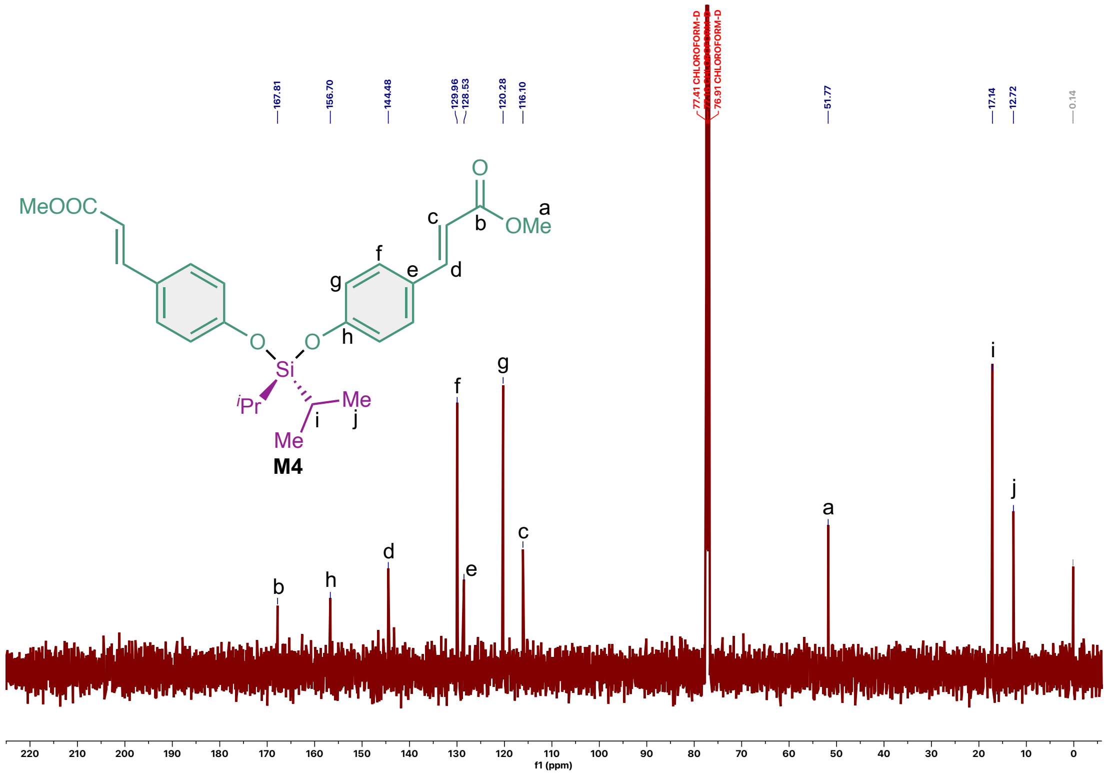

Figure S33. ^{13}C NMR spectrum of **M4** [126 MHz, CHLOROFORM-*D*].

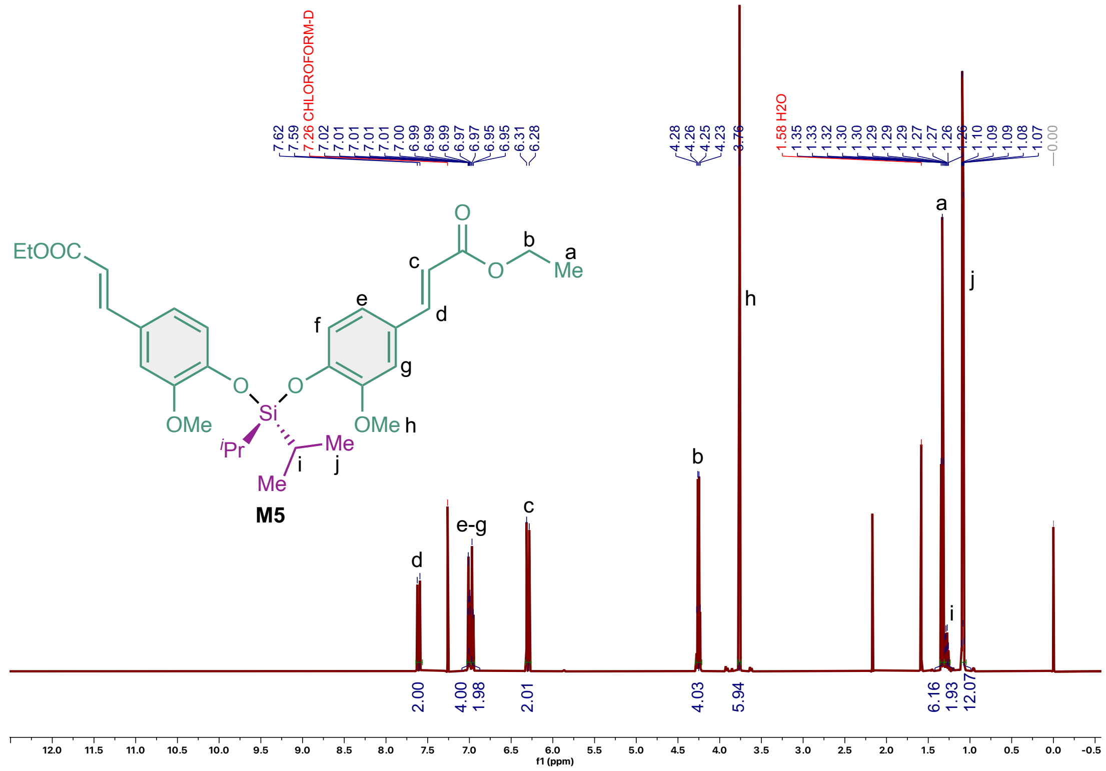

Figure S34. ^{1}H NMR spectrum of **M5** [500 MHz, CHLOROFORM-*D*].

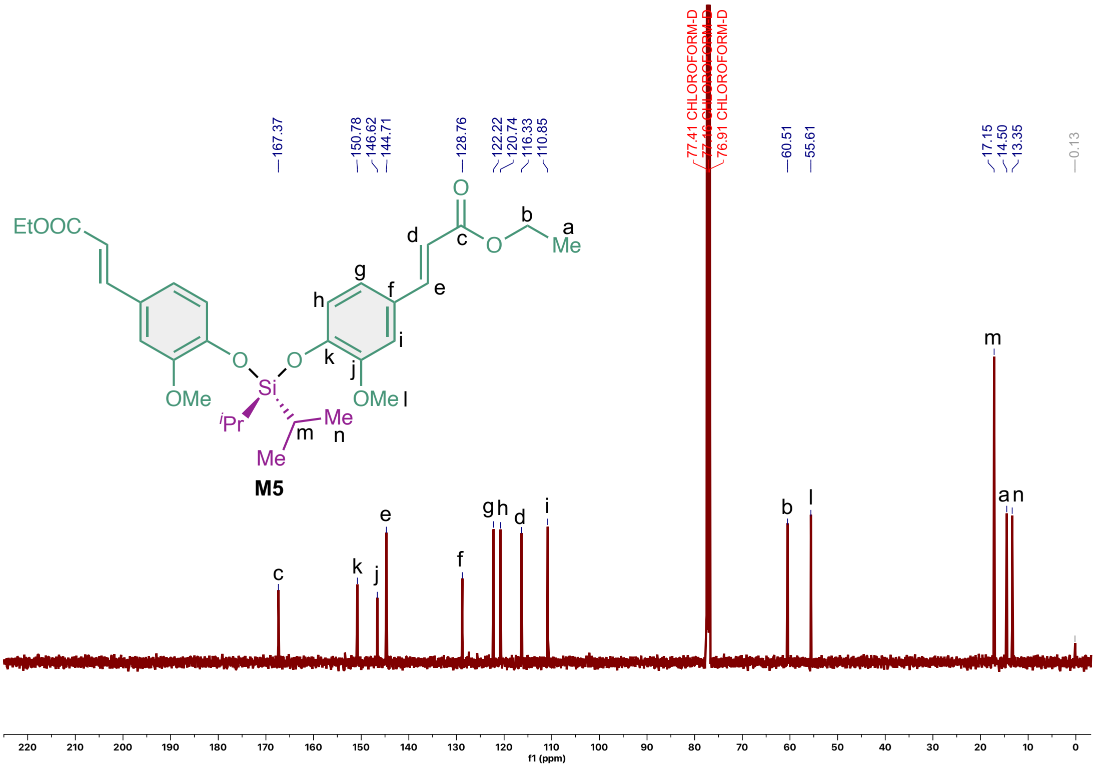

Figure S35. ^{13}C NMR spectrum of **M5** [126 MHz, CHLOROFORM-*D*].

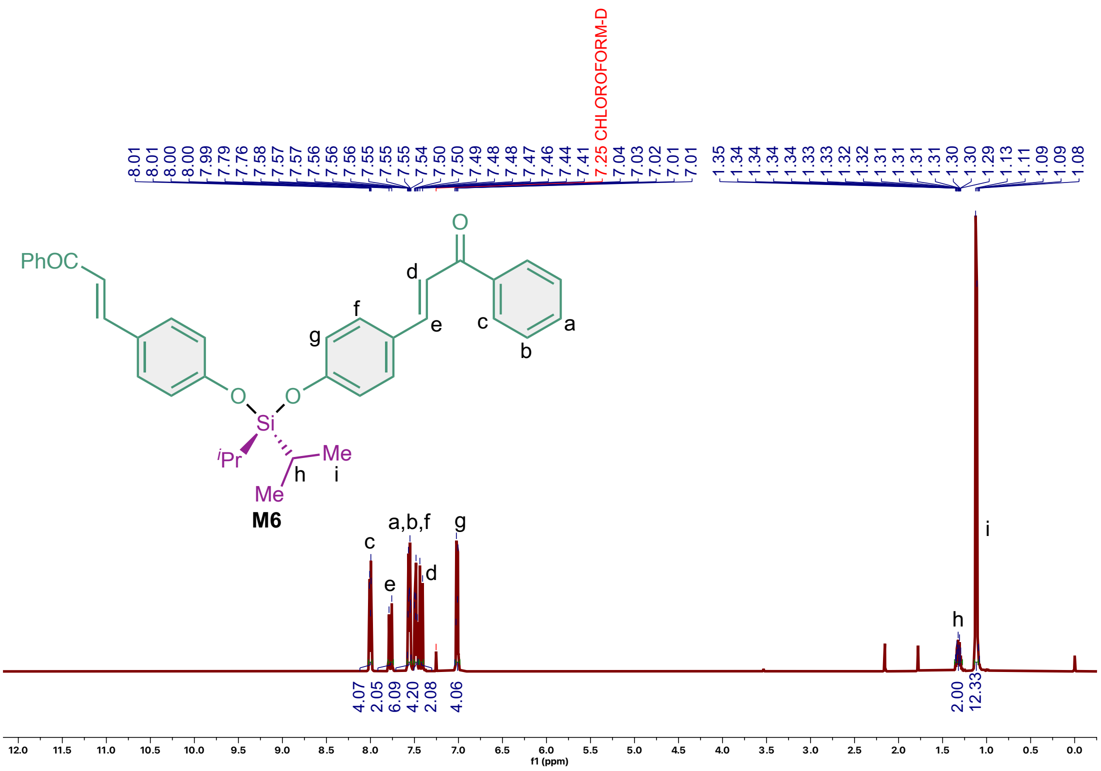

Figure S36. ^{1}H NMR spectrum of **M6** [500 MHz, CHLOROFORM-*D*].

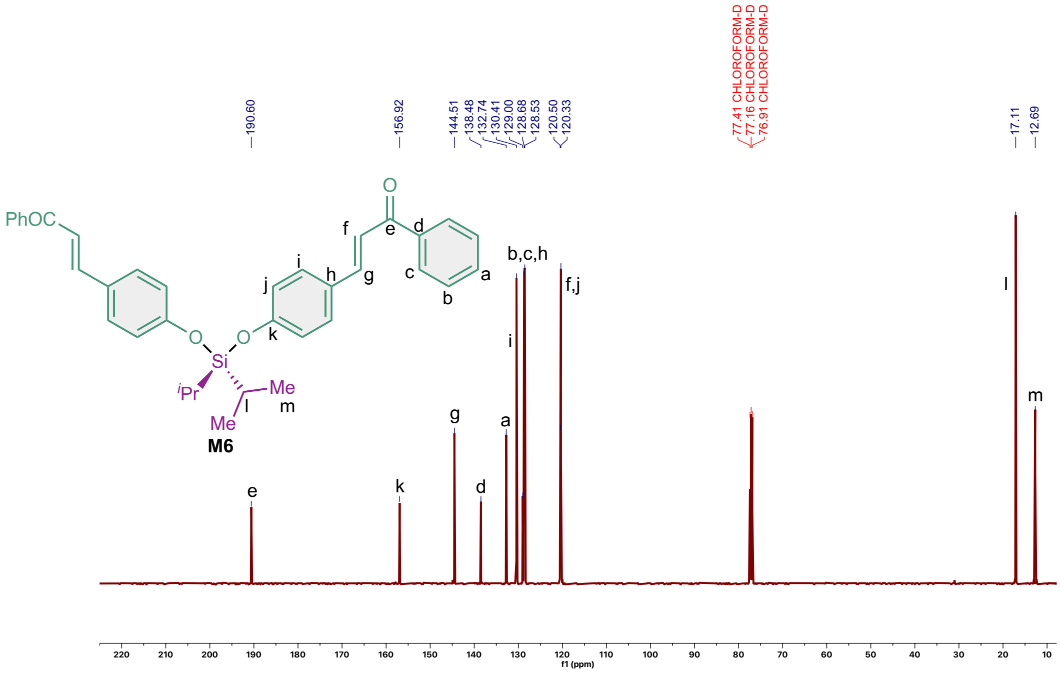

Figure S37. ^{13}C NMR spectrum of **M6** [126 MHz, CHLOROFORM-*D*].

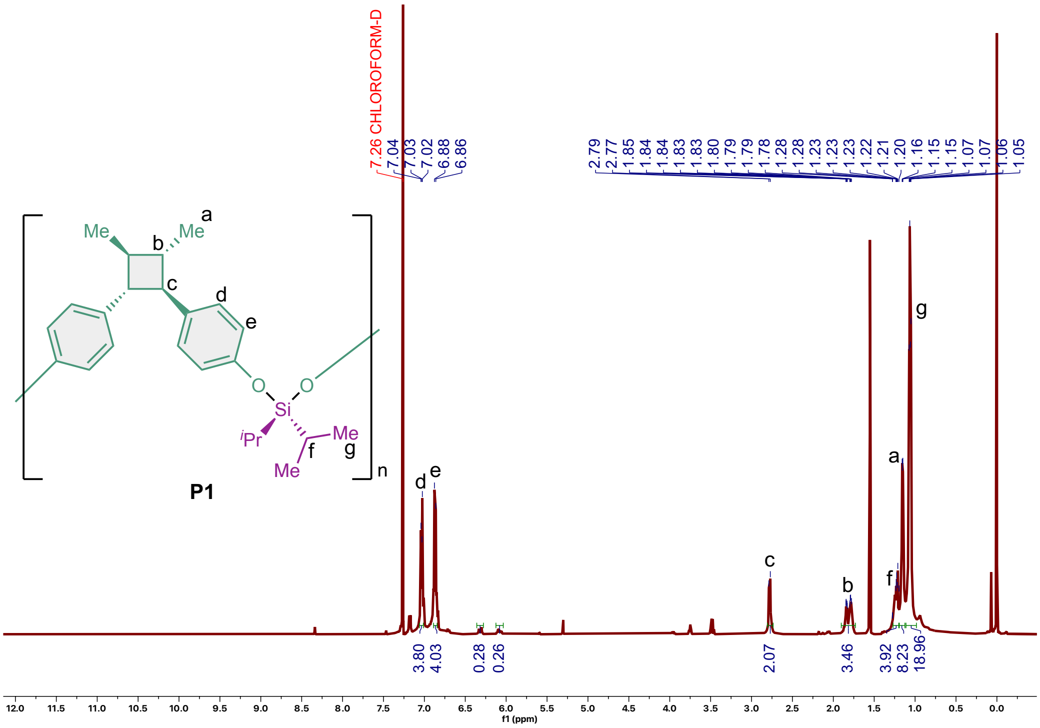

Figure S38. ^{1}H NMR spectrum of **P1** synthesized using PIDA [500 MHz, CHLOROFORM-*D*].

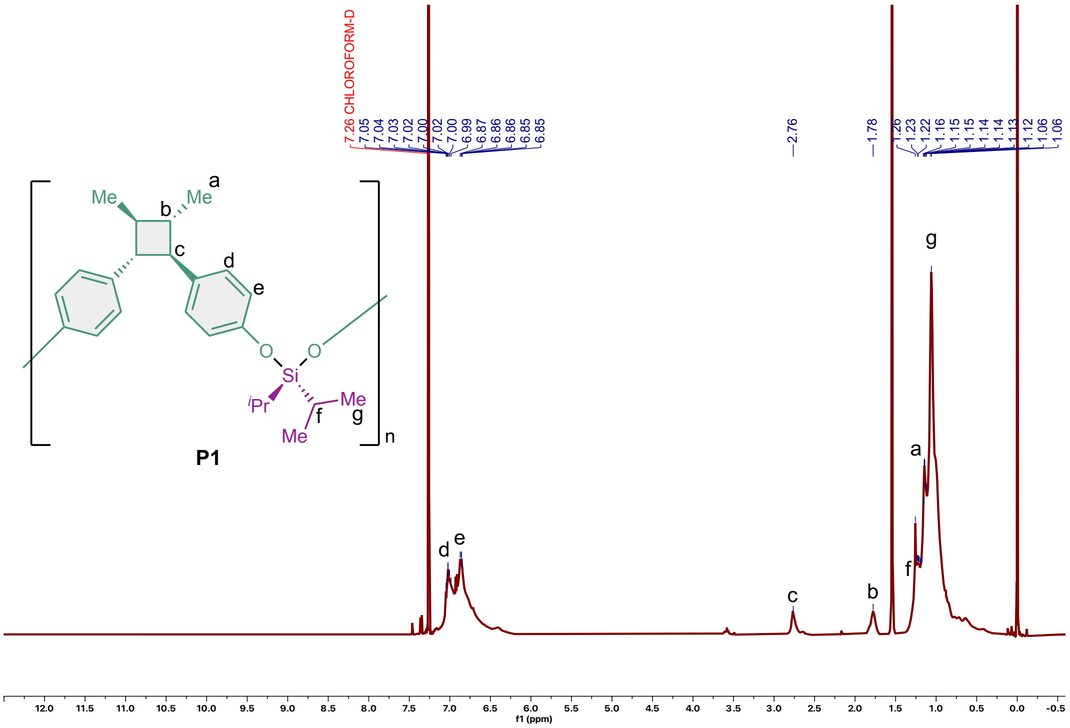

Figure S39. ^{1}H NMR spectrum of **P1** synthesized using magic blue [500 MHz, CHLOROFORM-*D*].

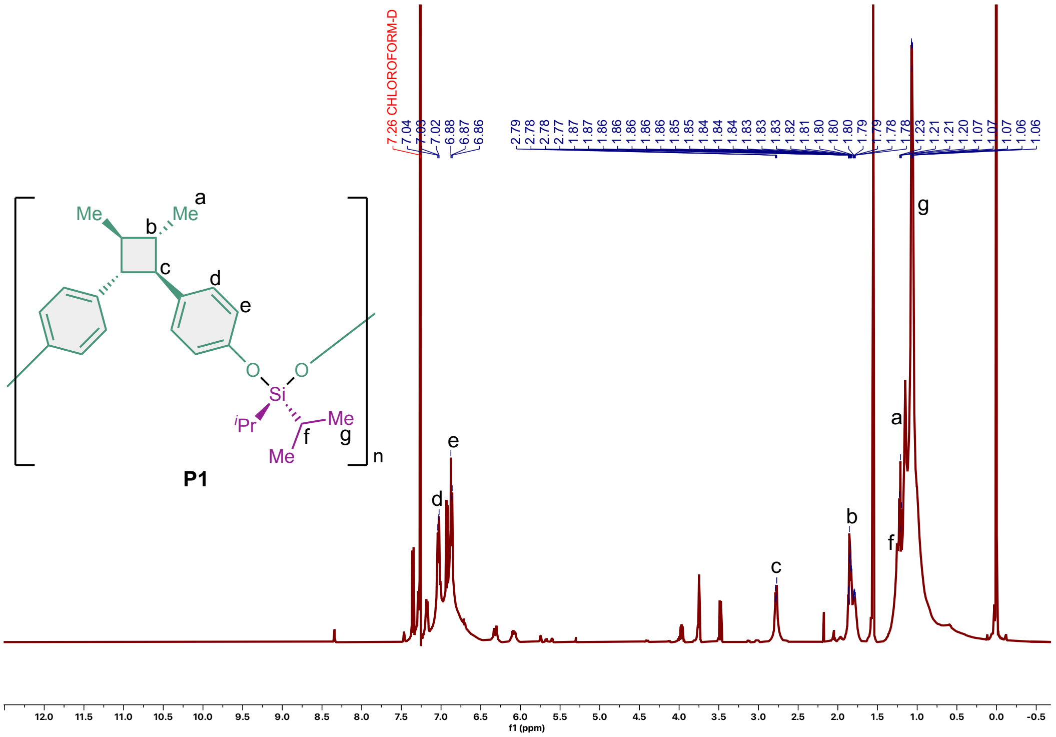

Figure S40. ^{1}H NMR spectrum of **P1** synthesized via electrolysis [500 MHz, CHLOROFORM-*D*].

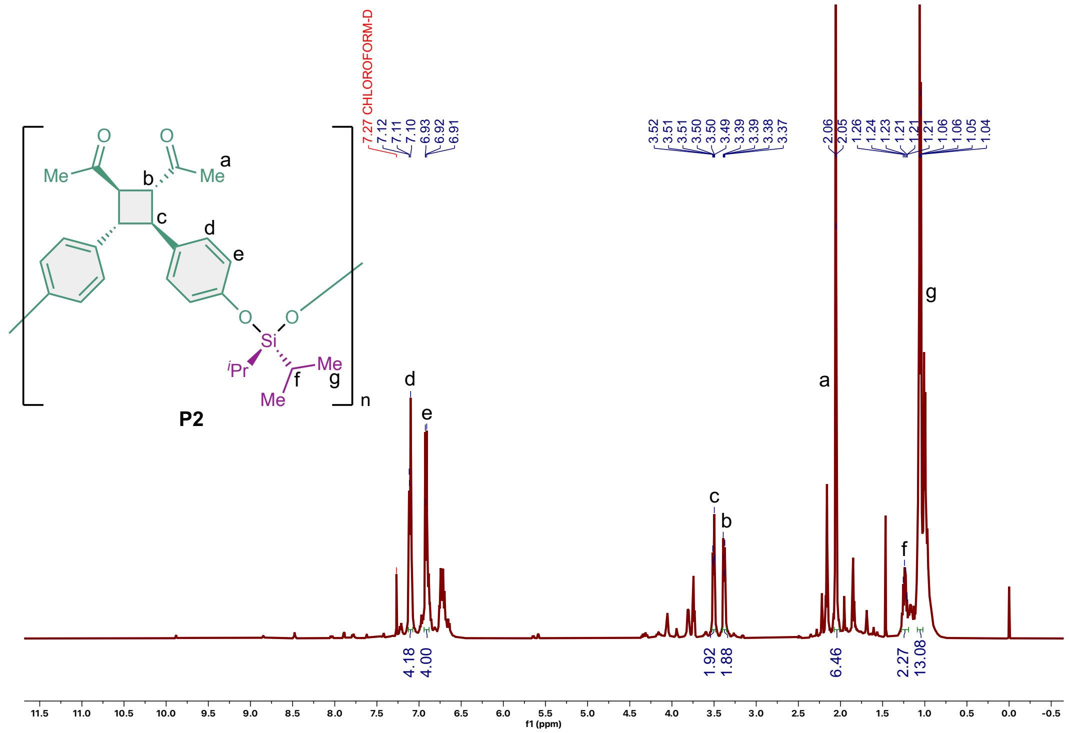

Figure S41. ^{1}H NMR spectrum of **P2** [500 MHz, CHLOROFORM-*D*].

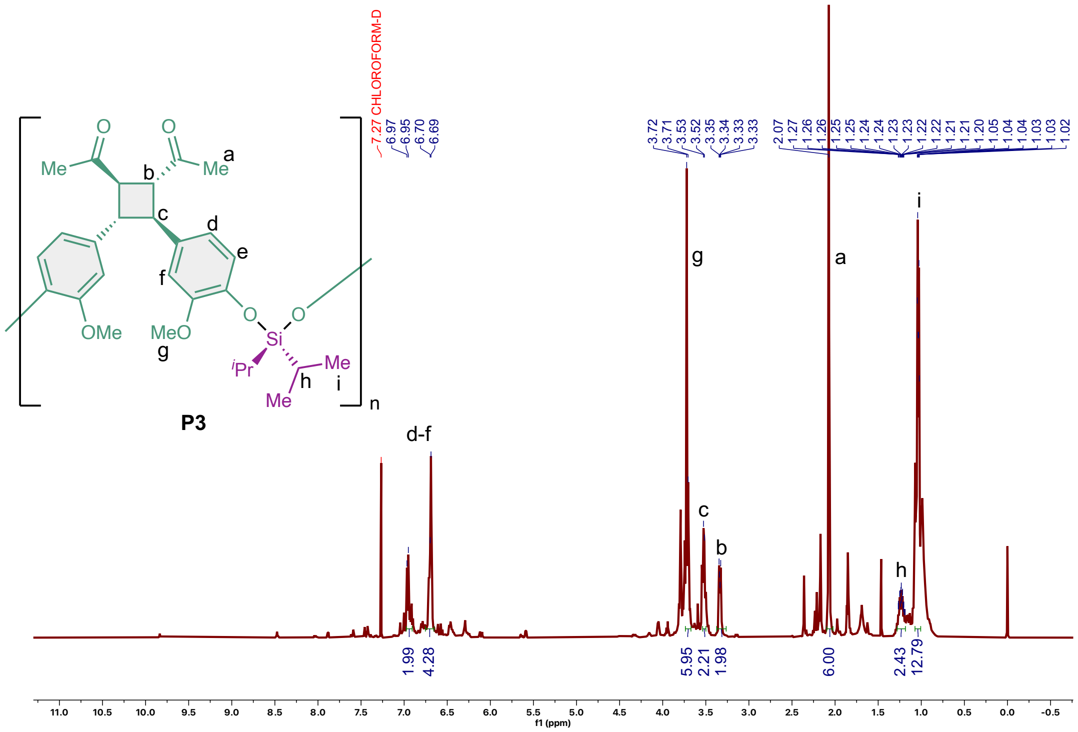

Figure S42. ^{1}H NMR spectrum of **P3** [500 MHz, CHLOROFORM-*D*].

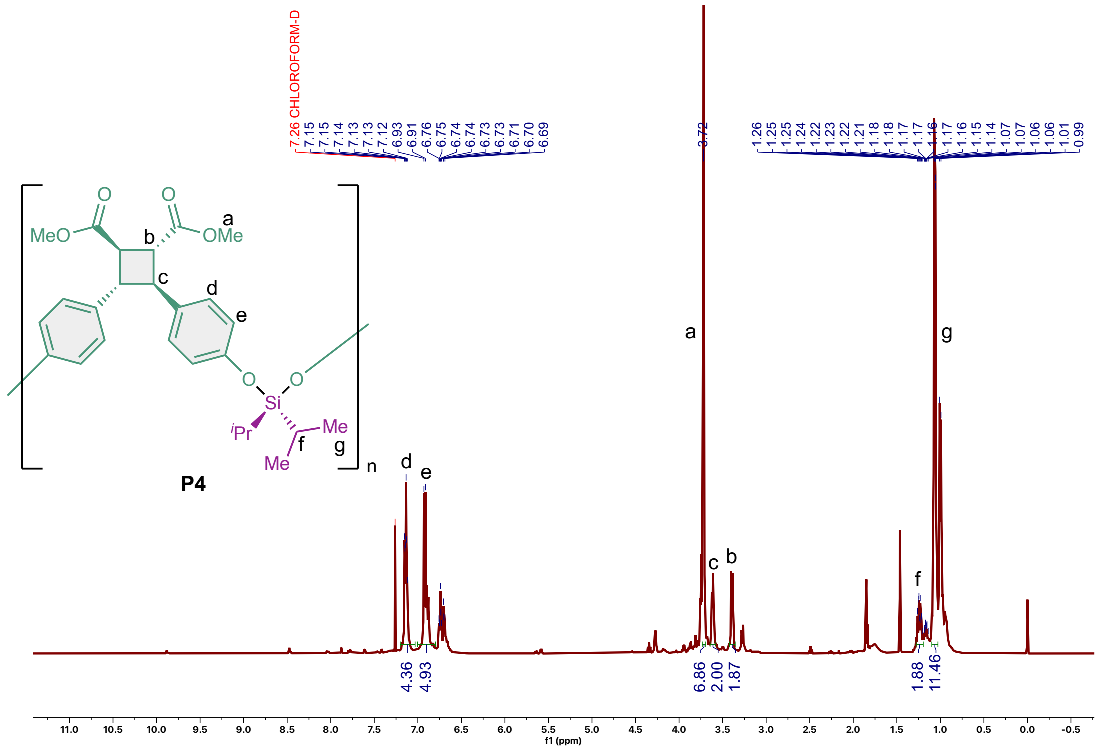

Figure S43. ^{1}H NMR spectrum of **P4** [500 MHz, CHLOROFORM-*D*].

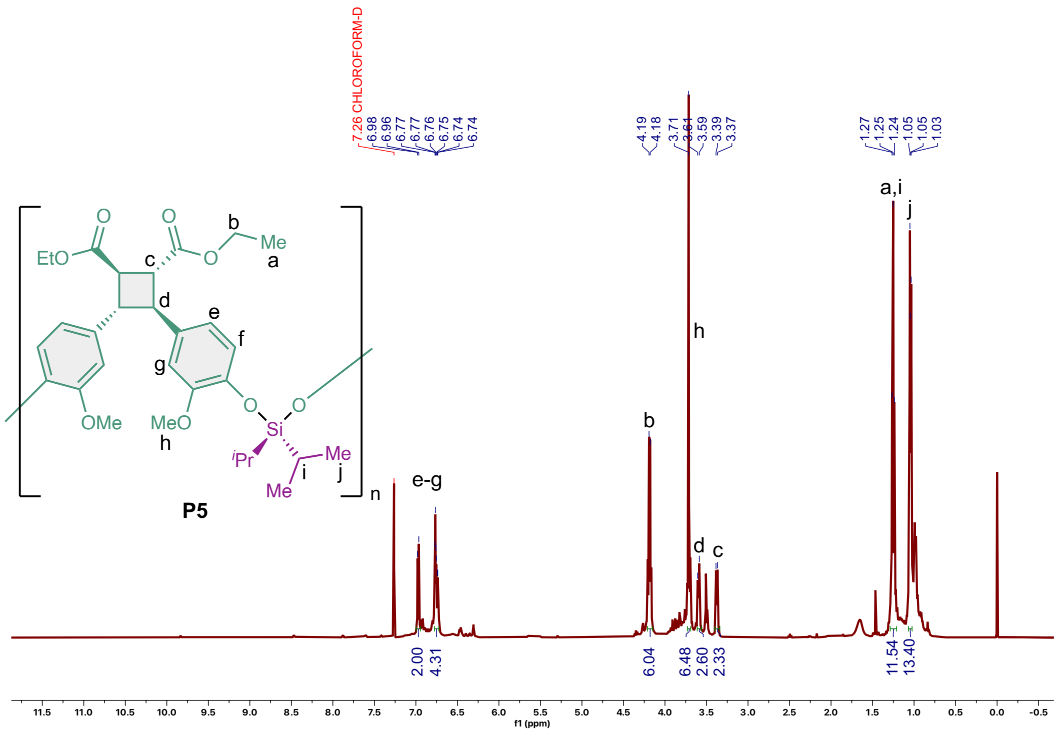

Figure S44. ^{1}H NMR spectrum of **P5** [500 MHz, CHLOROFORM-*D*].

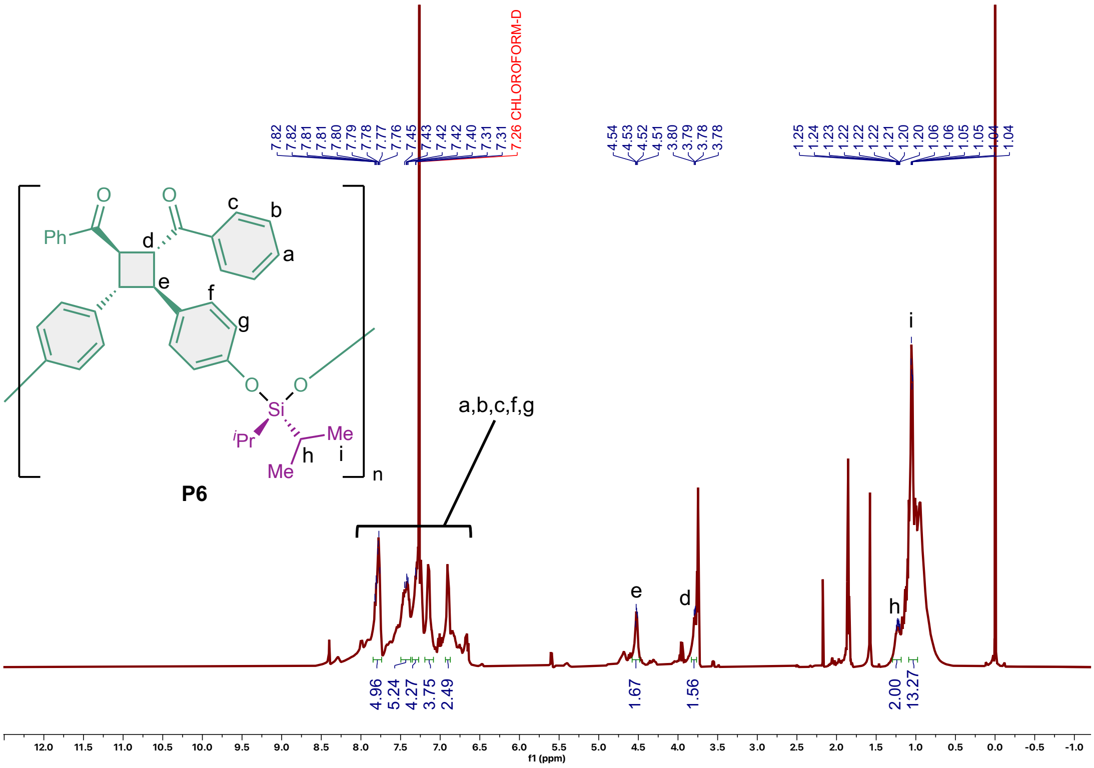

Figure S45. ^{1}H NMR spectrum of **P6** [500 MHz, CHLOROFORM-*D*].

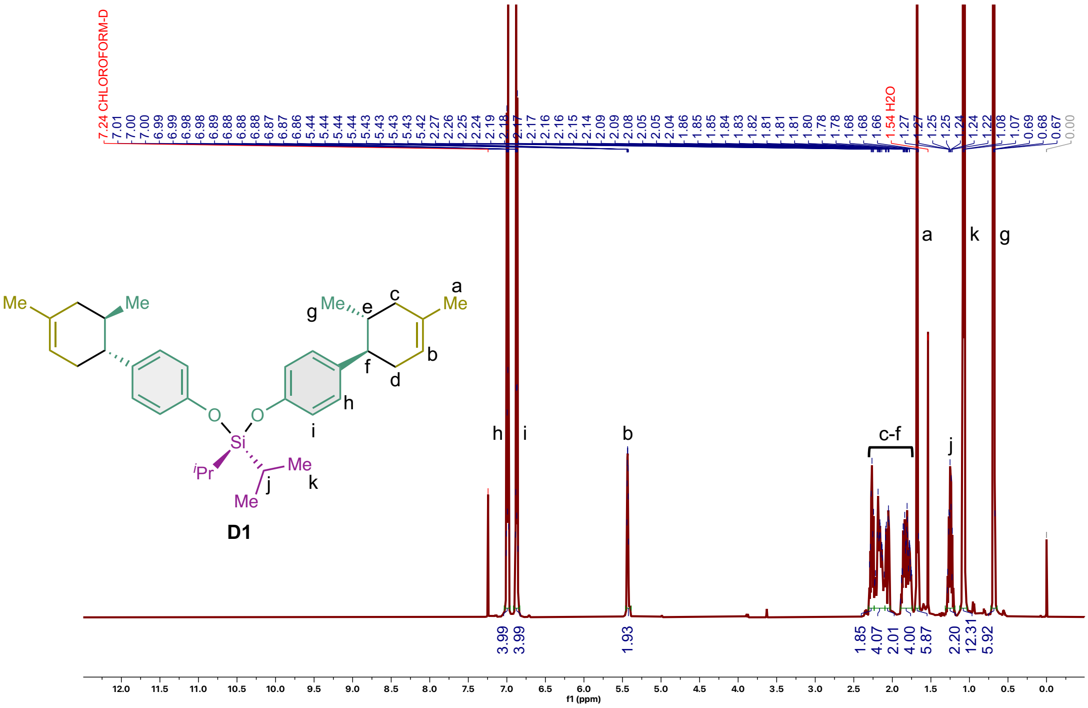

Figure S46. ^{1}H NMR spectrum of **D1** [500 MHz, CHLOROFORM-*D*].

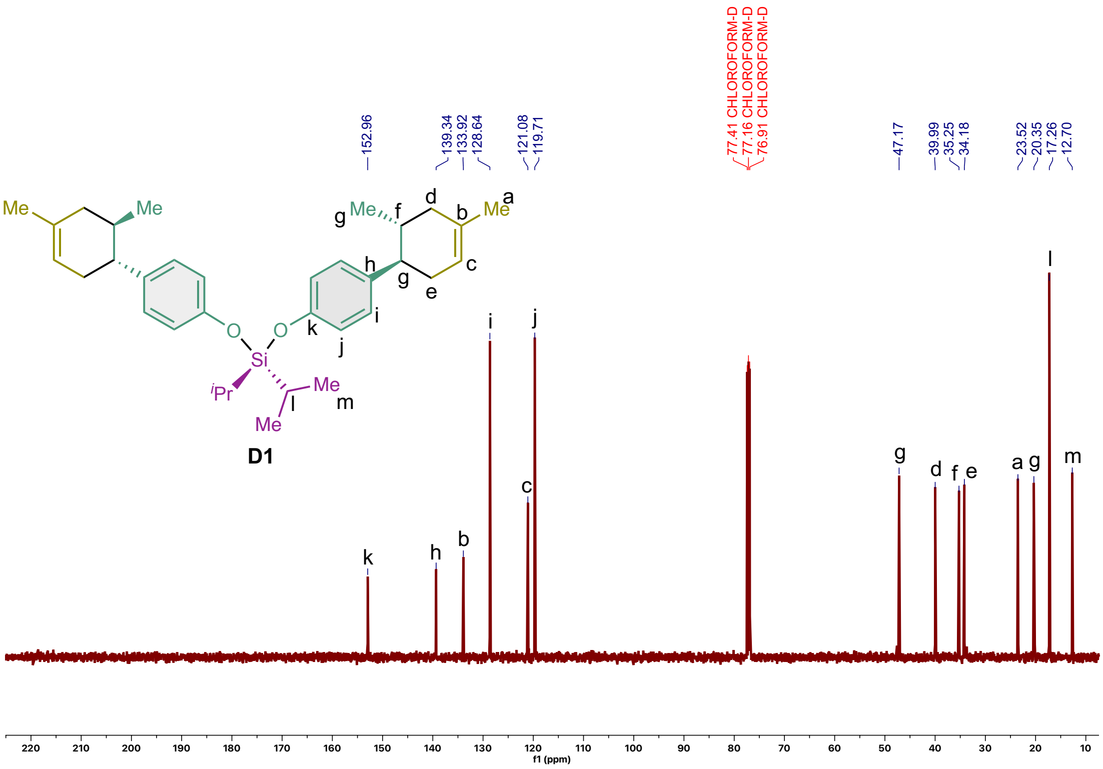

Figure S47. ^{13}C NMR spectrum of **D1** [126 MHz, CHLOROFORM-*D*].

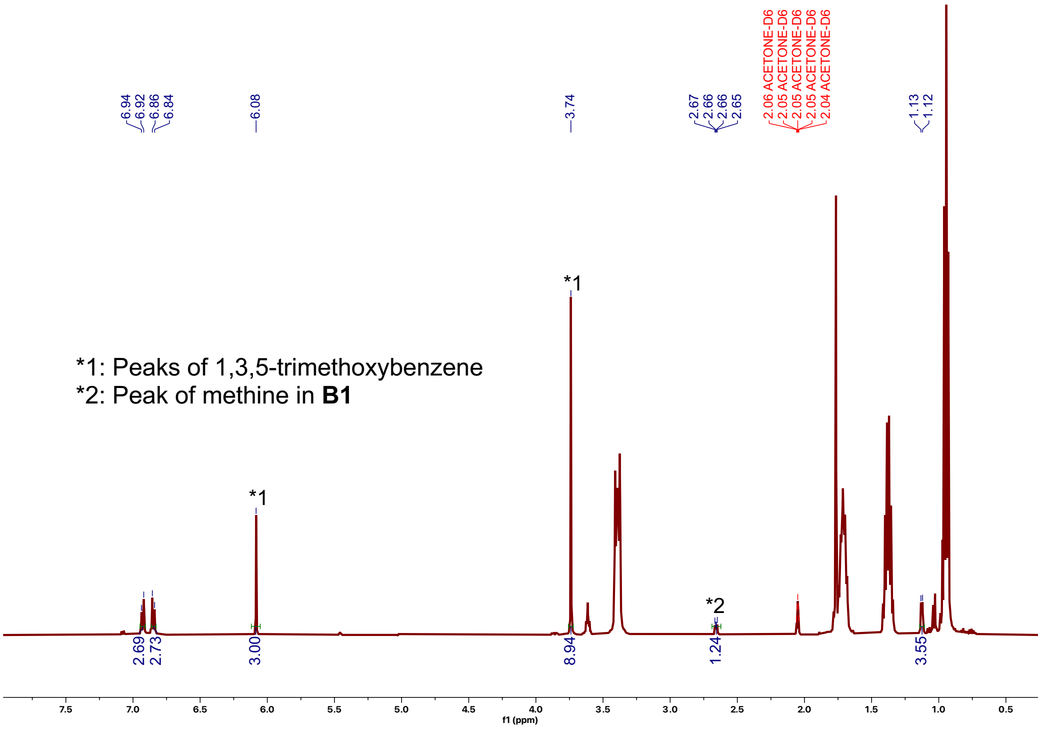

Figure S48. ^{1}H NMR spectrum after the decomposition of **P1** with 1,3,5-trimethoxybenzene as an internal standard. [500 MHz, ACETONE-D_6].

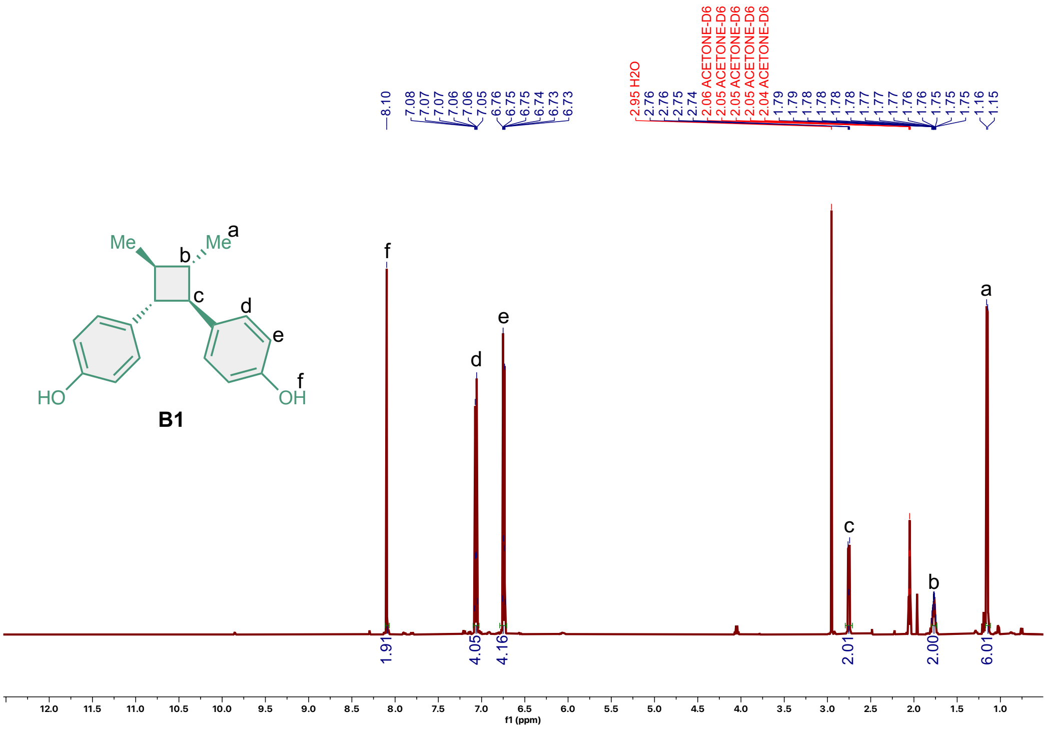

Figure S49. ^{1}H NMR spectrum of **B1** [500 MHz, ACETONE-D_6].

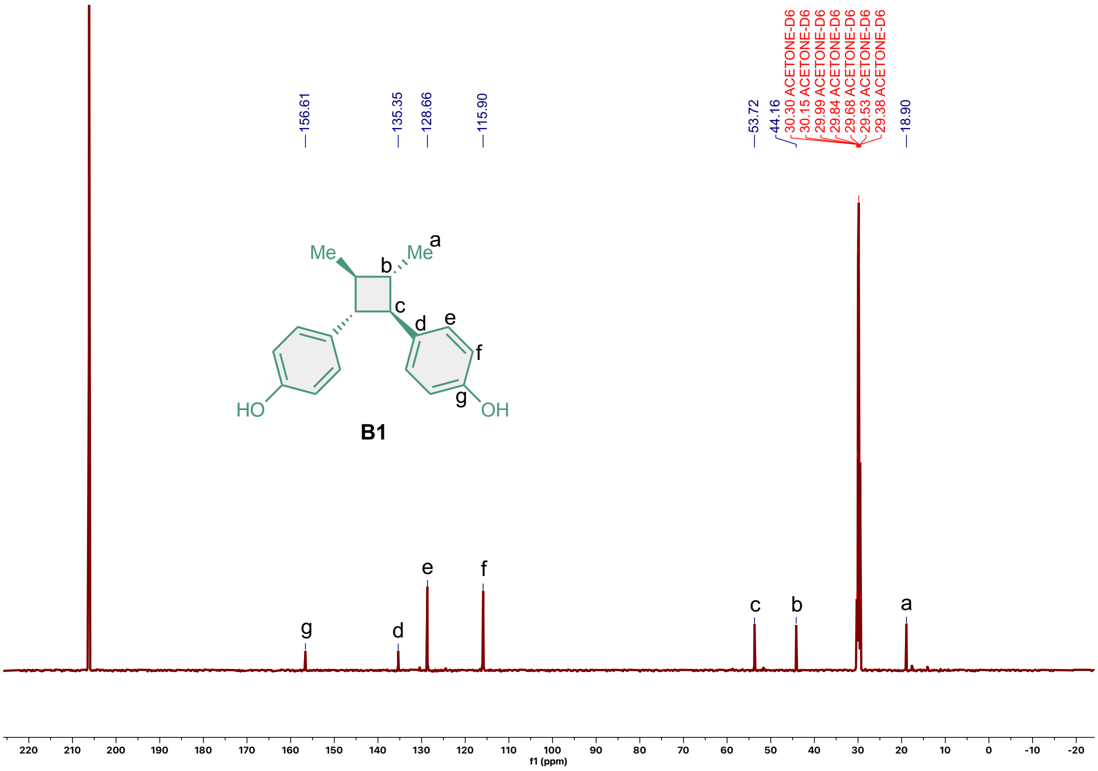

Figure S50. ^{13}C NMR spectrum of **B1** [126 MHz, ACETONE-D_6].

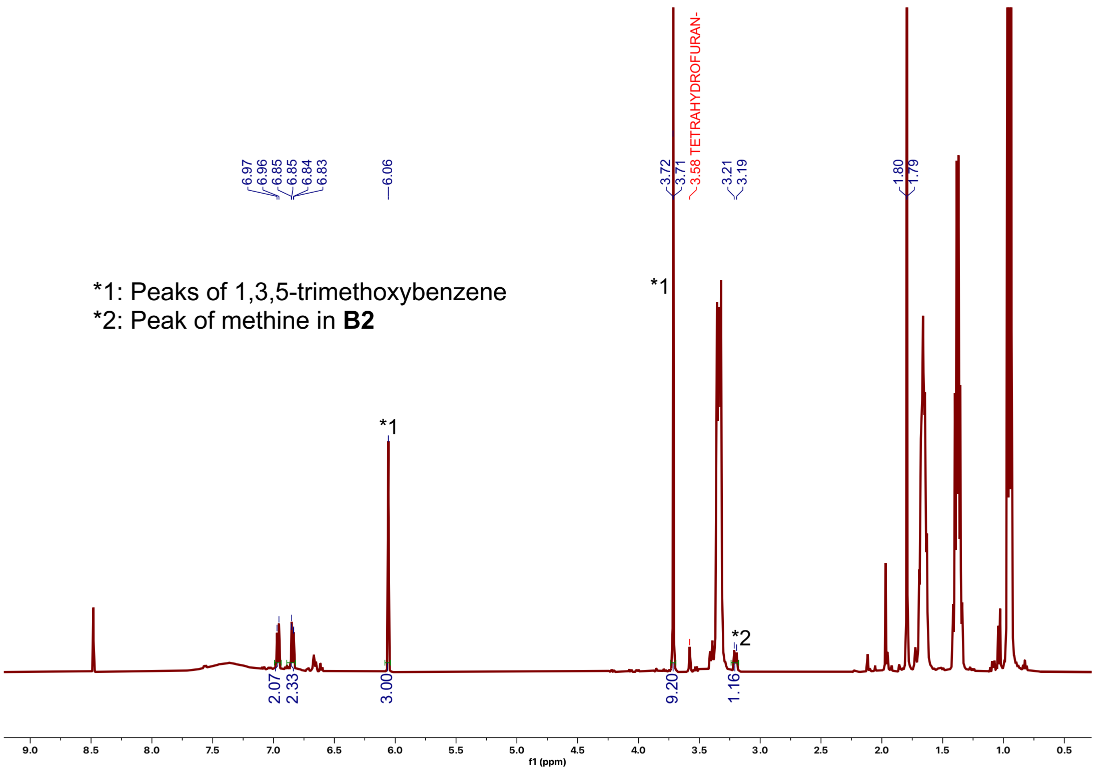

Figure S51. ^{1}H NMR spectrum after the decomposition of **P2** with 1,3,5-trimethoxybenzene as an internal standard. [500 MHz, THF-D_8].

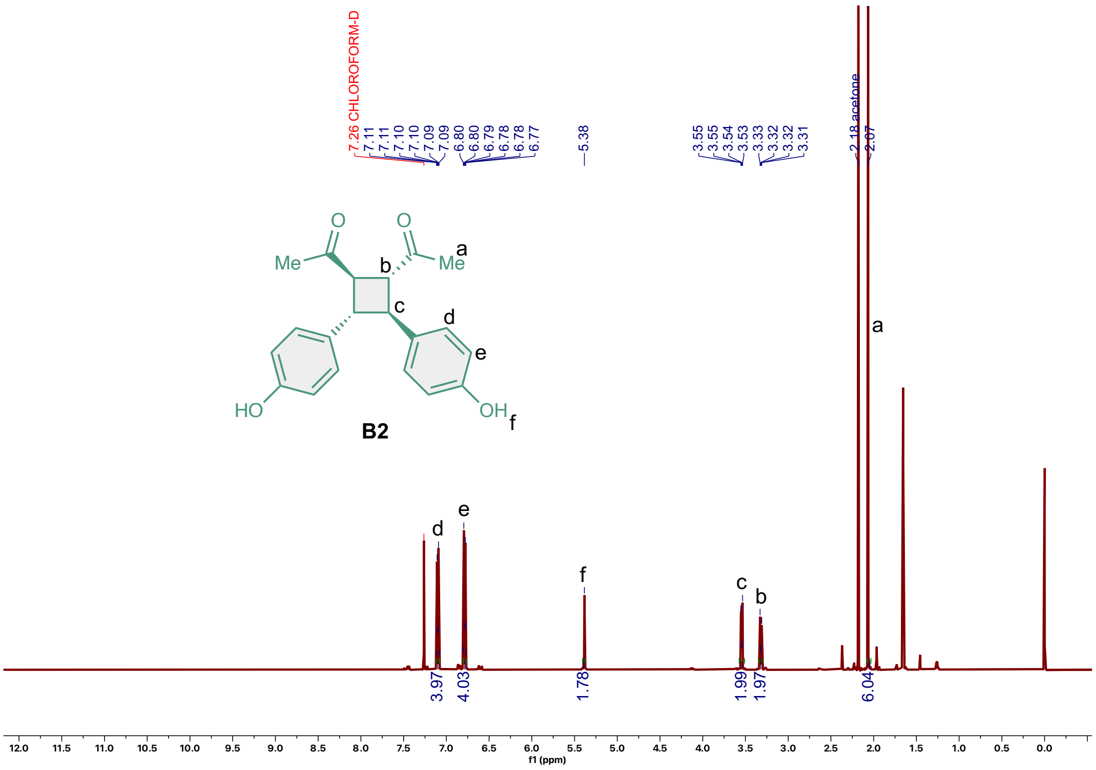

Figure S52. ^{1}H NMR spectrum of **B2** [500 MHz, CHLOROFORM-*D*].

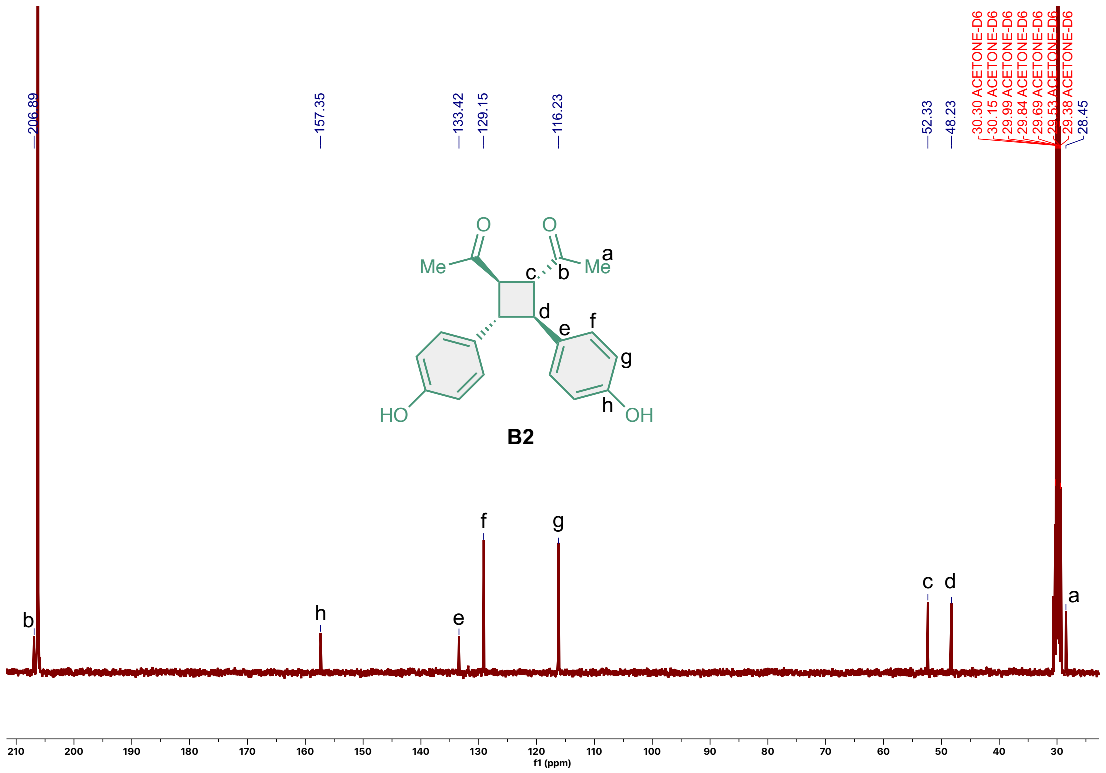

Figure S53. ^{13}C NMR spectrum of **B2** [126 MHz, ACETONE-D_6].

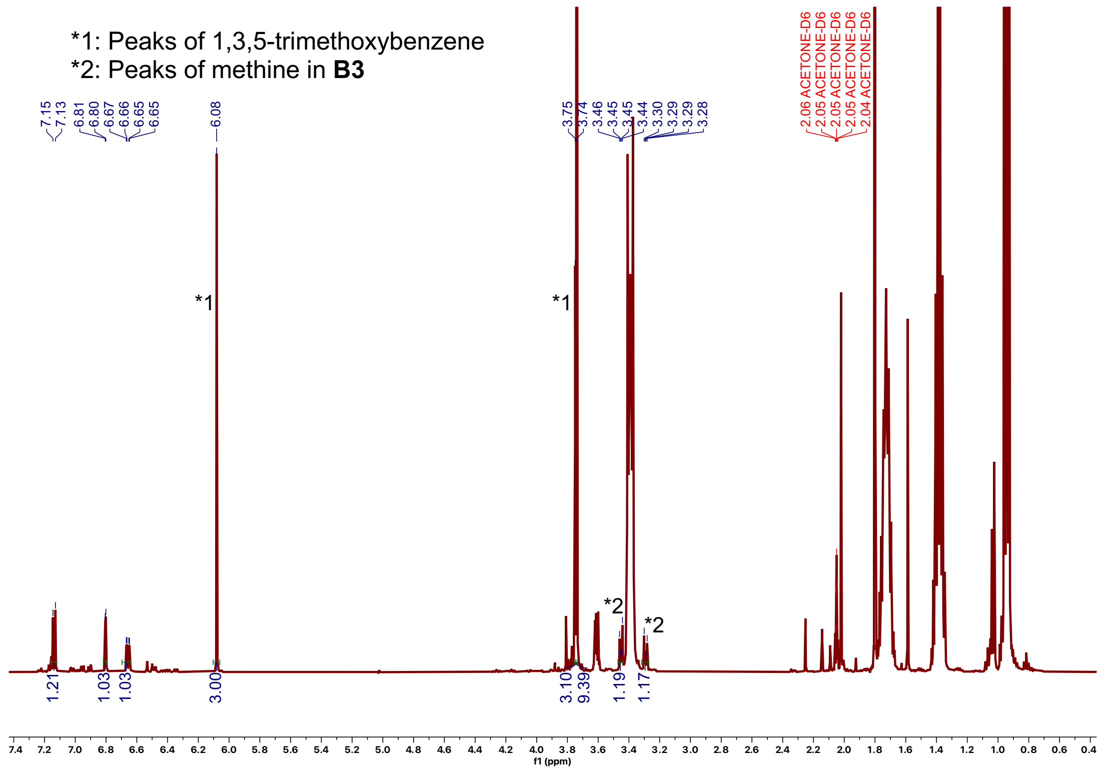

Figure S54. ^{1}H NMR spectrum after the decomposition of **P3** with 1,3,5-trimethoxybenzene as an internal standard. [500 MHz, ACETONE-D_6].

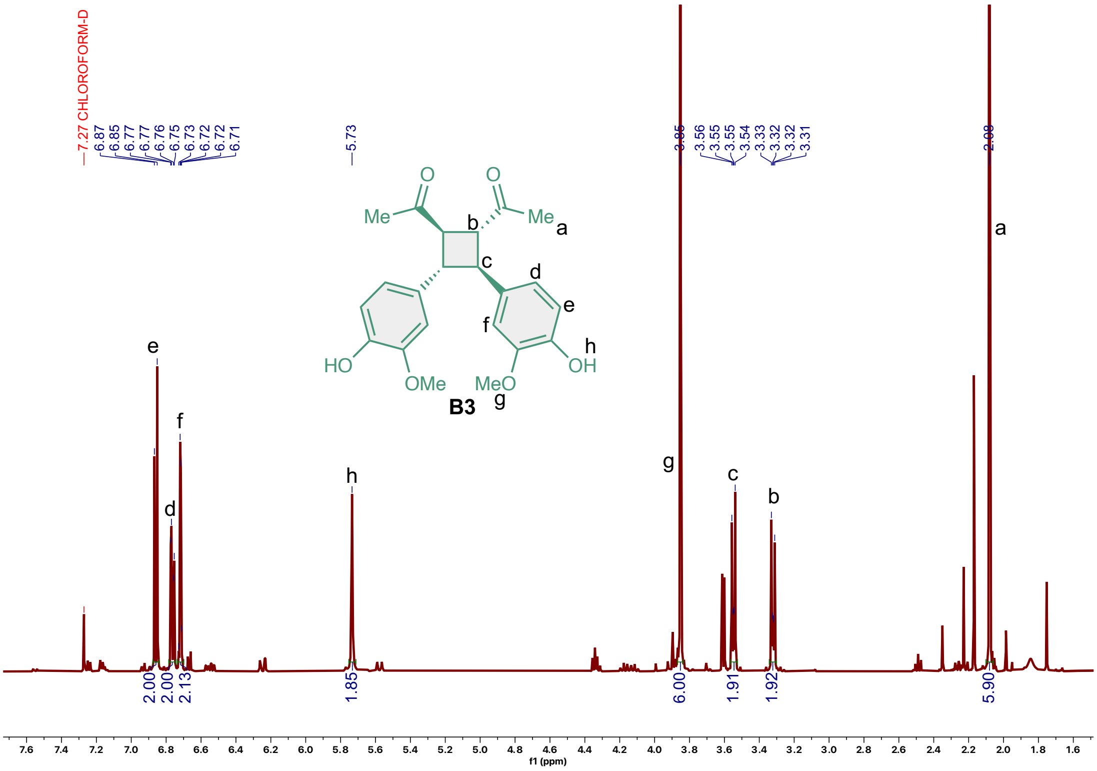

Figure S55. ^{1}H NMR spectrum of **B3** [500 MHz, CHLOROFORM-*D*].

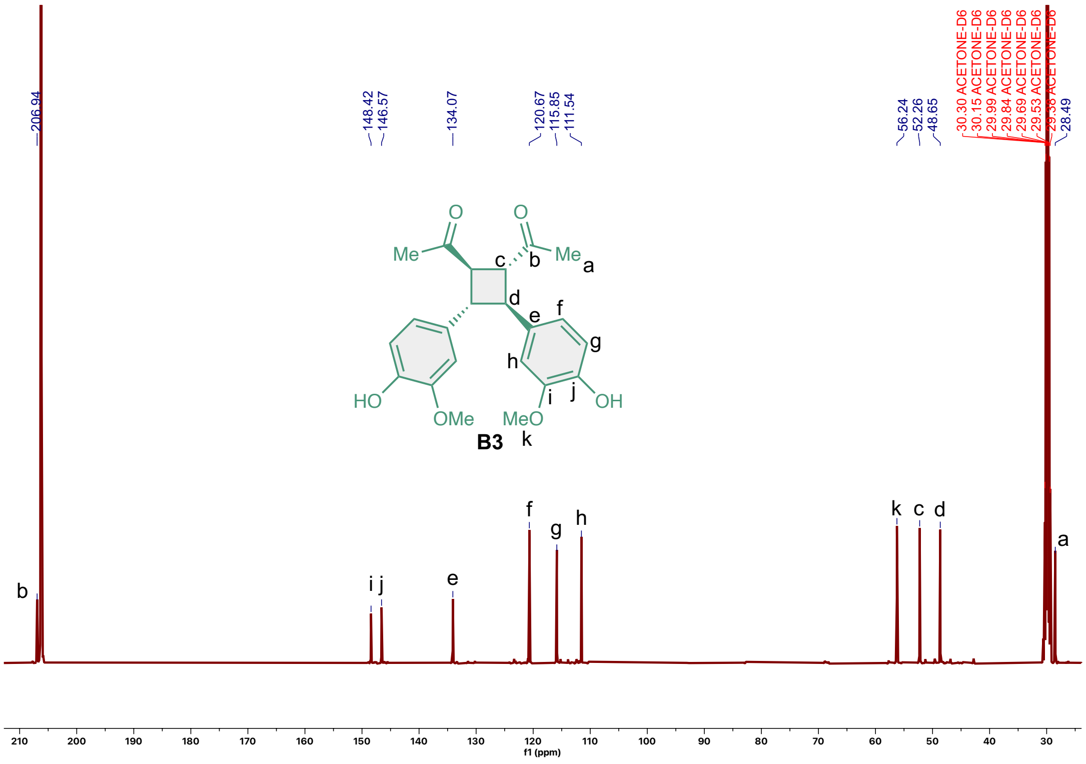

Figure S56. ^{13}C NMR spectrum of **B3** [126 MHz, ACETONE-D_6].

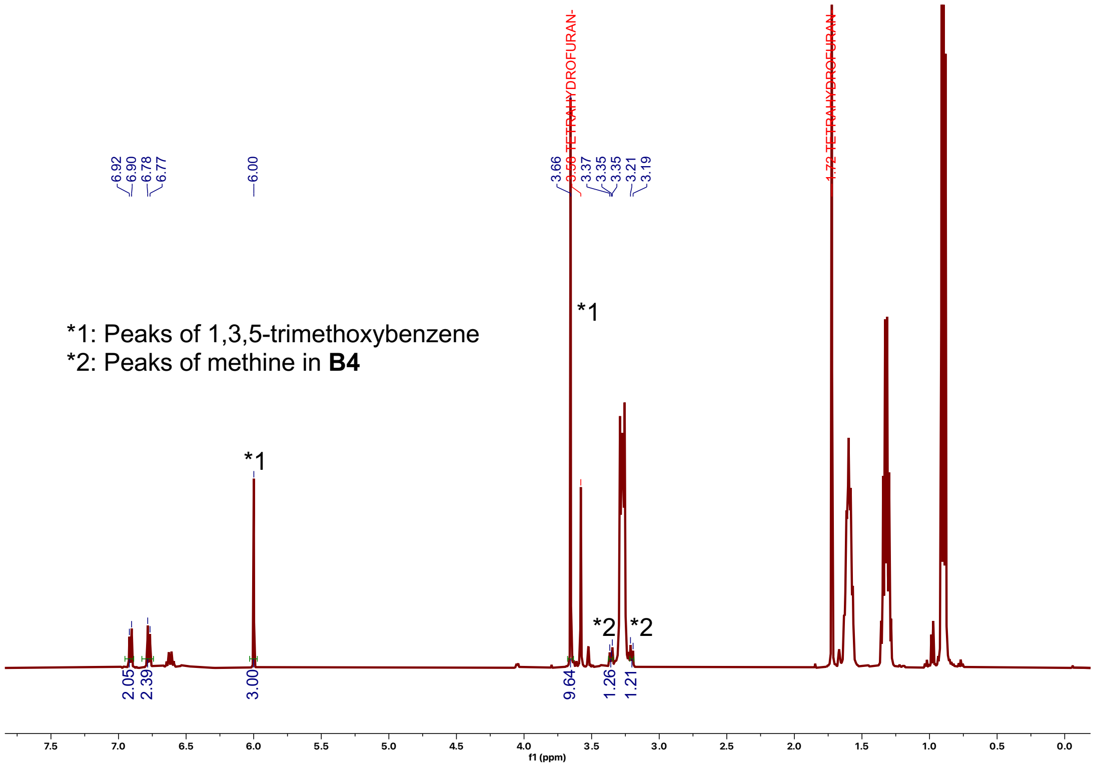

Figure S57. ^{1}H NMR spectrum after the decomposition of **P4** with 1,3,5-trimethoxybenzene as an internal standard. [500 MHz, THF-D_8].

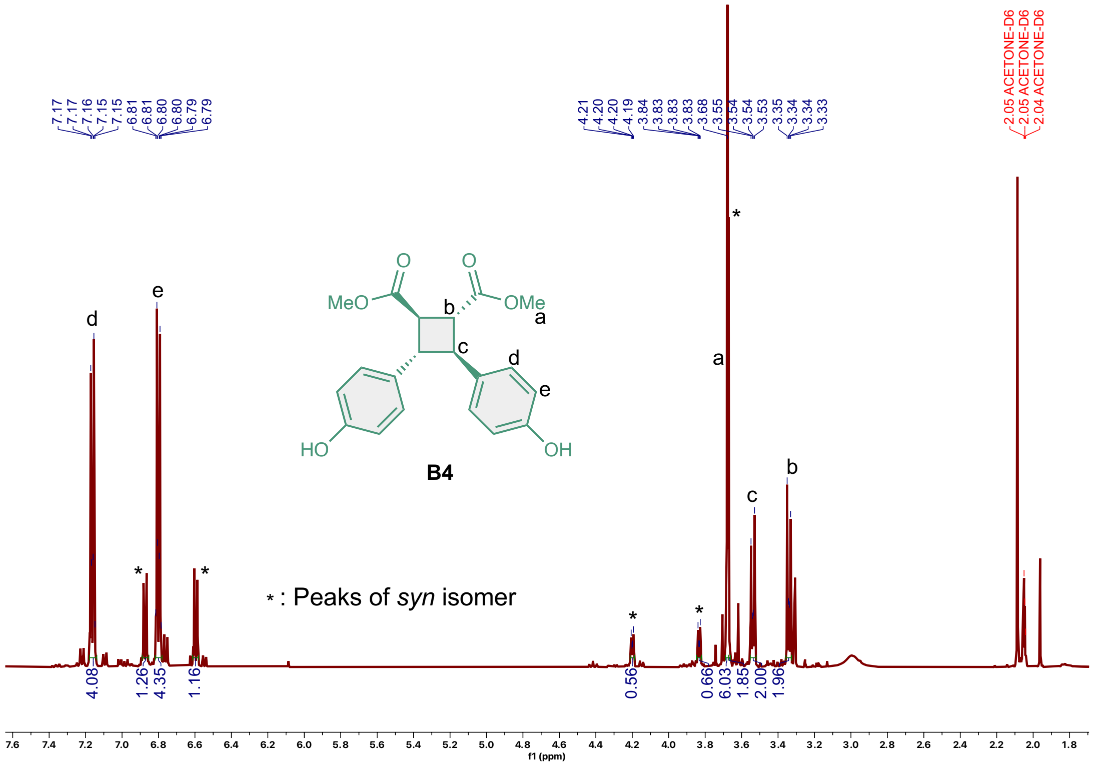

Figure S58. ^{1}H NMR spectrum of **B4** [500 MHz, ACETONE-D_6].

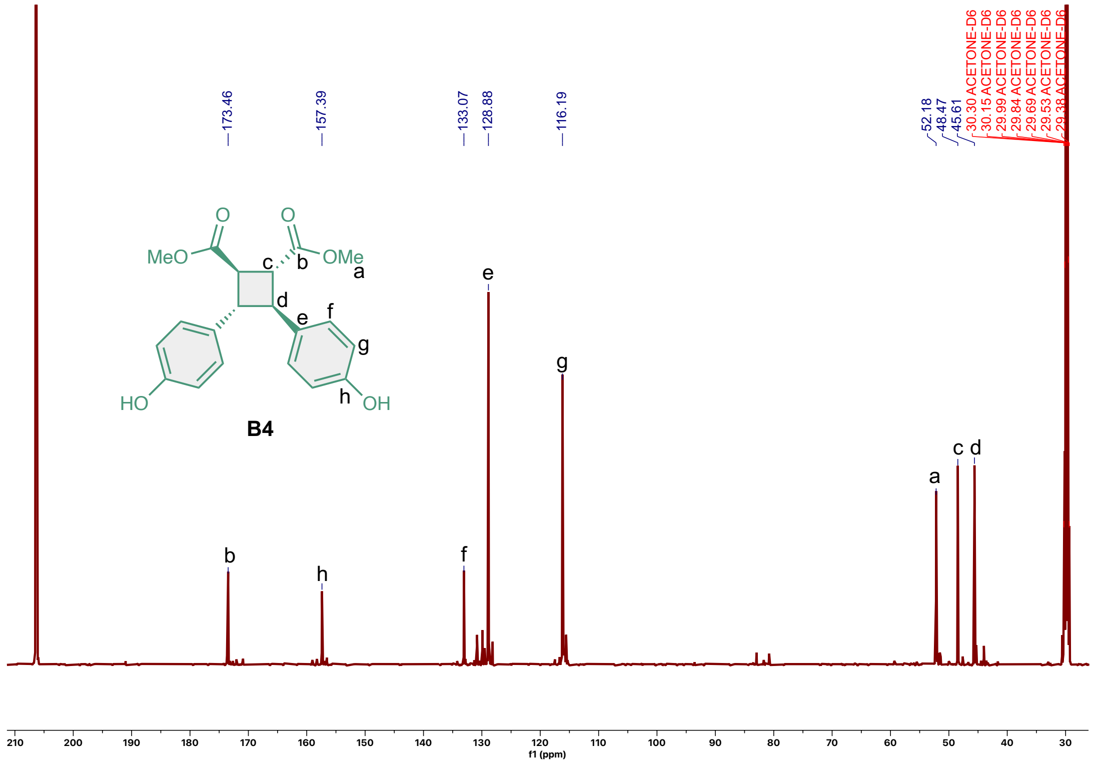

Figure S59. ^{13}C NMR spectrum of **B4** [126 MHz, ACETONE-D_6].

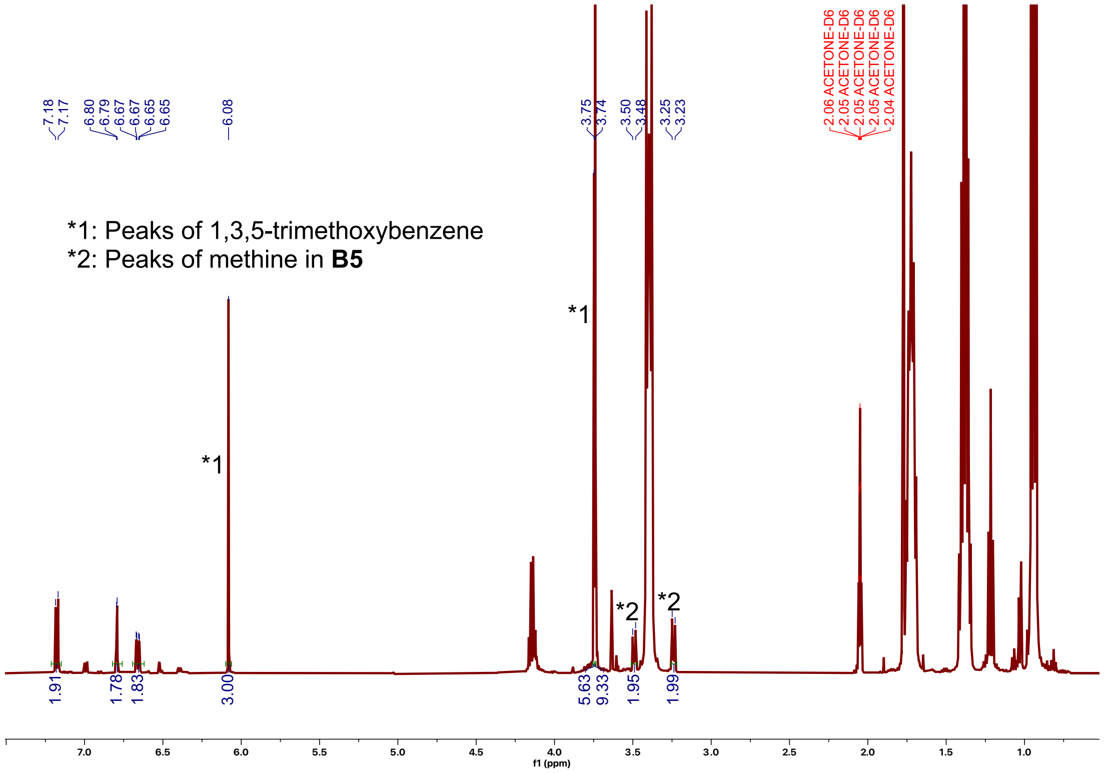

Figure S60. ^{1}H NMR spectrum after the decomposition of **P5** with 1,3,5-trimethoxybenzene as an internal standard. [500 MHz, ACETONE-D_6].

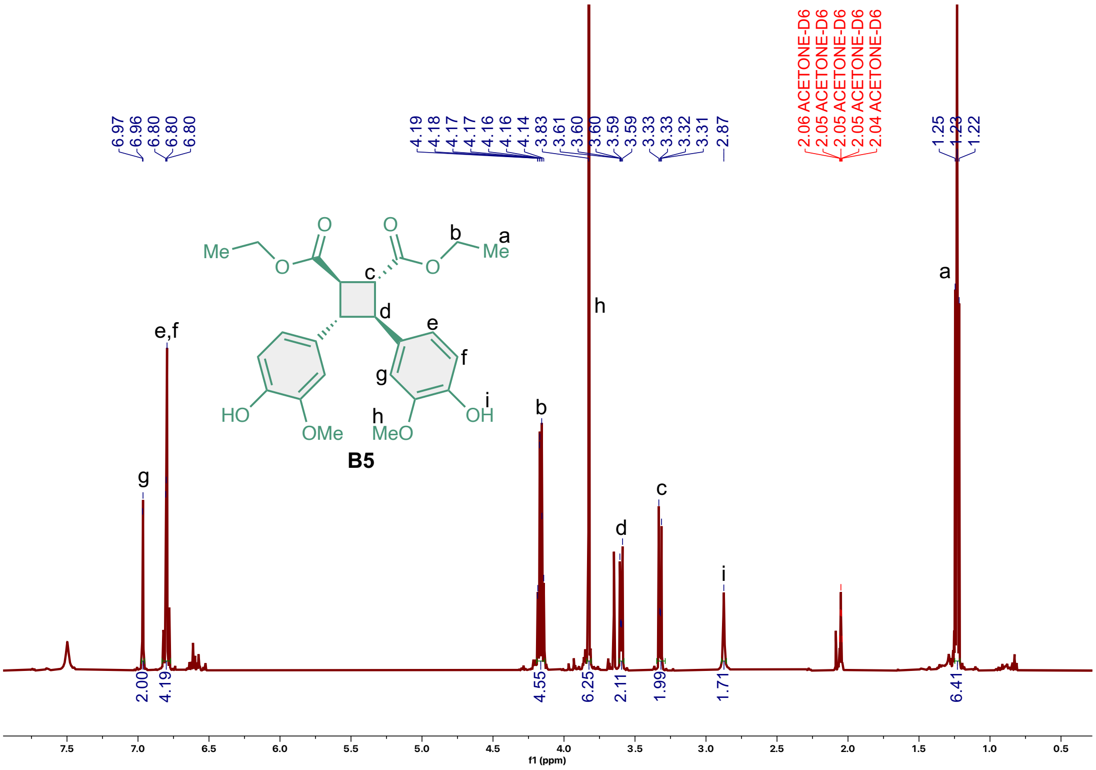

Figure S61. ^{1}H NMR spectrum of **B5** [500 MHz, ACETONE-D_6].

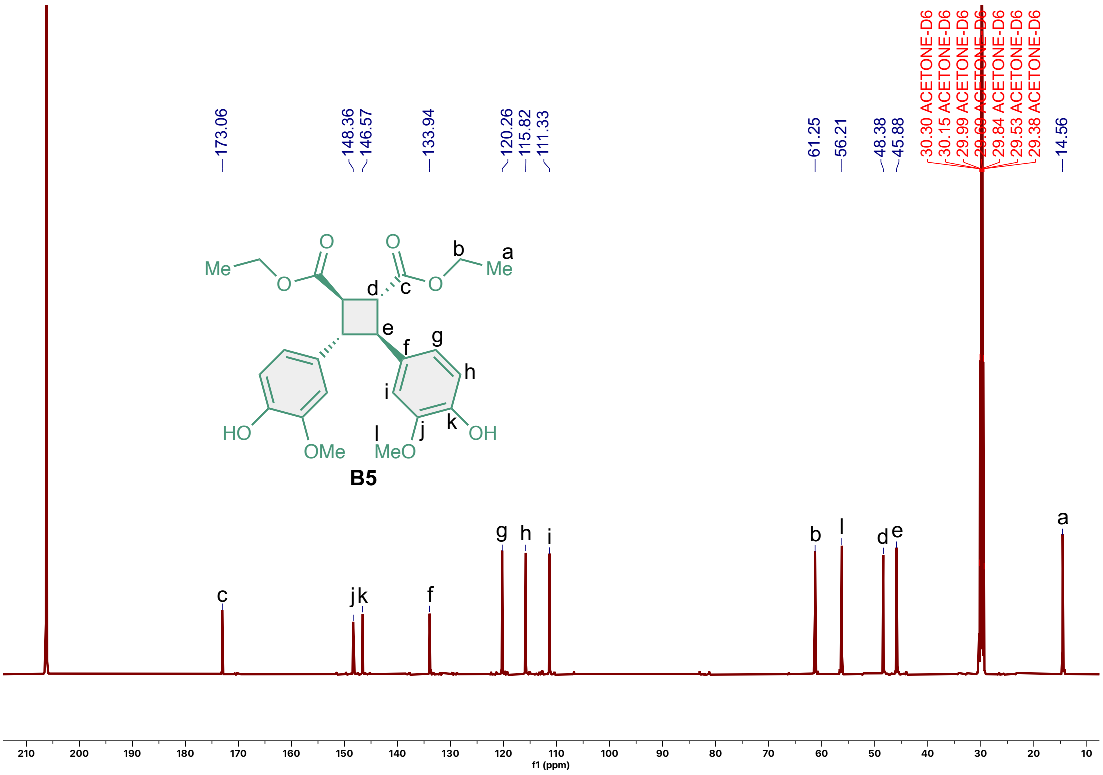

Figure S62. ^{13}C NMR spectrum of **B5** [126 MHz, ACETONE-D_6].

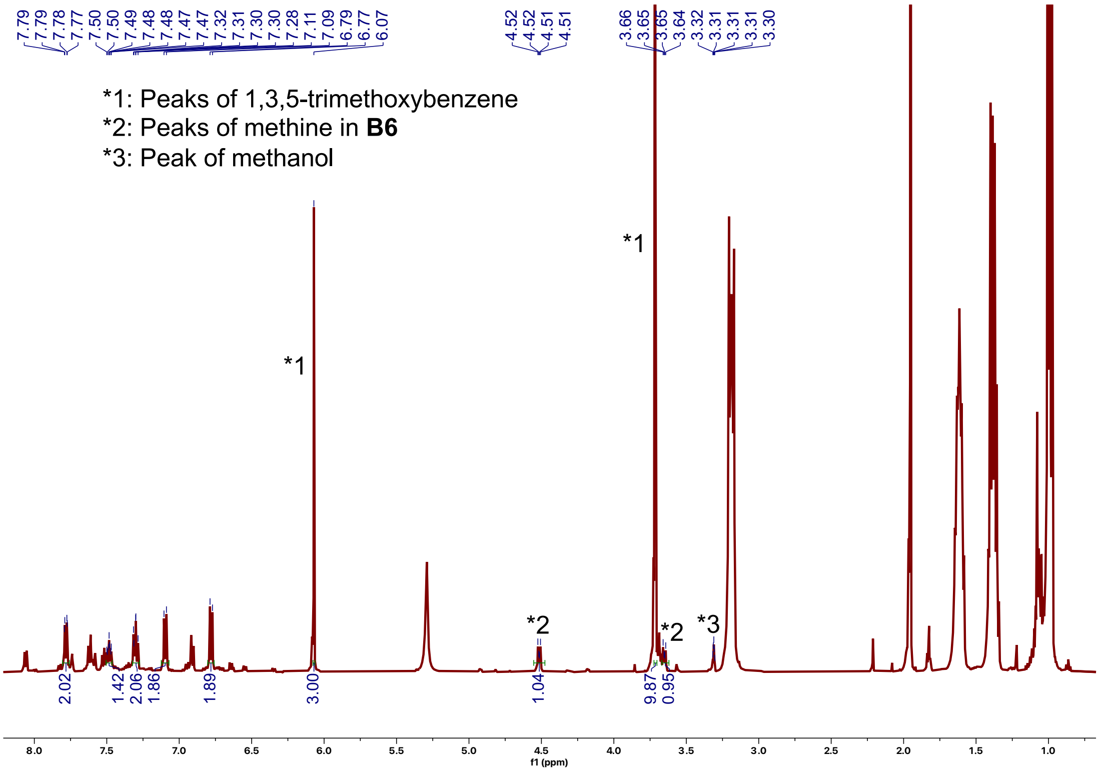

Figure S63. ^{1}H NMR spectrum after the decomposition of **P6** with 1,3,5-trimethoxybenzene as an internal standard. [500 MHz, METHANOL-D_4].

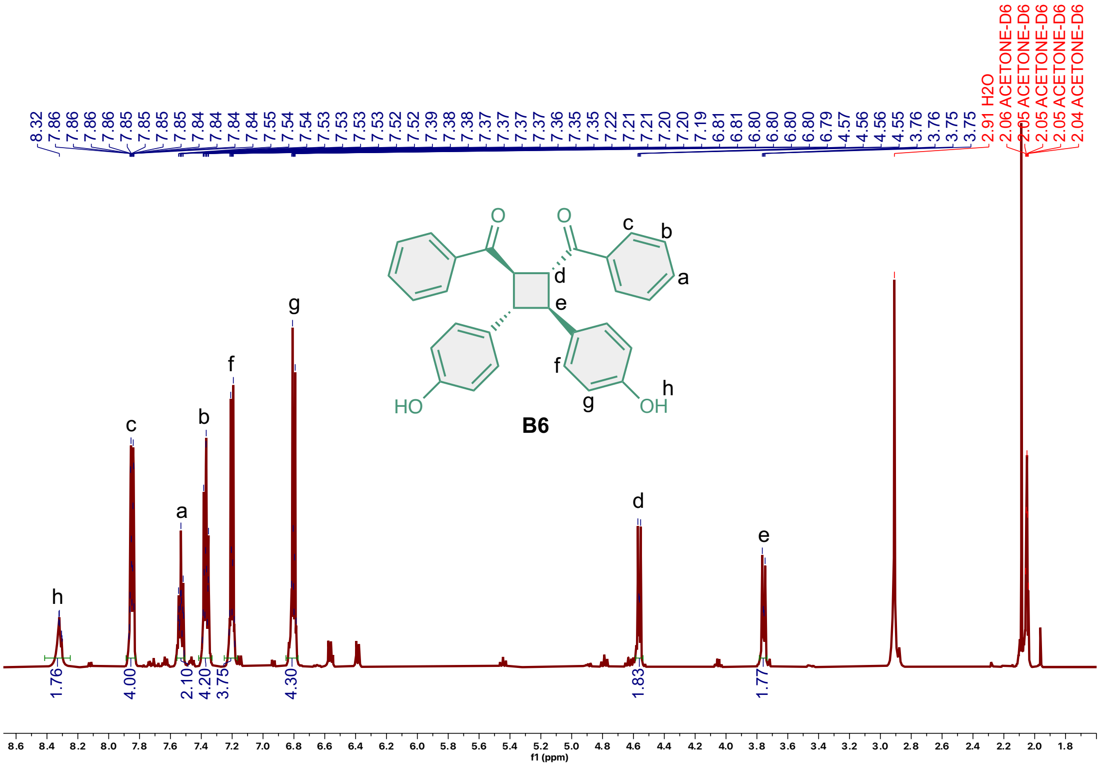

Figure S64. ^{1}H NMR spectrum of **B6** [500 MHz, ACETONE-D_6].

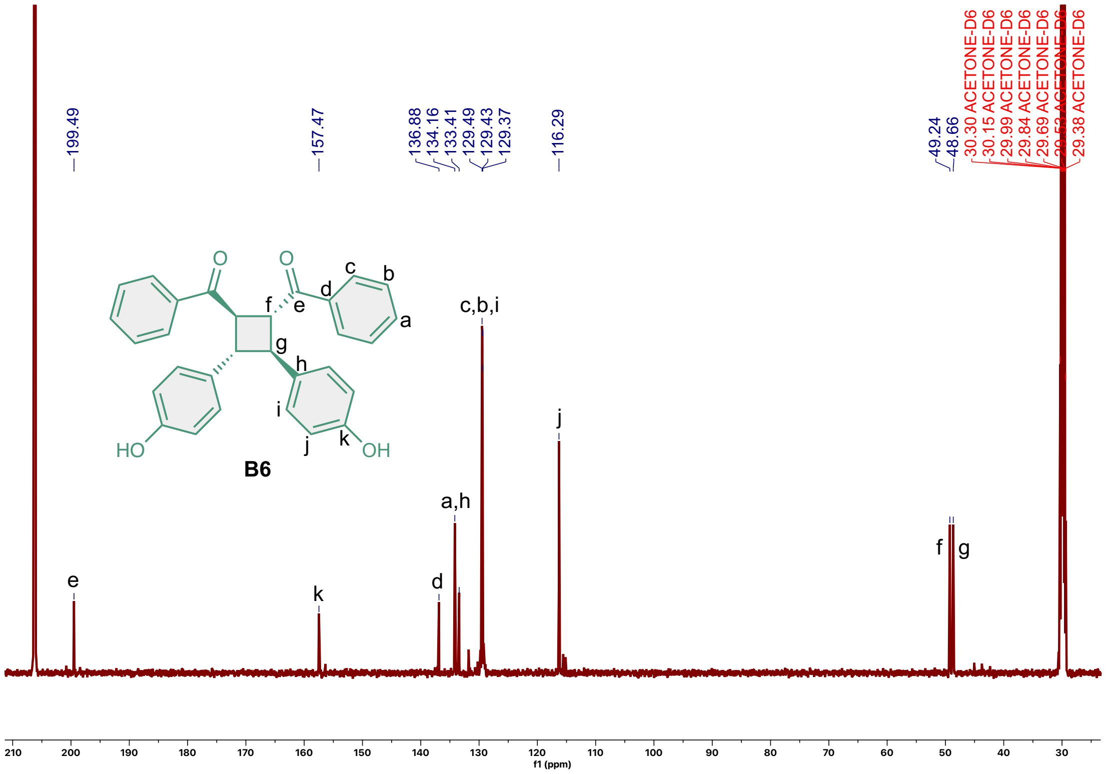

Figure S65. ^{13}C NMR spectrum of **B6** [126 MHz, ACETONE-D_6].

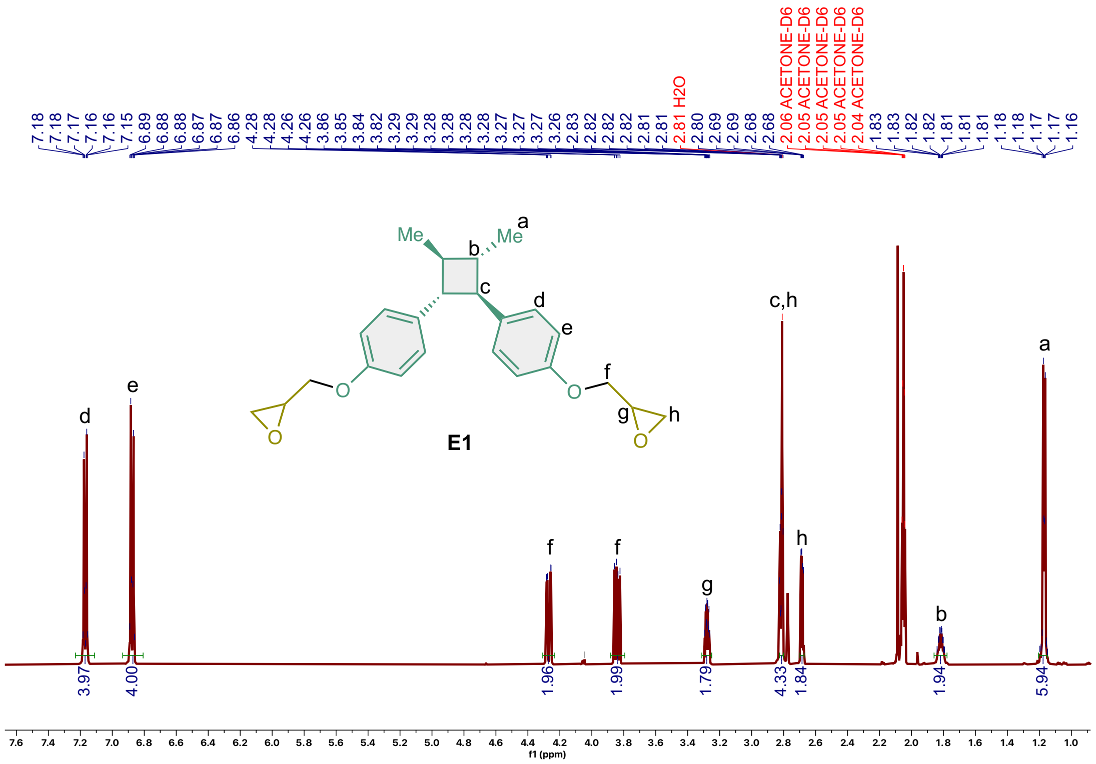

Figure S66. ^{1}H NMR spectrum of **E1** [500 MHz, ACETONE-D_6].

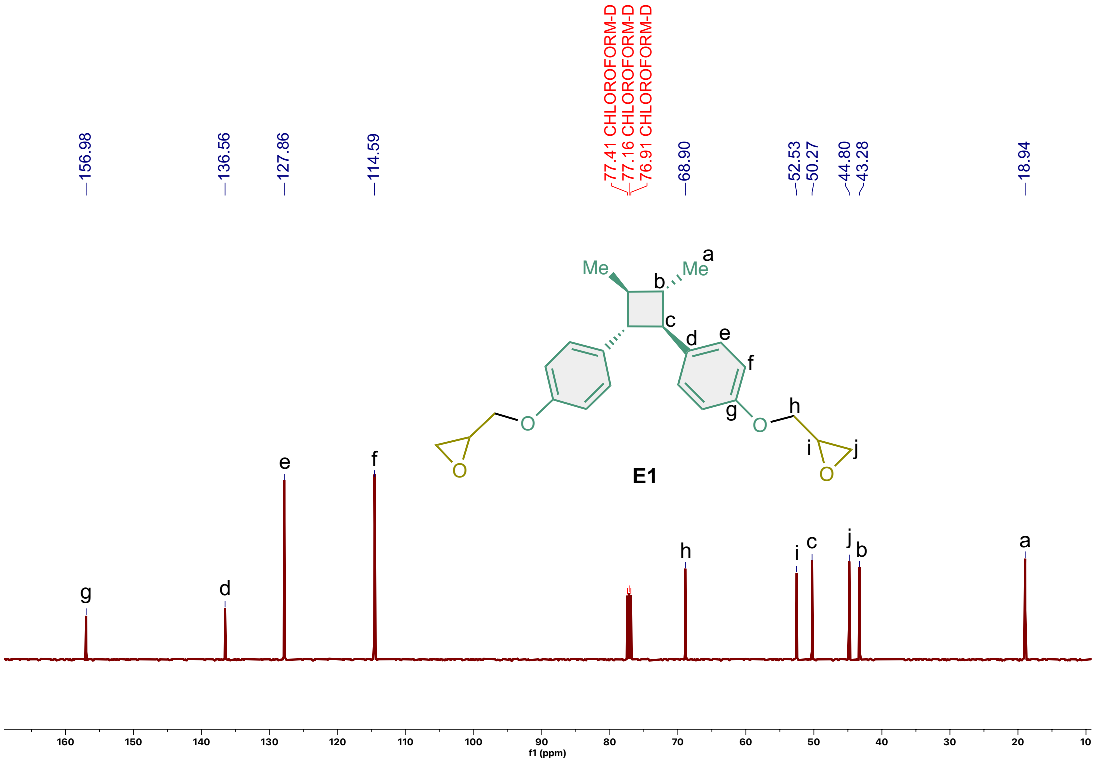

Figure S67. ^{13}C NMR spectrum of **E1** [126 MHz, CHLOROFORM-*D*].

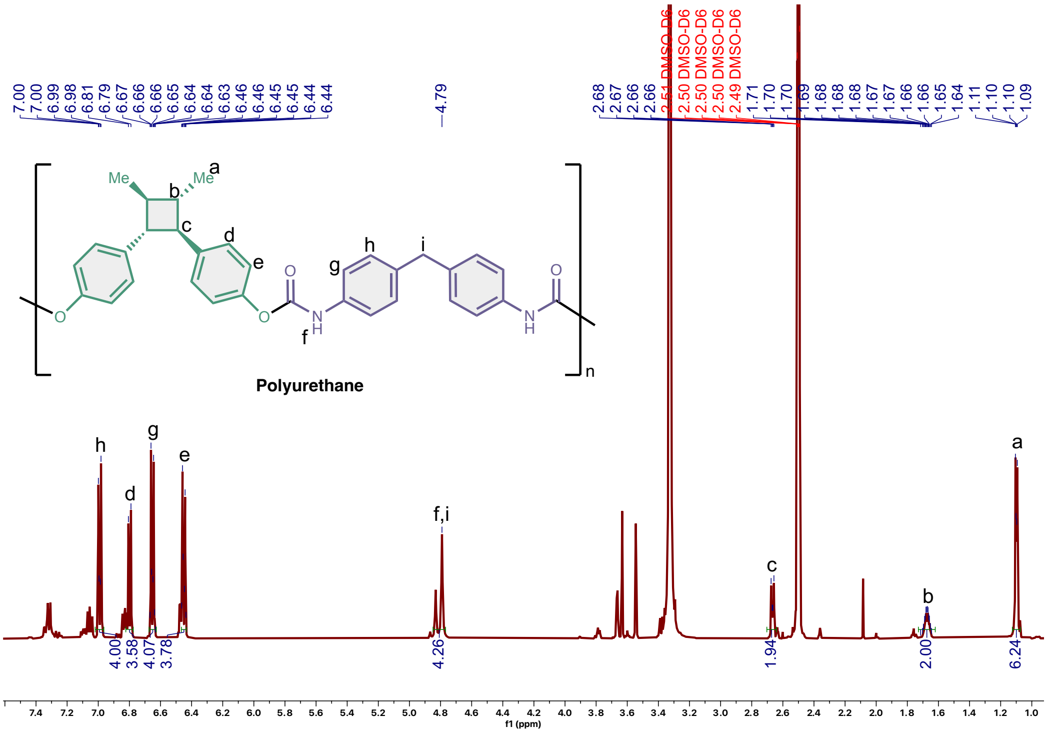

Figure S68. ^{1}H NMR spectrum of **polyurethane** [500 MHz, DMSO-D_6].

The slight peak disorder in the aromatic region, etc., may be due to the reaction of the terminal isocyanate groups of the polymer with DMSO.

6. HRMS Data

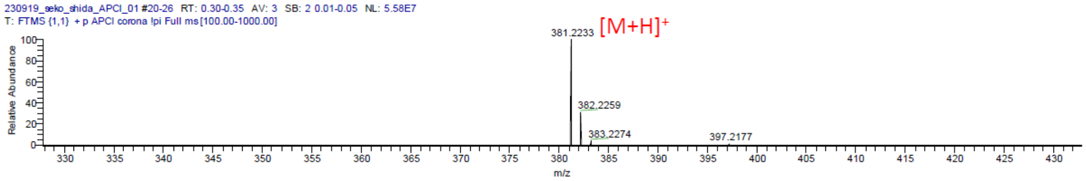

Figure S69. HRM (APCI) spectrum of **M1**.

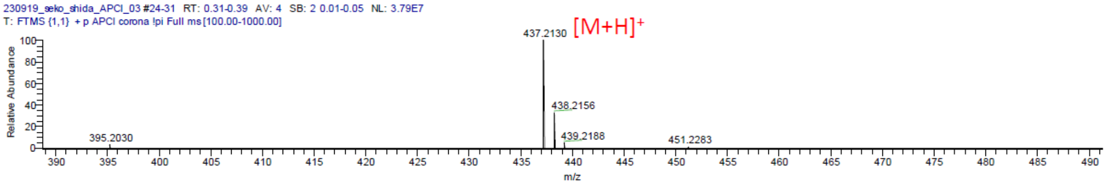

Figure S70. HRM (APCI) spectrum of **M2**.

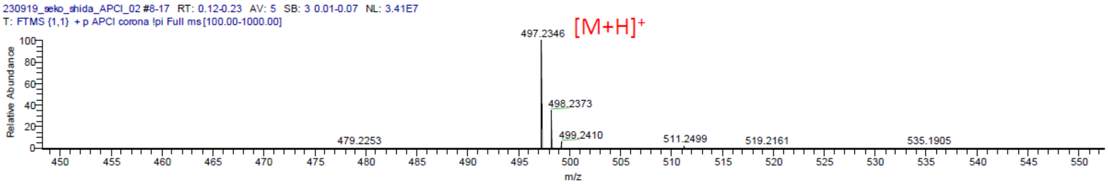

Figure S71. HRM (APCI) spectrum of **M3**.

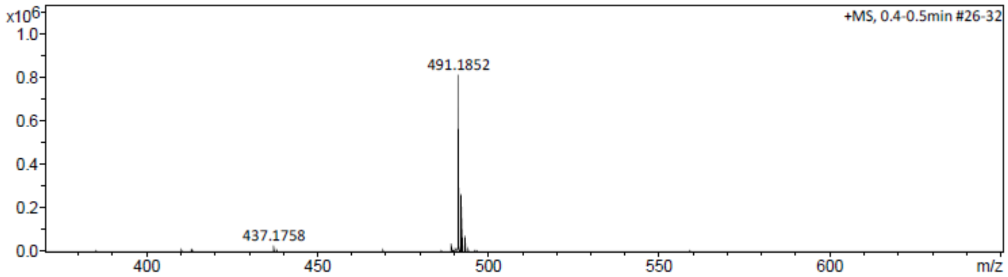

Figure S72. HRM (ESI) spectrum of **M4**.

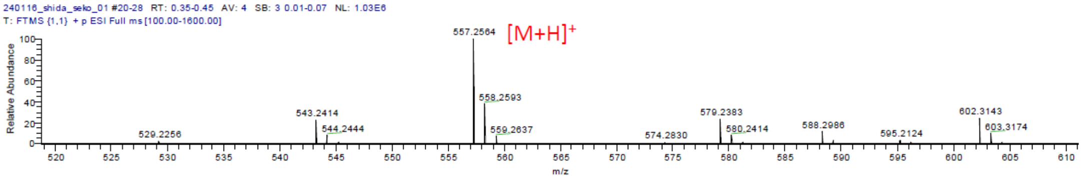

Figure S73. HRM (ESI) spectrum of **M5**.

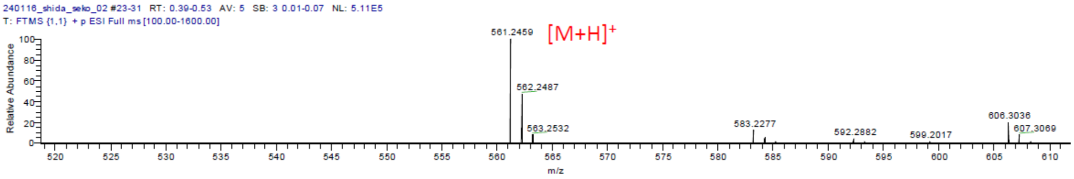

Figure S74. HRM (ESI) spectrum of **M6**.

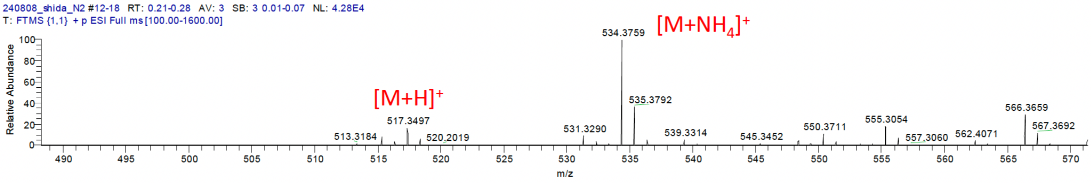

Figure S75. HRM (ESI) spectrum of **D1**.

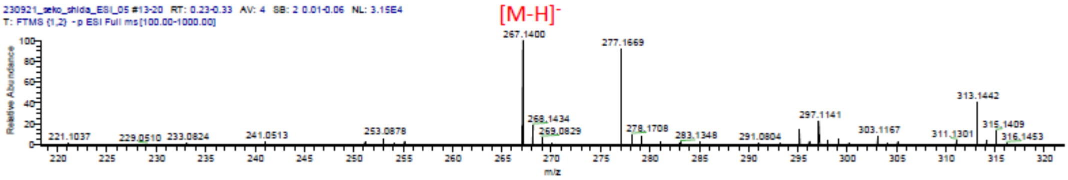

Figure S76. HRM (ESI) spectrum of **B1**.

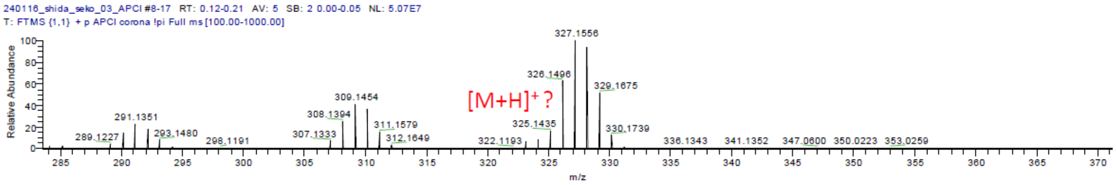

Figure S77. HRM (APCI) spectrum of **B2**.

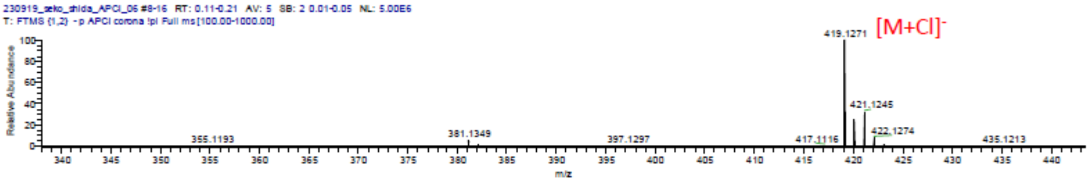

Figure S78. HRM (APCI) spectrum of **B3**.

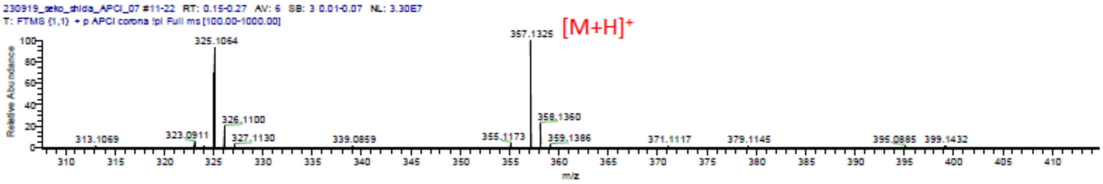

Figure S79. HRM (APCI) spectrum of **B4**.

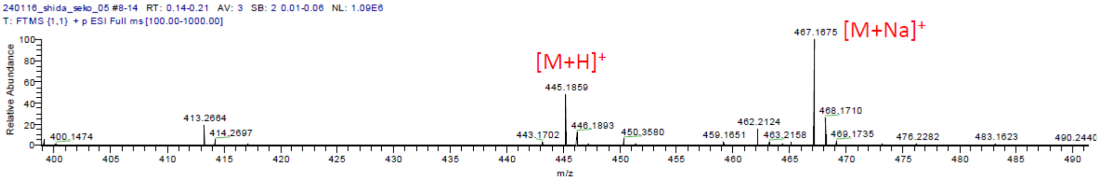

Figure S80. HRM (ESI) spectrum of **B5**.

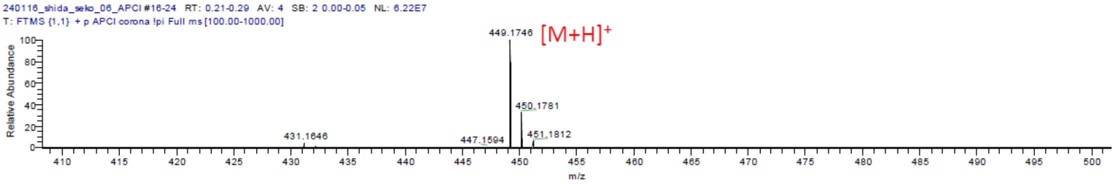

Figure S81. HRM (APCI) spectrum of **B6**.

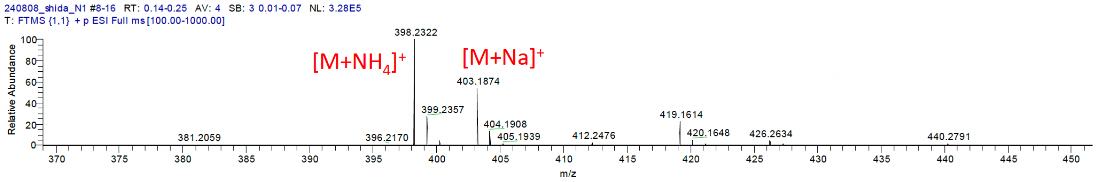

Figure S82. HRM (ESI) spectrum of **E1**.

7. Supplementary References

1. Church, A. C.; Pawlow, J. H. & Wagener, K. B. An examination of the substitution chemistry of di-*n*-hexyldichlorosilane. *J. Org. Chem.* **620**, 287-295 (2001).
2. Shi, M.; Huang, G.; Sun, J. & Fang, Q. Constructing low-*k* polymers at high frequency from two propenyl-containing biomasses through the grubbs reaction. *Polym. Chem.* ***14***, 999–1006 (2023).
3. Aplin, J. T. & Bauld, N. L. Mechanistic distinctions between cation radical and carbocation propagated polymerization. *J. Org. Chem.* **63**, 2586-2590 (1998).
4. Colomer, I., Batchelor-McAuley, C., Odell, B., Donohoe, T. J. & Compton, R. G. Hydrogen bonding to hexafluoroisopropanol controls the oxidative strength of hypervalent iodine reagents. *J. Am. Chem. Soc.* **138**, 8855–8861 (2016).
5. Koike, T. & Akita, M. Visible-light radical reaction designed by Ru- and Ir-based photoredox catalysis. *Inorg. Chem. Front.* **1**, 562-576 (2014).
